# Identification of a Novel Gene Involved in Cell-to-cell Communication-induced Cell Death and eDNA Production in *Streptococcus mutans*

**DOI:** 10.1264/jsme2.ME22085

**Published:** 2023-06-10

**Authors:** Ryo Nagasawa, Nobuhiko Nomura, Nozomu Obana

**Affiliations:** 1 Graduate School of Life and Environmental Sciences, University of Tsukuba, Japan; 2 Faculty of Life and Environmental Sciences, University of Tsukuba, Japan; 3 Microbiology Research Center for Sustainability, University of Tsukuba, Japan; 4 Faculty of Medicine, Transborder Medical Research Center, University of Tsukuba, Japan

**Keywords:** subpopulation ana­lysis, cell death, eDNA production, biofilm, *Streptococcus mutans*

## Abstract

*Streptococcus mutans* is a major caries-causing bacterium that forms firmly attached biofilms on tooth surfaces. Biofilm formation by *S. mutans* consists of polysaccharide-dependent and polysaccharide-independent processes. Among polysaccharide-independent processes, extracellular DNA (eDNA) mediates the initial attachment of cells to surfaces. We previously reported that the secreted peptide signal, competence-stimulating peptide (CSP) induced cell death in a subpopulation of cells, leading to autolysis-mediated eDNA release. The autolysin gene *lytF*, the expression of which is stimulated by CSP, has been shown to mediate CSP-dependent cell death, while cell death was not entirely abolished in the *lytF* deletion mutant, indicating the involvement of other factors. To identify novel genes involved in CSP-dependent cell death, we herein compared transcriptomes between live and dead cells derived from an isogenic population. The results obtained revealed the accumulation of several mRNAs in dead cells. The deletion of *SMU_1553c*, a putative bacteriocin gene, resulted in significant reductions in CSP-induced cell death and eDNA production levels from those in the parental strain. Moreover, in the double mutant strain of *lytF* and *SMU_1553c*, cell death and eDNA production in response to synthetic CSP were completely abolished under both planktonic and biofilm conditions. These results indicate that SMU_1553c is a novel cell death-related factor that contributes to CSP-dependent cell death and eDNA production.

*Streptococcus mutans* is a major bacterium that causes dental caries by forming firmly attached biofilms on tooth surfaces. Biofilms comprise bacterial cells and an extracellular matrix (Flemming and Wingender, 2010). The extracellular matrix of biofilms formed by *S. mutans* mainly consists of polysaccharides and extracellular DNA (eDNA) (Bowen and Koo, 2011; Klein *et al.*, 2015). Biofilm studies on *S. mutans* have focused on the polysaccharide-dependent mechanism induced by sucrose (Bowen and Koo, 2011; Koo *et al.*, 2013). However, eDNA has recently been attracting attention as a target to inhibit biofilm formation (Okshevsky *et al.*, 2015). Accumulating evidence suggests that eDNA contributes to biofilm formation in *S. mutans* (Petersen *et al.*, 2005; Das *et al.*, 2010; Kawarai *et al.*, 2016; Jung *et al.*, 2017; Nagasawa *et al.*, 2020a, 2020b). Therefore, a more detailed understanding of the regulatory mechanisms underlying eDNA production is important for controlling cariogenic biofilms.

Autolysis-dependent and membrane vesicle-mediated mechanisms that produce eDNA have been reported in *S. mutans* (Dufour and Lévesque, 2013; Liao *et al.*, 2014; Jung *et al.*, 2017). We previously detected autolysis-mediated eDNA production in a subpopulation of cells, which was induced by the Com system (Nagasawa *et al.*, 2020b). Although this eDNA production may be attributed to cell death, observations of several dead cells encapsulating nucleic acids (Nagasawa *et al.*, 2020b) suggested that not all dead cells were lysed to produce eDNA. The Com system of *S. mutans* is regulated by cell-to-cell communication via two secreted peptide signals: competence-stimulating peptide (CSP) and SigX-inducing peptide (XIP) (Mashburn-Warren *et al.*, 2010; Reck *et al.*, 2015; Khan *et al.*, 2016; Kaspar *et al.*, 2017). This system was initially reported as a hierarchical gene regulatory system that induces genetic competence and was later shown to control eDNA production via autolysis (Petersen and Scheie, 2000; Dufour and Lévesque, 2013). LytF, an autolysin whose expression is controlled by this system, is a major factor contributing to the autolysis-mediated production of eDNA (Dufour and Lévesque, 2013). Genes under the control of the Com system are classified into the ComE, ComR, and SigX regulons (Khan *et al.*, 2016). SigX is a sigma factor located the most downstream of the regulatory hierarchy, and it directly activates late competence genes, autolysin, and other genes (Khan *et al.*, 2016). Therefore, this sigma factor regulates the ability to take up exogenous DNA and produce eDNA. The Com system of *S. mutans* may be induced by the external addition of synthetic CSP (sCSP) or synthetic XIP (sXIP), depending on culture conditions. In a complex medium, sCSP, but not sXIP, induced the expression of *sigX* in a subpopulation of cells, and *lytF* was coexpressed with *sigX* (Lemme *et al.*, 2011; Son *et al.*, 2012; Reck *et al.*, 2015). On the other hand, when cultured in a chemically defined medium, sXIP induces *sigX* expression in a whole population of cells, while sCSP is unable to induce *sigX* expression (Reck *et al.*, 2015). These differences in cell responsiveness are attributed to the peptide content of the culture medium (Son *et al.*, 2012; Hagen and Son, 2017; Underhill *et al.*, 2019).

In our previous study using a complex medium, we found that sCSP induced cell death in a subpopulation of cells that localized at the bottom of the biofilm, contributing to cell-to-surface adherence (Nagasawa *et al.*, 2020b). The dead cell population was mainly found in the *lytF*-expressing subpopulation. However, the deletion of *lytF* partially suppressed cell death and eDNA production. This finding suggests that *lytF* is required for the maximum efficiency of cell death and subsequent eDNA production, but is not the sole determinant of sCSP-induced cell death. Since sCSP also induces natural competence-related genes, living cells are predicted to be competent cells that incorporate foreign DNA, while dead cells are a source of eDNA. The incorporation of foreign DNA results in increased diversity within the species through the acquisition of new traits, and eDNA production leads to robust biofilm formation and tolerance to external stresses. Therefore, the fate determination of life or death in the presence of CSP may have important implications for the survival strategy of *S. mutans*. However, the genetic components involved in cell death in *S. mutans* remain elusive.

To elucidate the mechanisms underlying eDNA production induced by CSP, we herein attempted to identify novel genes involved in cell death. We combined live/dead cell sorting with a transcriptome ana­lysis, which indicated the accumulation of mRNAs in dead cells. Experiments with the mutant strains revealed a novel cell death-related factor.

## Materials and Methods

### Bacterial strains and culture conditions

The bacterial strains used in the present study are listed in Table‍ ‍1. We cultured these strains in brain heart infusion (BHI) broth (Difco Laboratories) in an aerobic atmosphere containing 5% CO_2_ at 37°C. To induce the Com system, we used sCSP (SGSLSTFFRLFNRSFTQA; 18 amino acids) at a final concentration of 1‍ ‍μM.

### Flow cytometry and cell sorting

Overnight cultures were diluted to an OD_600_ of 0.05 with fresh BHI or BHI supplemented with sCSP (BHI sCSP) and incubated in an aerobic atmosphere containing 5% CO_2_ at 37°C for 6 h. Dead cells were stained with 500 nM SYTOX Green (Thermo Fisher Scientific) for 15‍ ‍min. Samples were diluted with sterile phosphate-buffered saline (PBS) where appropriate. Live and dead cells were analyzed and separated by a cell sorter (SH800Z; Sony). SYTOX Green was excited with a 488‍ ‍nm laser, and fluorescence was detected with 525/±25 and 487.5 LP filters. mScarlet-I was excited with a 561‍ ‍nm laser, and fluorescence was detected with 600/±30 and 561 LP filters. The boundary between positive and negative fluorescence was set using unstained WT cells. We confirmed whether cells were separated correctly by reanalyzing each collected cell. Samples were maintained at 5°C during cell sorting. A total of 10^7^ live and dead cells were collected. Collected cells were concentrated by entrapment on membrane filters (0.22-μm Triton-free mixed cellulose ester membranes; Merck).

### Total RNA extraction and RNA-seq ana­lysis

To extract total RNA from the cells collected by cell sorting, we placed membrane filters with trapped cells in 15-mL tubes containing 500‍ ‍μg of glass beads, 1‍ ‍mL of LETS buffer (0.1 M LiCl, 10‍ ‍mM Tris-HCl, 10‍ ‍mM EDTA, and 1% SDS), and 1‍ ‍mL of phenol‒chloroform-isoamyl alcohol (PCI; Nippon Gene), and disrupted cells by bead beating with a vortex mixer for 4‍ ‍min. After centrifugation, the aqueous phase was transferred to a new microtube and mixed with an equal volume of PCI. After centrifugation, the aqueous phase was transferred to a new microtube, and nucleic acids were precipitated using isopropanol and sodium acetate. The pellet was washed with 70% ethanol and treated with DNase I (Roche Diagnostics). After treatment, DNase I was denatured and removed by a phenol‒chloroform treatment. Total RNA was obtained by ethanol precipitation. The quantity and quality of the total RNA extracted were confirmed using the RiboGreen assay (Quant-iT RiboGreen RNA Assay Kit; Invitrogen) and reverse transcription-PCR (RT-PCR), respectively.

Before preparing the cDNA library for RNA-seq, ribosomal RNA was depleted using the NEBNext rRNA Depletion Kit (New England Biolabs). Transcripts were fragmented and used as a template to generate a strand-specific cDNA library using the TruSeq Stranded Total RNA Library Prep kit (Illumina). Samples were sequenced using 100-bp paired-end reads with a NovaSeq 6000 sequencer system (Illumina). The reads obtained were trimmed and mapped to the reference genome of *S. mutans* UA159 (accession number: AE014133) using Geneious (Biomatters) to identify genes with altered expression. Since the purpose of RNA-seq is to screen candidate genes, the trial number was one.

### RNA-seq data accession number

The RNA-seq data obtained in the present study have been deposited in the DNA Data Bank of Japan (DDBJ) Sequence Read Archive (DRA) and may be referenced under accession number DRA014393.

### RT‒PCR

Overnight cultures were diluted to an OD_600_ of 0.05 with fresh BHI or BHI with sCSP and incubated in an aerobic atmosphere containing 5% CO_2_ at 37°C for 2, 4, or 6 h. Total RNA was extracted and quantified by the method described above. We synthesized cDNA from 500‍ ‍ng of total RNA with the PrimeScript™ RT reagent kit with a gDNA eraser (Takara). The primers used for RT‒PCR are shown in Table S1. PCR was performed with Tks Gflex DNA polymerase (Takara) for 25 cycles. We used the lactate dehydrogenase gene *ldh*, a housekeeping gene in *S. mutans*, as an endogenous control (Merritt *et al.*, 2005). PCR products were electrophoresed in a 1.5% agarose gel and stained with Midori Green Xtra (Nippon Genetics).

### Construction of mutant strains

The primers used to construct the deletion mutant strains in the present study are shown in Table S2. Sequence information was obtained from the KEGG (http://www.genome.jp/kegg/) and NCBI (https://www.ncbi.nlm.nih.gov/) databases. Deletion mutants were constructed by replacing the target gene with an erythromycin resistance gene (*ermBP*), kanamycin resistance gene (*aph3*), or spectinomycin resistance gene (*aad9*). The upstream and downstream sequences of the target genes were amplified from the genomic DNA of *S. mutans* UA159 WT by PCR. *ermBP*, *aph3*, and *aad9* were amplified from pJIR418 and from the genomic DNA of *Bacillus subtilis* TAY3203 and pDL278, respectively (LeBlanc *et al.*, 1992; Sloan *et al.*, 1992; Yamamoto *et al.*, 2014). DNA fragments were linked by overlap extension PCR and introduced into competent cells of WT *S. mutans* UA159. Competent cells were prepared by adding sCSP at a final concentration of 1‍ ‍μM to cells grown to the early-log phase in BHI and then cultured for 2 h. Transformants were screened in Mitis Salivarius agar (Difco Laboratories) plates with erythromycin (10‍ ‍μg mL^–1^), kanamycin (900‍ ‍μg mL^–1^), or spectinomycin (200‍ ‍μg mL^–1^). The insertion of the mutation into the target locus was confirmed by colony PCR and DNA sequencing.

The *SMU_1553c*-complemented strain (*SMU_1553c* comp) was constructed by inserting the native promoter and open reading frame (ORF) into the pseudogene locus *SMU_437c*. The primers used to construct *SMU_1553c* comp are shown in Table S3. The upstream and downstream sequences of *SMU_437c*, including the kanamycin resistance gene, were amplified by PCR from the genomic DNA of the WT P*_lytF_* reporter of *S. mutans* (Nagasawa *et al.*, 2020b). The sequence from the promoter of *SMU_1554c* to the *SMU_1553c* ORF was amplified by PCR from the genomic DNA of WT *S. mutans* UA159. DNA fragments were linked by overlap extension PCR and introduced into competent cells of the Δ*SMU_1553c* strain. Genetic competence was induced by the method described above, and transformants were screened on MS plates with kanamycin (900‍ ‍μg mL^–1^). The DNA fragment inserted at the target locus was confirmed by colony PCR and DNA sequencing.

### eDNA quantification

Overnight cultures were diluted to an OD_600_ of 0.05 with fresh BHI or BHI sCSP and incubated in an aerobic atmosphere containing 5% CO_2_ at 37°C for 6 h. At the endpoint, OD_600_ was measured by a spectrophotometer (NanoDrop2000c; Thermo Scientific). Extracellular nucleic acids were purified from culture supernatants. Culture supernatants were mixed with an equal volume of cetyltrimethylammonium bromide and incubated at 65°C for 15‍ ‍min. After mixing with an equal volume of PCI, the sample was centrifuged. Nucleic acids in the aqueous phase were precipitated using isopropanol and sodium acetate. The pellet was washed with 70% EtOH and dissolved in TE buffer (10‍ ‍mM Tris-HCl and 1‍ ‍mM EDTA). We used the Quant-iT PicoGreen dsDNA assay kit (Thermo Fisher Scientific) to quantify double-stranded DNA. The results obtained were standardized by the OD_600_ of the culture solution at 6 h.

### Biofilm formation

Overnight cultures were diluted to an OD_600_ of 0.05 with fresh BHI supplemented with 1% (w/v) sucrose (BHIs) or BHIs supplemented with 1‍ ‍μM sCSP (BHIs sCSP). Samples were placed into a glass-bottom dish (Matsunami) and incubated in an aerobic atmosphere containing 5% CO_2_ at 37°C for 6 h. After the incubation, cell cultures were removed and the wells were washed twice with PBS to remove planktonic cells. We stained all cells with 5‍ ‍μM SYTO 59 (Thermo Fisher Scientific) and dead cells and extracellular nucleic acids with 1.25‍ ‍μM SYTOX Green (Thermo Fisher Scientific) for 30‍ ‍min. Stained biofilms were washed twice with PBS before observations.

### Confocal laser scanning microscopy (CLSM)

Biofilm cells were observed using an inverted confocal laser scanning microscope (LSM780; Carl Zeiss) with a C-Apochromat 40×/1.2 water-immersion objective lens. Z-stacks were acquired at 0.5-μm intervals. SYTOX Green and SYTO 59 were excited by 488 and 633 nm lasers, respectively, and emissions were detected at 499 to 535‍ ‍nm for SYTOX Green and 642 to 695‍ ‍nm for SYTO 59.

### Statistics

Differences between mean values for multiple groups were analyzed by a one-way ana­lysis of variance with Tukey’s honestly significant difference test (IBM SPSS statistics 25, IBM Corporation). In the present study, three independent experiments were performed in triplicate. *P*<0.05 was considered to be significant.

## Results and Discussion

### RNA-seq ana­lysis of live and dead cells as a screening method for cell death-related factors

In *S. mutans*, cell death was induced in a subpopulation of cells when they were cultured in a complex medium containing sCSP (Lemme *et al.*, 2011; Son *et al.*, 2012; Reck *et al.*, 2015). We previously showed using a flow cytometry ana­lysis that cell death by sCSP mainly occurred in the *lytF*-expressing population. However, the deletion of *lytF* did not entirely abolish CSP-induced cell death, suggesting that *lytF* is not the sole factor triggering cell death (Nagasawa *et al.*, 2020b). In the present study, to clarify the factors contributing to the life or death of cells, we attempted a comprehensive comparison of gene expression in live and dead cells of *S. mutans*. We cultured *S. mutans* in BHI sCSP, labeled dead cells in the population with SYTOX Green, and separated live and dead cells using a cell sorter. Before sorting, the bacterial population contained 85% live cells and 15% dead cells (Fig. S1A). After cell sorting based on SYTOX Green fluorescence, collected cells were reanalyzed by flow cytometry, confirming the enrichment of live or dead cells (Fig. S1B and C). We concluded that each target cell population was sufficiently enriched for the subsequent RNA-seq ana­lysis.

Based on RNA-seq results, we extracted mRNAs differentially expressed between live and dead cells (Table S4 and Fig. S2). To validate RNA-seq results, we initially confirmed whether known cell death-related genes were highly expressed in dead cell-derived RNAs. *cipB* is a bacteriocin-encoding gene in the ComE regulon, and the intracellular accumulation of CipB leads to cell death in *S. mutans* (Perry *et al.*, 2009; Khan *et al.*, 2016). CipI is an immunity protein against CipB, and the expression balance between CipB and CipI is considered to be important for cell survival (Perry *et al.*, 2009; Dufour *et al.*, 2011). Our RNA-seq results showed that *cipB* mRNA levels were 3.76-fold higher in dead cells than in live cells (Table S4). On the other hand, the mRNA levels of the immunity protein CipI were 3.95-fold lower in dead cells than in live cells (Table S4). In addition, the mRNA levels of *lrgB*, which encodes one of the factors involved in the autolysis of *S. mutans* (Ahn *et al.*, 2010; Ahn and Rice, 2016), were 8-fold higher in dead cells than in live cells (Table 2 and S4). Furthermore, the mRNA levels of *sigX*, which encodes a sigma factor and is necessary for cell death in a subpopulation, were more than four-fold higher in dead cells than in live cells (Table 2 and S4). It is important to note that *lytF* mRNA levels did not significantly differ between live and dead cells (Table S4). AtlA is another major autolysin in *S. mutans* whose expression is regulated independently of the Com system (Shibata *et al.*, 2005). AtlA contributes to eDNA production via cell death and to cell separation during cell division (Jung *et al.*, 2017; Zamakhaeva *et al.*, 2021). However, *atlA* mRNA did not accumulate in dead cells, which may have been due to the small impact of AtlA-dependent cell death under our experimental conditions. The percentage of dead cells of the Δ*atlA* strain in BHI sCSP was similar to that of WT (Fig. S3). Collectively, we concluded that our RNA-seq results were consistent with previous findings and may be used to identify novel cell death-related genes that were previously overlooked.

### *SMU_1553c* contributes to CSP-induced cell death and eDNA production

**Fig. 1.**
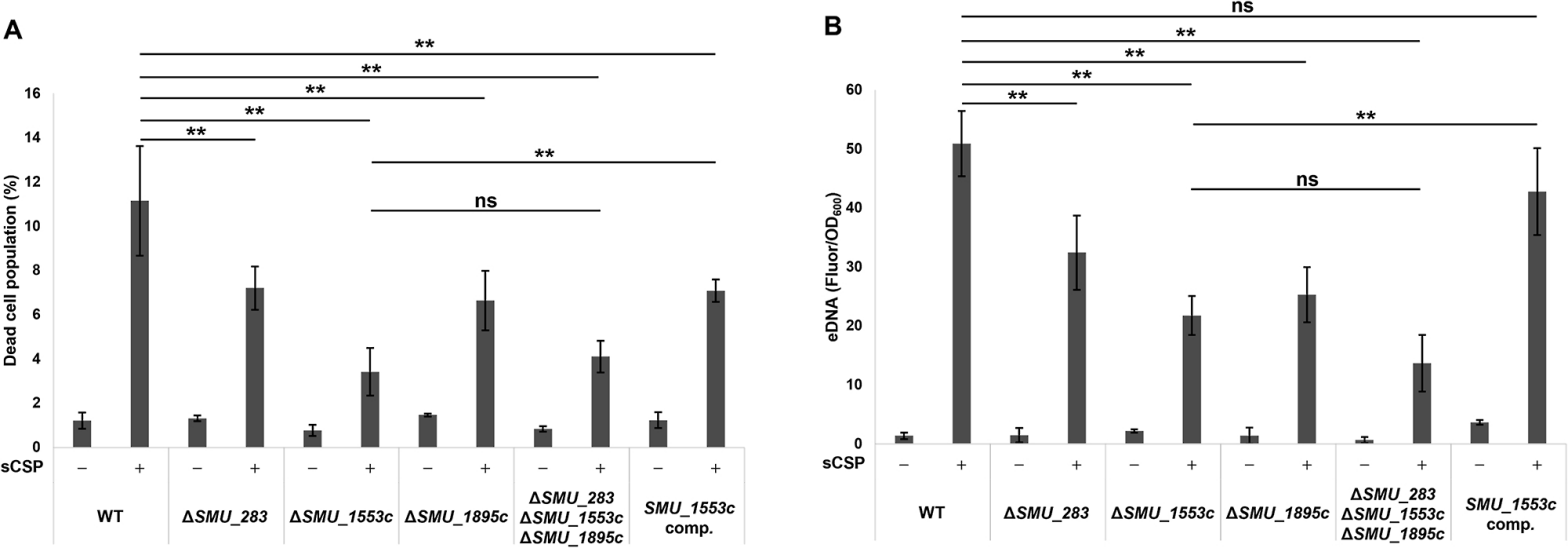
The deletion of *SMU_1553c* reduced sCSP-induced cell death and eDNA production Cells were grown in BHI with or without sCSP in an aerobic atmosphere containing 5% CO_2_ at 37°C for 6 h. (A) Dead cells were stained with SYTOX Green, and the population of SYTOX Green-positive cells was quantified by flow cytometry. (B) eDNA was extracted from 6-h cultures, and double-stranded DNA was quantified using the PicoGreen assay kit. Fluorescence values were standardized by the OD_600_ of the 6-h cultures. These data are presented as the mean±standard deviation of the results of three independent experiments. Asterisks indicate significant differences; **, *P*<0.01, and ns indicates not significant.

To identify genes involved in the induction of cell death, we focused on mRNAs that accumulated in dead cells. We identified 70 mRNAs with levels that were at least 4-fold higher in dead cells than in live cells, including mRNAs detected in dead cells only (Table 2). We performed the functional classification of the 70 differentially expressed genes using a gene ontology enrichment ana­lysis. However, more than 50% of the gene products were functionally unknown, and no noteworthy results were obtained to identify cell death-related factors. Since this study aimed to identify factors involved in cell death, we focused on proteins that appeared to act on the cell surface structure, particularly the plasma membrane. Therefore, we targeted *SMU_283*, *SMU_1553c*, and *SMU_1895c* because these genes are predicted to encode short peptide bacteriocins among the hypothetical proteins identified in the RNA-seq ana­lysis, and a detailed functional ana­lysis of their gene products was not conducted in previous studies (Hale *et al.*, 2005; van der Ploeg, 2005; Nicolas, 2011). We constructed the deletion mutants Δ*SMU_283*, Δ*SMU_1553c*, and Δ*SMU_1895c* and quantified the dead cell population and eDNA production in these mutants. All of the single mutant strains showed a significant reduction in the cell death population and eDNA production induced by sCSP from those in WT (Fig. 1A and B). Among them, Δ*SMU_1553c* showed the most reduced dead cell population in the presence of sCSP, and this population size was similar to that of the triple mutant strain Δ*SMU_283 *Δ*SMU_1553c *Δ*SMU_1895c* (Fig. 1A). Since the triple mutant showed a smaller dead cell population size and lower eDNA production than those of the Δ*SMU_283* and Δ*SMU_1895c* single mutants, *SMU_1553c* was considered to exert the strongest effects on cell death and eDNA production. The complementation of *SMU_1553c* partially restored the dead cell population and eDNA production (Fig. 1A and B). Based on these results, *SMU_1553c* was a plausible candidate gene involved in the induction of cell death and subsequent eDNA production in response to sCSP.

Since RNA-seq results showed the low expression of *SMU_1553c*, we confirmed whether *SMU_1553c* was expressed by RT‒PCR. *SMU_1553c* expression was detected at all of the time points tested (2, 4, and 6‍ ‍h), and time-dependent changes in expression levels were not observed (Fig. S4). Moreover, sCSP did not affect the expression of *SMU_1553c* (Fig. S4).

**Fig. 2.**
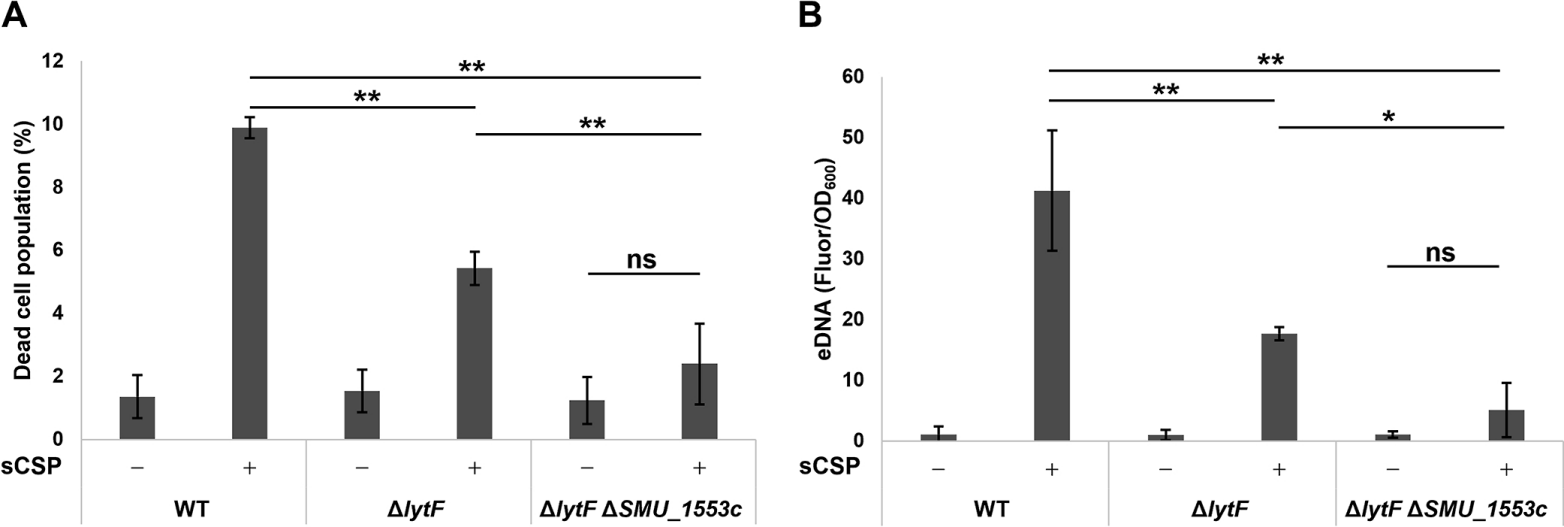
Double mutant strains of *lytF* and *SMU_1553c* did not induce cell death or eDNA production in response to sCSP Cells were grown in BHI with or without sCSP in an aerobic atmosphere containing 5% CO_2_ at 37°C for 6 h. (A) Dead cells were stained with SYTOX Green, and the populations of SYTOX Green-positive and SYTOX Green-negative cells were quantified by flow cytometry. (B) eDNA was extracted from 6-h cultures, and double-stranded DNA was quantified using the PicoGreen assay kit. Fluorescence values were standardized by the OD_600_ of the 6-h cultures. These data are presented as the mean±standard deviation of the results of three independent experiments. Asterisks indicate a significant difference; *, *P*<0.05, **, *P*<0.01, and ns indicates not significant.

We previously demonstrated that the induction of cell death and eDNA production in response to sCSP were lower in the *lytF* deletion mutant strain than in the WT strain (Nagasawa *et al.*, 2020b). However, the deletion of *lytF* alone did not completely abolish cell death or eDNA production. We then quantified the dead cell population and eDNA production in the *lytF* and *SMU_1553c* double mutant strain to examine the genetic epistasis of cell death and eDNA production. Δ*lytF* Δ*SMU_1553c* showed significantly larger decreases in number of dead cells and eDNA production induced by sCSP than Δ*lytF* (Fig. 2A and B). Furthermore, no significant differences were observed in the dead cell population or eDNA production between Δ*lytF* Δ*SMU_1553c* with or without sCSP (Fig. 2A and B). These results suggest that *SMU_1553c* is related to cell death and the eDNA production pathway in response to CSP. Although limited information is currently available on the *SMU_1553c*-encoded protein, a bioinformatics ana­lysis suggested that *SMU_1553c* was a part of the bacteriocin biosynthesis gene cluster (Nicolas, 2011). *SMU_1553c* encodes a 64-aa short peptide similar to carnocyclin A secreted by *Carnobacterium maltaromaticum*. Carnocyclin A is a bacteriocin that exhibits antibacterial activity against Gram-positive bacteria (Martin-Visscher *et al.*, 2008). However, our RNA-seq ana­lysis of dead cells indicated that *SMU_1553c* expression caused cell death, even though it is unclear whether the SMU_1553c peptide is secreted by these cells and it may have a different function from carnocyclin A.

**Fig. 3.**
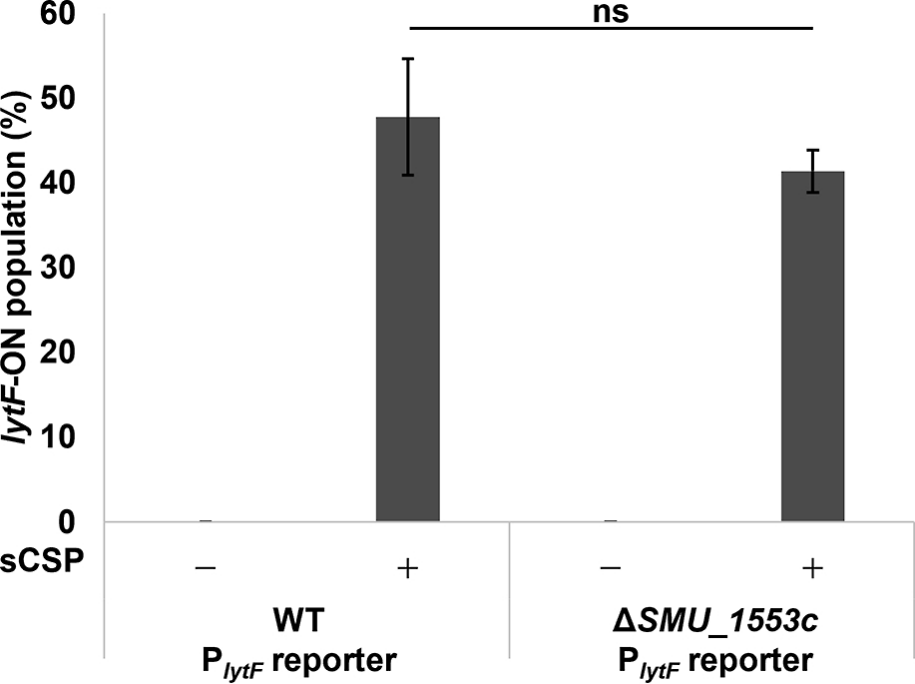
The deletion of *SMU_1553c* did not affect the percentage of *lytF*-ON cells P*_lytF_* reporter strains with a WT background (WT P*_lytF_* reporter) and Δ*SMU_1553c* background (Δ*SMU_1553c* P*_lytF_* reporter) were grown in BHI with or without sCSP in an aerobic atmosphere containing 5% CO_2_ at 37°C for 6 h. The population of *lytF*-ON was quantified by flow cytometry. Data are presented as the mean±standard deviation of the results of three independent experiments. ns indicates not significant.

Salivary mucin MUC5B up-regulates the expression of *SMU_1553c* and down-regulates the expression of the Com system (Werlang *et al.*, 2021). We speculated that the deletion of *SMU_1553c* affects *lytF* expression. The *lytF*-ON populations using the P*_lytF_* reporter system on a WT or Δ*SMU_1553c* background showed no significant differences (Fig. 3). Therefore, the deletion of *SMU_1553c* reduced CSP-induced cell death and subsequent eDNA production without affecting the expression of *lytF*.

### The deletion of *SMU_1553c* reduces cell death and eDNA production in biofilms

**Fig. 4.**
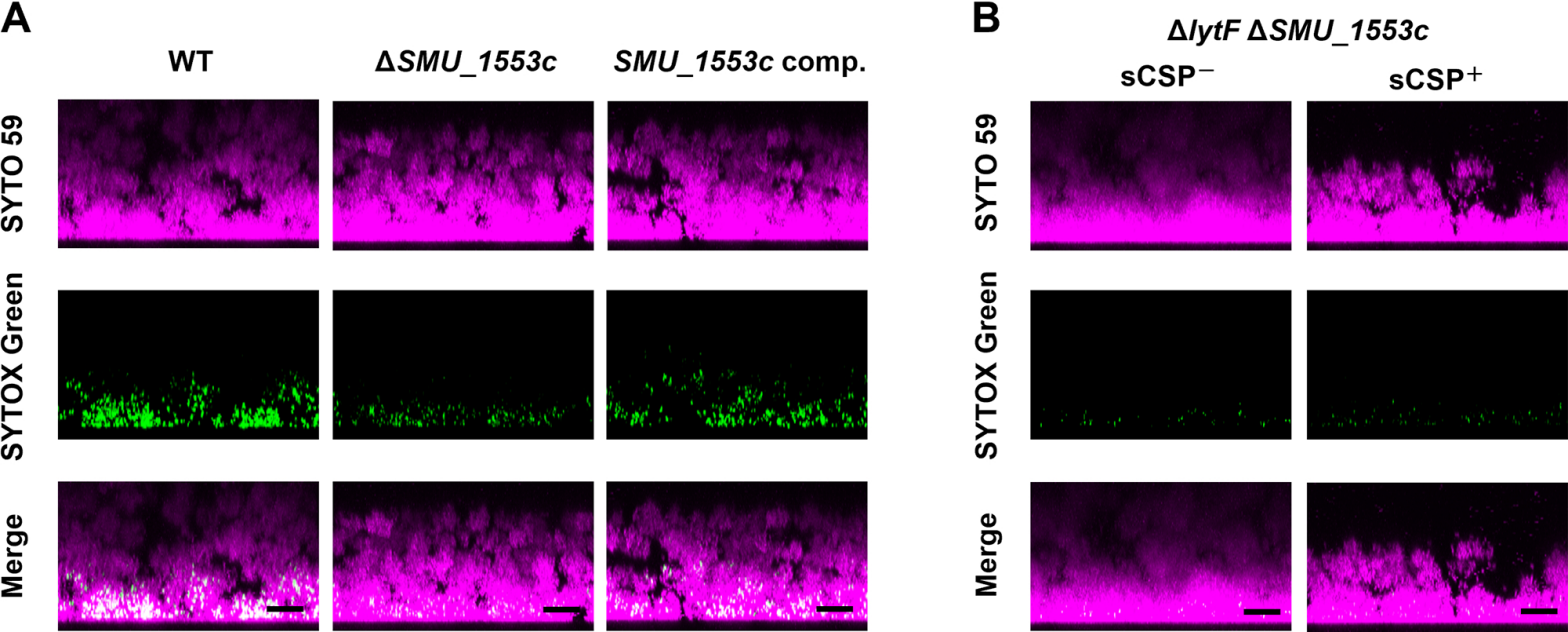
The deletion of *SMU_1553c* reduced cell death and eDNA production in biofilms Cells were grown in an aerobic atmosphere containing 5% CO_2_ at 37°C for 6‍ ‍h in glass-bottom dishes. After removing planktonic cells by washing twice with PBS, cells were stained with SYTO 59 and SYTOX Green. The entire cell population is indicated in magenta by SYTO 59 staining, and green indicates dead cells and extracellular nucleic acids stained with SYTOX Green. These images show the maximum intensity projection of the biofilm side view. Scale bars in merged images indicate 10‍ ‍μm. Representative images from three independent experiments are shown. (A) WT, Δ*SMU_1553c*, and *SMU_1553c* comp. strains were grown in BHIs with sCSP. (B) The Δ*lytF* & *SMU_1553c* strain was grown in BHIs with or without sCSP.

We previously reported that *lytF* was required to induce cell death and eDNA production near the bottom of a biofilm (Nagasawa *et al.*, 2020b). eDNA contributes to the initial attachment of a biofilm to surfaces, which is the first step in biofilm formation, and to its structural stability (Das *et al.*, 2010; Nagasawa *et al.*, 2017). We investigated whether the reductions in dead cells and eDNA production caused by the *SMU_1553c* deletion were also observed in biofilms. Δ*SMU_1553c* showed greater decreases in the number of dead cells and extracellular nucleic acid levels in biofilms in the presence of sCSP than WT (Fig. 4A). This phenotype was restored by the complementation of the *SMU_1553c* gene (Fig. 4A). Furthermore, dead cell numbers and extracellular nucleic acid levels were markedly reduced in the biofilms of the Δ*lytF* Δ*SMU_1553c* double mutant in the presence of sCSP and were similar to those in these biofilms in the absence of sCSP (Fig. 4B). Therefore, *SMU_1553c* appeared to affect the viability of *S. mutans* as an additional factor of CSP-dependent eDNA production.

In conclusion, we herein identified the novel gene, *SMU_1553c* that contributes to CSP-dependent cell death and eDNA production in biofilms by analyzing mRNAs that accumulated in a dead cell subpopulation. Cell death-related factors have primarily been identified through *in silico* homology searches and experimental screening. Homology searches reveal the presence and distribution of proteins with similar functions. Autolysin LytF in *S. mutans* has been focused on as a protein with a murein hydrolase domain and shown experimentally to be an autolysin (Dufour and Lévesque, 2013). On the other hand, AtlA in *S. mutans* is an autolysin identified by screening from a random mutant library (Shibata *et al.*, 2005). Moreover, a Tn-seq ana­lysis revealed a novel gene that contributes to cell death-mediated eDNA production in *Staphylococcus aureus* (DeFrancesco *et al.*, 2017). In contrast to these methods, our strategy does not require the construction of a mutant library. Therefore, our RNA-seq screening method has the advantage of being applicable to bacterial species for which there is no established method to construct a high-density random mutation library; however, it has some issues regarding the quality maintenance and processing of RNAs. *SMU_1553c* expression was previously shown to be induced by the host-derived biomolecule, salivary mucin MUC5B (Werlang *et al.*, 2021). Therefore, *SMU_1553c* may play a role in interactions with the host. A future challenge is to clarify gene expression regulation by and the detailed mechanism of action of *SMU_1553c* in cariogenic bacterial cell death.

## Citation

Nagasawa, R., Nomura, N., and Obana, N. (2023) Identification of a Novel Gene Involved in Cell-to-cell Communication-induced Cell Death and eDNA Production in *Streptococcus mutans*. *Microbes Environ ***38**: ME22085.

https://doi.org/10.1264/jsme2.ME22085

## Supplementary Material

Supplementary Material

## Figures and Tables

**Table 1.** Bacterial strains and plasmids

Strain or plasmid	Relevant properties	Source or reference
Strain		
*Streptococcus mutans*		
UA159	Wild type erm^S^ kan^S^ spec^S^	Ajdić *et al.*, 2002
Δ*lytF*	UA159 *lytF* deletion mutant; *ermBP*	Nagasawa *et al.*, 2020b
Δ*SMU_283*	UA159 *SMU_283* deletion mutant; *ermBP*	This study
Δ*SMU_1553c*	UA159 *SMU_1553c* deletion mutant; *ermBP*	This study
Δ*SMU_1895c*	UA159 *SMU_1895c* deletion mutant; *ermBP*	This study
Δ*SMU_283* Δ*SMU_1553c* Δ*SMU_1895c*	UA159 *SMU_283*, *SMU_1553c*, and *SMU_1895c* deletion mutant; *ermBP*, *aph3*, *aad9*	This study
Δ*atlA*	UA159 *atlA* deletion mutant; *ermBP*	Nagasawa *et al.*, 2020a
*SMU_1553c* comp.	UA159 *SMU_1553c* deletion mutant; *ermBP*, *SMU_437c*::P*_1554c_*-*SMU_1554c*-*1553c* *aph3*	This study
Δ*lytF* Δ*SMU_1553c*	UA159 *lytF* and *SMU_1553c* deletion mutant; *aad9*, *ermBP*	This study
WT P*_lytF_* reporter	UA159 *SMU_437c*::P*_lytF_*-*mScarlet-I* *aph3*	Nagasawa *et al.*, 2020b
Δ*SMU_1553c* P*_lytF_* reporter	UA159 *SMU_1553c* deletion mutant; *ermBP*, *SMU_437c*:: P*_lytF_*-*mScarlet-I* *aph3*	This study
*Bacillus subtilis*		
TAY3203	*trpC2* *lys1* Δ*aprE3* *nprE18* *nprR2* Δ*ydiR*-*ydjA* Δ*yqpP*-*yodU* (ΔSPβ) *amyE*::*nonA*-*spoVG* 3′ UTR *aph3*	Yamamoto *et al.*, 2014
Plasmid		
pJIR418	*ermBP*	Sloan *et al.*, 1992
pDL278	*aad9*	LeBlanc *et al.*, 1992

^a^ erm^S^: erythromycin susceptibility, kan^S^: kanamycin susceptibility, spec^S^: spectinomycin susceptibility

**Table 2.** Accumulated mRNAs in dead cells

Locus_tag	Gene	Product	Fold change (Dead/Live)
*SMU_1294*	*flaW*	flavodoxin	174.25
*SMU_370*		ABC transporter ATP-binding protein	149.18
*SMU_283*		hypothetical protein	25.81
*SMU_175*		hypothetical protein	25.5
*SMU_33*		hypothetical protein	25.27
*SMU_313*		PTS system sorbitol-specific transporter subunit IIA	15.74
*SMU_207c*		transposon protein	15.6
*SMU_677*		MerR family transcriptional regulator	15.12
*SMU_1147c*		hypothetical protein	14.72
*SMU_1496*	*lacA*	galactose-6-phosphate isomerase subunit LacA	11.78
*SMU_210c*		hypothetical protein	11.36
*SMU_812*		hypothetical protein	9.2
*SMU_134*		TetR/AcrR family transcriptional regulator	8.44
*SMU_574c*	*lrgB*	hypothetical protein	7.96
*SMU_642*		hypothetical protein	7.92
*SMU_1492*	*lacF*	PTS system lactose-specific transporter subunit IIA	7.79
*SMU_202c*		hypothetical protein	6.57
*SMU_21*	*mreD*	cell shape-determining protein MreD	6.45
*SMU_504*	*dam*	site-specific DNA-methyltransferase	6.13
*SMU_981*	*bglB1*	BglB fragment	6.06
*SMU_209c*		hypothetical protein	5.76
*SMU_41*		hypothetical protein	5.39
*SMU_1423*	*pdhA*	pyruvate dehydrogenase, TPP-dependent E1 component alpha-subunit	5.29
*SMU_791c*		hypothetical protein	4.98
*SMU_1895c*		hypothetical protein	4.98
*SMU_1790c*		transcriptional regulator	4.97
*SMU_1253c*		hypothetical protein	4.91
*SMU_2121c*		hypothetical protein	4.91
*SMU_115*		PTS system fructose-specific transporter subunit IIA	4.81
*SMU_373*		hypothetical protein	4.66
*SMU_199c*		hypothetical protein	4.52
*SMU_1261c*		phosphoribosyl-ATP pyrophosphohydrolase	4.48
*SMU_208c*		transposon protein	4.42
*SMU_1490*	*lacG*	6-phospho-beta-galactosidase	4.31
*SMU_1997*	*sigX*	ComX, transcriptional regulator of competence-specific genes	4.22
*SMU_1631*		peptidyl-prolyl cis-trans isomerase	4.19
*SMU_39*		hypothetical protein	4.15
*SMU_1494*	*lacC*	tagatose-6-phosphate kinase	4.12
*SMU_28*		ATP-binding protein	*
*SMU_55*		hypothetical protein	*
*SMU_56*		hypothetical protein	*
*SMU_92c*		transposase fragment	*
*SMU_94c*		transposase fragment	*
*SMU_136c*		transcriptional regulator	*
*SMU_194c*		hypothetical protein	*
*SMU_195c*		hypothetical protein	*
*SMU_206c*		hypothetical protein	*
*SMU_211c*		hypothetical protein	*
*SMU_212c*		hypothetical protein	*
*SMU_213c*		hypothetical protein	*
*SMU_214c*		hypothetical protein	*
*SMU_215c*		hypothetical protein	*
*SMU_216c*		hypothetical protein	*
*SMU_217c*		hypothetical protein	*
*SMU_378*		hypothetical protein	*
*SMU_379*		hypothetical protein	*
*SMU_594*		hypothetical protein	*
*SMU_605*		hypothetical protein	*
*SMU_1026*		hypothetical protein	*
*SMU_1027*		transcriptional regulator	*
*SMU_1030*		polyribonucleotide nucleotidyltransferase	*
*SMU_1031*	*xis*	transposon excisionase; Tn916 ORF1-like	*
*SMU_1259*		restriction endonuclease	*
*SMU_1369*		hypothetical protein	*
*SMU_1395c*		hypothetical protein	*
*SMU_1553c*		hypothetical protein	*
*SMU_1554c*		hypothetical protein	*
*SMU_1600*	*ptcB*	PTS system cellobiose transporter subunit IIB	*
*SMU_1701c*		hypothetical protein	*
*SMU_1804c*		hypothetical protein	*

The mRNAs detected in dead cells only and mRNAs that accumulated to levels that were more than 4-fold higher in dead cells than in live cells are shown in this table. All data from RNA-seq are shown in Table S4. * mRNAs detected in dead cells only.
