# Supplementary material for: Identification of a Novel Gene Involved in Cell-to-cell Communication-induced Cell Death and eDNA Production in Streptococcus mutans
Source: Microbes Environ. 2023 Jun 10;38(2):ME22085. doi: 10.1264/jsme2.ME22085 (PMC10308232; doi:10.1264/jsme2.ME22085)
Supplement: Supplementary file 1 — Supplementary Material [file 38_22085_s1.pdf]

Table S1 Primers for RT-PCR

Primer	Sequence
1553c Fw	gagatcattgaaattactgctcatttttatcg
1553c Rv	cataagtcattttctttcctccctgc
ldh Fw	ggtgctgtaggttcatcttacg
ldh Rv	gcaagtgcatgactaagatcaag
lytF Fw	gaatgctggctgttagttcttatac
lytF Rv	ctgtacagcgttgacatcaaaag

Table S2 Primers for mutant construction

Primer	Sequence
283 up Fw	GAAATCAGTTGTATGACTTCAATTGGTAC
283 up Rv fuse to ermBP	CTTTTACGTTTCCGGGTACAATTCGCCATTGTGTCCATCACCTATC
283 down Fw fuse to ermBP	GAAATAATTCTATGAGTCGCGCAGGTGCTGCAGTTATTTATTATG
283 down Rv	GATAATCTCTAAGAGAATGTTAACGGCTATG
1553c up Fw	CATTGTAACAGGGTTATGGACTATGTC
1553c up Rv fuse to ermBP	CTTTTACGTTTCCGGGTACAATTCGAGCAGTAATTTCAATGATCTCATGATC
1553c up Rv fuse to km	CAAGCTATAAGGTTATTGTCCTGGGGAGCAGTAATTTCAATGATCTCATGATC
1553c down Fw fuse to ermBP	GAAATAATTCTATGAGTCGCCAGGGAGGAAAGAAAATGACTTATG
1553c down Fw fuse to km	CTAGATTTAGATGTCTAAAAAGCAGGGAGGAAAGAAAATGACTTATGAAG
1553c down Rv	GAACTAGCTGAGCATAAGTAATAGATAAGG
1895c up Fw	CATCTTTGACGATATGGACGATAAAAG
1895c up Rv fuse to ermBP	CTTTTACGTTTCCGGGTACAATTCCCATTGTTTCAAATTGATTTAATTTTTGTGTATTC
1895c up Rv fuse to spec	GCCAGTCACGTTACGTTATTAGGTAGCTAACGTCTCTGTATCCATTG
1895c down Fw fuse to ermBP	GAAATAATTCTATGAGTCGCCCAAATCCTGTTATTGTTCCTTTTAGAG
1895c down Fw fuse to spec	CCGATTAAGATACTGCCTACCCTGTTATTGTTCCTTTTAGAGTACG
1895c down Rv	CATCTGTGTAGCACTTTTCCATTG
1554c up Fw	GAAATGGATCTGTCCAGTATAGCAG
1554c up Rv fuse to ermBP	CTTTTACGTTTCCGGGTACAATTCGTCCCTCTTCGTTTTCTTTATGATATG
lytF up Fw	GTGTAGAAGAAGAGGGTTATTATCATG
lytF up Rv fuse to spec	GCCAGTCACGTTACGTTATTAGCTCAATCGAAATCTCCTTTATTCTTTTTTAC
lytF down Fw fuse to spec	GATAAAATCCGATTAAGATACTGCCTACTTATATTTATCCTGACTAAGAGAACAAAAC
lytF down Rv	CTGAAAAAACAGAGCAAGCTAAC
ermBP Fw	GAATTGTACCCGGAAACGTAAAAG
ermBP Rv	GCGACTCATAGAATTATTTCCTCC
km Fw	CCCAGGACAATAACCTTATAGCTTG
km Rv	GCTTTTTAGACATCTAAATCTAGGTACTAAAAC
spec Fw	CTAATAACGTAACGTGACTGGCAAG
spec Rv	GTAGGCAGTATCTTAATCGGATTTTATCG

Table S3 Primers for construction of complemented strain

Primer	Sequence
437 up Fw	GTGAAGCTGTTGTTCCAGATTTG
437 up Rv fuse to P1554	CCTCTTCGAGGCTTGCGAGAGGCAAAAAAAGCCCG
P1554-3 Fw fuse to 437 up	CTTATGCGGGCTTTTTTTGCCTCTCGCAAGCCTCGAAGAGGTAATATTATTACG
P1554-3 Rv fuse to 437 down	GTATTTTTATGGATTTTTATTGTTTATCATAAGTCATTTTCTTTCCTCCCTG
Tldh Km 437 down Rv fuse 1553c	CGCAGGGAGGAAAGAAAATGACTTATGATAAACAATAAAAATCCATAAAAATACCAAGCATTG
437 down Rv	GGTATAGATGAAGTTTGCGGTC

Table S4 RNA-seq results of sorted live and dead cells

Locus_tag	Gene	Product	Fold change (Dead/Live)	Differential expression p-value	UA159_dead - Normalized TPM	UA159_live - Normalized TPM
SMU_01	dnaA	chromosome replication initiator DnaA	1.67	0.000	96.72	57.79
SMU_02	dnaN	DNA polymerase III subunit beta	0.69	0.001	89.74	130.14
SMU_05		hypothetical protein	1.26	0.009	175.77	139.32
SMU_06		GTP-binding protein YchF	0.68	0.000	146.51	215.71
SMU_07	pth	peptidyl-tRNA hydrolase	2.37	0.000	41.96	17.72
SMU_08	trcF	transcription-repair coupling factor	0.94	1.000	41.1	43.84
SMU_09		hypothetical protein	0.71	0.001	111.73	157.91
SMU_10		hypothetical protein	0.82	0.190	99.18	120.48
SMU_11		hypothetical protein	2.57	0.000	73.1	28.45
SMU_12		hypothetical protein	0.88	0.870	73.57	83.26
SMU_13		cell-cycle protein	1.84	0.000	60.92	33.11
SMU_14	hprT	hypoxanthine-guanine phosphoribosyltransferase	1.07	0.072	794.08	740.49
SMU_15	ftsH	cell division protein FtsH	1.07	0.006	2228.06	2087.29
SMU_16		amino acid permease	1.20	0.140	95.89	80.19
SMU_18		hypothetical protein	0.92	0.990	85.5	92.79
SMU_20	mreC	cell shape-determining protein MreC	1.05	0.990	21.78	20.72
SMU_21	mreD	cell shape-determining protein MreD	6.45	0.000	28.98	4.49
SMU_22	gbpB	secreted antigen GbpB/SagA	0.62	0.000	4593.78	7402.57
SMU_23	prs	ribose-phosphate pyrophosphokinase	0.79	0.002	234.9	297.77
SMU_24		aromatic amino acid aminotransferase	1.09	0.470	111.61	102.11
SMU_25	recO	DNA repair protein RecO	0.43	0.000	20.29	47.05
SMU_26	plsX	phosphate acyltransferase	1.05	0.470	345.55	330.14
SMU_27	acpP	acyl carrier protein	0.47	0.000	35.94	76.57
SMU_28		ATP-binding protein		0.000	13.32	0
SMU_29		phosphoribosylaminoimidazolesuccinocarboxamide synthase	1.57	0.012	49.55	31.55
SMU_30	purL	phosphoribosylformylglycinamidine synthase	2.13	0.000	101.15	47.58
SMU_31		hypothetical protein	2.36	0.000	38.32	16.27
SMU_32	purF	amidophosphoribosyltransferase	3.21	0.000	168.09	52.37
SMU_33		hypothetical protein	25.27	0.000	51.05	2.02
SMU_34	purM	phosphoribosylaminoimidazole synthetase	2.71	0.000	155.68	57.44
SMU_35	purN	phosphoribosylglycinamide formyltransferase	1.21	0.024	189.22	156.12
SMU_36		hypothetical protein	1.91	0.000	160.38	83.81
SMU_37	purH	bifunctional phosphoribosylaminoimidazolecarboxamide formyltransferas	2.22	0.000	229.78	103.51
SMU_38c		transcriptional regulator	2.01	0.005	27.45	13.63
SMU_39		hypothetical protein	4.15	0.000	32.47	7.82
SMU_40		hypothetical protein	2.36	0.000	97.24	41.27
SMU_41		hypothetical protein	5.39	0.000	97.76	18.13
SMU_42		hypothetical protein	1.34	0.026	79.12	58.92
SMU_43		site-specific DNA-methyltransferase restriction-modification protein	1.66	0.063	25.22	15.15
SMU_44		DNA mismatch repair protein	1.50	0.023	51.23	34.17
SMU_45		hypothetical protein	1.99	0.028	19.27	9.68
SMU_46		hypothetical protein	0.78	0.390	20.97	26.85
SMU_47		hypothetical protein	1.48	0.180	20.34	13.78
SMU_48	purD	phosphoribosylamine--glycine ligase	1.30	0.002	202.76	155.94
SMU_49		hypothetical protein	2.96	0.000	105.87	35.75
SMU_50	purE	5-(carboxyamino)imidazole ribonucleotide mutase	1.74	0.000	182.51	104.74
SMU_51	purK	5-(carboxyamino)imidazole ribonucleotide synthase	2.76	0.000	273.52	99.02
SMU_52		hypothetical protein	3.54	0.000	61.95	17.52
SMU_53		hypothetical protein	1.52	0.000	147.41	96.83
SMU_54		amino acid recemase	2.74	0.000	154.69	56.46
SMU_55		hypothetical protein		0.000	18.62	0
SMU_56		hypothetical protein		0.000	8.24	0
SMU_58		hypothetical protein	3.70	0.000	39	10.54
SMU_59	purB	adenylosuccinate lyase	1.89	0.000	215.92	114.46
SMU_60		DNA alkylation repair protein	0.91	0.810	113.13	124.47
SMU_61		transcriptional regulator	0.69	0.000	171.13	247.86
SMU_63c		hypothetical protein	0.75	0.000	4726.48	6307.17
SMU_64	ruvB	Holliday junction DNA helicase RuvB	1.11	0.000	2373.34	2139.02
SMU_65		protein tyrosine-phosphatase	1.25	0.000	2366.73	1895.74
SMU_66		hypothetical protein	1.00	1.000	1018.39	1021.16
SMU_67		acyltransferase	1.04	0.140	1547.92	1486.69
SMU_68		hypothetical protein	1.14	0.015	444.76	390.37
SMU_70	thrC	threonine synthase	0.83	0.094	191	229.95
SMU_71		cation efflux pump (multidrug resistance protein)	0.78	0.140	46.56	59.44
SMU_73		hypothetical protein	1.51	0.000	332.71	220.87
SMU_74		hypothetical protein	0.83	0.004	623.18	748.23
SMU_75		D-alanyl-D-alanine carboxypeptidase	0.80	0.000	448.28	559.83
SMU_76		N-acetyl-muramidase	0.82	0.019	298.07	362
SMU_78	fruA	exo-beta-D-fructosidase	1.95	0.000	81.94	42.09
SMU_79	fruB	exo-beta-D-fructosidase	1.92	0.011	25.95	13.5
SMU_80	hrcA	heat-inducible transcription repressor	1.18	0.000	1354.92	1150.76
SMU_81	grpE	heat shock protein GrpE	2.54	0.000	1027.48	404.44
SMU_82	dnaK	molecular chaperone DnaK	2.35	0.000	2396.19	1020.65
SMU_83	dnaJ	molecular chaperone DnaJ	2.12	0.000	1423.52	672.76
SMU_84	truA	tRNA pseudouridine synthase A	1.48	0.000	305.77	206.41
SMU_85	thiD	phosphomethylpyrimidine kinase	1.26	0.004	212.97	168.77
SMU_86		hypothetical protein	1.17	0.130	127.13	108.32
SMU_87		hypothetical protein	1.62	0.000	345.42	213.17
SMU_88c		mechanosensitive ion channel protein MscS	1.50	0.000	173.42	115.41
SMU_89c		nitrite transporter	1.29	0.270	32.68	25.29
SMU_91	ropA	trigger factor	0.83	0.003	708.2	848.37
SMU_92c		transposase fragment		0.000	8.26	0
SMU_93c		transposase fragment	0.09	0.000	8.51	92.29
SMU_94c		transposase fragment		0.000	17.71	0
SMU_97	pyrG	CTP synthetase	1.03	0.580	628.46	612.93
SMU_99	fbaA	fructose-bisphosphate aldolase	0.75	0.000	3072.43	4099.7
SMU_100		PTS system sorbose transporter subunit IIB	0.58	0.006	29.34	50.9

SMU_101		PTS system sorbose transporter subunit IIC	1.92	0.001	44.14	23
SMU_102		PTS system transporter subunit IID	0.91	0.870	93.43	102.47
SMU_103		PTS system transporter subunit IIA	1.24	0.350	36.88	29.69
SMU_104		alpha-glucosidase	1.21	0.150	81.39	67.18
SMU_105		transcriptional regulator; repressor of sugar transport operon	1.08	0.500	111.94	103.54
SMU_106c		transposase fragment	0.78	0.000	323.79	413.04
SMU_107		hypothetical protein	1.07	1.000	6.21	5.82
SMU_108		hypothetical protein	0.79	0.430	23.66	29.9
SMU_109		permease	0.51	0.000	58.69	114.75
SMU_110	mutR	transcriptional regulator MutR	0.63	0.075	18.69	29.74
SMU_112c		transcriptional regulator	2.83	0.000	23.6	8.35
SMU_113		1-phosphofructokinase	3.12	0.001	15.22	4.88
SMU_114		PTS system fructose-specific transporter subunitIIBC	1.52	0.150	22.78	15.03
SMU_115		PTS system fructose-specific transporter subunitIIA	4.81	0.000	16.58	3.45
SMU_116	lacD2	tagatose 1,6-diphosphate aldolase	3.41	0.000	18.1	5.31
SMU_117c		hypothetical protein	1.13	0.820	11.34	10.07
SMU_118c		esterase	1.14	0.037	326.58	286.59
SMU_119	adh	alcohol dehydrogenase	1.91	0.000	144.17	75.53
SMU_120		50S ribosomal protein L28	0.32	0.000	146.4	456.21
SMU_121	dinF	damage-inducible protein DinF	1.29	0.019	123.64	95.92
SMU_123		DNA polymerase III PolC	1.29	0.000	455.38	353.02
SMU_124		MarR family transcriptional regulator	0.96	0.800	858.28	890.05
SMU_125		hypothetical protein	1.51	0.000	915.09	607.33
SMU_127	adhA	acetoin dehydrogenase TPP-dependent, E1 component subunit alpha	0.56	0.000	510.97	905.68
SMU_128	adhB	acetoin dehydrogenase TPP-dependent, E1 component subunit beta	1.08	0.140	414.62	382.92
SMU_129	adhC	branched-chain alpha-keto acid dehydrogenase E2 subunit	1.04	0.480	408.29	391.9
SMU_130	adhD	dihydrolipoamide dehydrogenase	1.19	0.000	555.33	465.55
SMU_131	lplA	lipoate-protein ligase	0.97	0.890	410.09	424.26
SMU_132		hippurate amidohydrolase	1.06	0.480	210.22	197.72
SMU_133c		MDR permease	2.36	0.001	26.47	11.22
SMU_134		TetR/AcrR family transcriptional regulator	8.44	0.000	14.68	1.74
SMU_135	mleR	transcriptional regulator	1.04	0.860	68.77	66.2
SMU_136c		transcriptional regulator		0.000	16.56	0
SMU_137	mleS	malate dehydrogenase	1.12	0.049	387.34	346.28
SMU_138		malate permease	1.46	0.000	315.03	216.2
SMU_139		hypothetical protein	1.07	0.280	298.55	278.07
SMU_140		glutathione reductase	1.07	0.260	363.7	340.44
SMU_141		hypothetical protein	0.86	0.300	268.8	311.52
SMU_143c		peptide deformylase	0.94	0.700	432.75	462.7
SMU_144c		transcriptional regulator	0.48	0.000	171.2	353.51
SMU_145		hypothetical protein	0.61	0.000	212.43	350.36
SMU_148	adhE	bifunctional acetaldehyde-CoA/alcohol dehydrogenase	1.08	0.360	205.52	190.77
SMU_149		transposase	1.21	0.490	29.47	24.42
SMU_150		hypothetical protein	1.22	0.000	1018.11	831.37
SMU_151		hypothetical protein	0.80	0.000	5868.91	7356.6
SMU_152		hypothetical protein	0.61	0.000	1248.52	2057.66
SMU_153		hypothetical protein	0.89	0.006	5246.63	5920.01
SMU_154		30S ribosomal protein S15	0.70	0.000	3426.44	4875.5
SMU_155	pnpA	polynucleotide phosphorylase	1.33	0.000	364.75	273.92
SMU_156		hypothetical protein	0.99	1.000	175.3	176.85
SMU_157	cysE	serine acetyltransferase	1.26	0.000	567.65	452.15
SMU_158	cysS	cysteinyl-tRNA synthetase	1.20	0.029	199.67	166.53
SMU_159		hypothetical protein	1.18	0.012	304.41	258.14
SMU_160		metallopeptidase	1.38	0.000	292.5	211.85
SMU_161		transcriptional regulator	1.89	0.000	234.71	124.15
SMU_162c		hypothetical protein	1.43	0.001	125.46	87.54
SMU_163c		hypothetical protein	1.36	0.000	192.84	142.02
SMU_164		23S rRNA (guanosine(2251)-2'-O)-methyltransferase RlmB	1.00	1.000	164.73	165.52
SMU_165		hypothetical protein	0.97	0.950	272.7	280.1
SMU_166		hypothetical protein	0.69	0.000	156.04	225.55
SMU_167		hypothetical protein	0.52	0.000	281.31	536.98
SMU_168		transcriptional regulator	0.64	0.000	663	1036.83
SMU_169		50S ribosomal protein L13	0.85	0.000	5314.97	6254
SMU_170		30S ribosomal protein S9	0.84	0.000	4330.06	5169.91
SMU_172		cell growth regulatory protein	0.78	0.003	187.07	239.45
SMU_173		ppGpp-regulated growth inhibitor	0.62	0.000	211.19	339.25
SMU_174c		hypothetical protein	1.23	0.140	72.2	58.57
SMU_175		hypothetical protein	25.50	0.000	56.1	2.2
SMU_176		hypothetical protein	3.77	0.000	89.5	23.71
SMU_177		hypothetical protein	1.00	1.000	70.42	70.15
SMU_178		hypothetical protein	1.07	0.610	100.21	93.52
SMU_179		hypothetical protein	1.55	0.002	78.93	50.97
SMU_180		oxidoreductase	1.98	0.000	169.04	85.18
SMU_181		mevalonate kinase	0.83	0.110	159.1	191.99
SMU_182	sloA	iron ABC transporter ATP-binding protein	0.59	0.000	151.97	258.59
SMU_183	sloB	Mn/Zn ABC transporter	0.77	0.001	197.5	256.1
SMU_184	sloC	ABC transporter metal binding lipoprotein	0.88	0.610	189.55	216.13
SMU_185		hypothetical protein	0.97	0.970	264.21	273.22
SMU_186	sloR	metal-dependent transcriptional regulator	0.99	1.000	363.02	365.49
SMU_187c		hypothetical protein	1.02	0.820	449.22	442.41
SMU_188c		heat shock protein 33	0.64	0.000	326.84	508.58
SMU_189		hypothetical protein	1.75	0.000	76.77	43.99
SMU_191c		integrase	2.36	0.014	16.7	7.09
SMU_193c		hypothetical protein	0.94	1.000	12.9	13.77
SMU_194c		hypothetical protein		0.000	12.96	0
SMU_195c		hypothetical protein		0.000	4.98	0
SMU_196c		transfer protein	1.10	0.540	81.71	74.25
SMU_197c		hypothetical protein	2.24	0.000	34.55	15.42
SMU_198c		conjugative transposon protein	1.90	0.031	20.01	10.51
SMU_199c		hypothetical protein	4.52	0.000	47.28	10.46
SMU_200c		hypothetical protein	1.55	0.020	45.37	29.33

SMU_201c		transposon protein	1.69	0.040	28.71	16.97
SMU_202c		hypothetical protein	6.57	0.000	39.46	6.01
SMU_204c		hypothetical protein	1.02	1.000	31.4	30.88
SMU_205c		hypothetical protein	1.67	0.094	20.89	12.51
SMU_206c		hypothetical protein		0.005	3.62	0
SMU_207c		transposon protein	15.60	0.000	21.53	1.38
SMU_208c		transposon protein	4.42	0.000	36.79	8.33
SMU_209c		hypothetical protein	5.76	0.000	44.27	7.68
SMU_210c		hypothetical protein	11.36	0.000	20.68	1.82
SMU_211c		hypothetical protein		0.002	3.94	0
SMU_212c		hypothetical protein		0.570	1.34	0
SMU_213c		hypothetical protein		0.000	6.45	0
SMU_214c		hypothetical protein		0.002	4.01	0
SMU_215c		hypothetical protein		0.001	4.3	0
SMU_216c		hypothetical protein		0.580	1.37	0
SMU_217c		hypothetical protein		0.000	4.7	0
SMU_218		transcriptional regulator	0.50	0.000	40.11	80.39
SMU_219		hypothetical protein	0.67	0.020	37.69	56.65
SMU_220c		hypothetical protein	0.84	0.150	168.01	201.02
SMU_221c		integrase	0.92	0.760	229.01	249.64
SMU_222c		integrase fragment	1.09	0.370	155.72	143.01
SMU_223c		hypothetical protein	0.63	0.000	154.44	245.51
SMU_224c		hypothetical protein	0.96	0.940	121.71	127.1
SMU_225c		hypothetical protein	1.45	0.000	133.86	92.36
SMU_226c		transposase	1.06	0.320	366.17	345.73
SMU_227c		hypothetical protein	0.67	0.000	191.85	287.59
SMU_228		alkaline-shock protein-like protein	0.68	0.000	220.78	324.21
SMU_229		hypothetical protein	1.02	0.750	404.33	396.99
SMU_231	ilvB	acetolactate synthase catalytic subunit	0.82	0.001	610.6	741.38
SMU_232	ilvH	acetolactate synthase small subunit	1.04	0.320	826.1	795.65
SMU_233	ilvC	ketol-acid reductoisomerase	0.74	0.000	1147.27	1554.66
SMU_234	ilvA	threonine dehydratase	0.78	0.000	400.01	513.05
SMU_235		hypothetical protein	0.70	0.000	714.11	1021.77
SMU_236c		transcriptional regulator	0.55	0.000	73.49	133.64
SMU_237c		hypothetical protein	0.82	0.360	48.97	59.4
SMU_238c		ABC transporter ATP-binding protein	1.63	0.002	64.54	39.52
SMU_239c		hypothetical protein	3.36	0.014	9.72	2.89
SMU_241c		amino acid ABC transporter ATP-binding protein	1.46	0.000	218.82	149.77
SMU_242c		amino acid ABC transporter permease	1.50	0.000	149.47	99.8
SMU_243		hypothetical protein	1.89	0.000	117.69	62.13
SMU_244	bacA	undecaprenyl pyrophosphate phosphatase	1.45	0.002	106.95	73.66
SMU_245	mecA	adaptor protein MecA	0.88	0.280	719.33	817.48
SMU_246	rgpG	glycosyl transferase N-acetylglucosaminyltransferase RgpG	0.83	0.008	505.44	607.11
SMU_247		ABC transporter ATP-binding protein	1.19	0.000	853.86	718.23
SMU_248		ABC transporter membrane protein	1.40	0.000	774.97	553.24
SMU_249	nifS	NifS protein-like protein class-V aminotransferase	1.19	0.000	716.79	601.17
SMU_250	nifU	nitrogen fixation-like protein, NifU	1.46	0.000	706.71	484.41
SMU_251		ABC transporter membrane protein	1.26	0.000	624	495.06
SMU_252		hypothetical protein	0.70	0.015	56.56	81.25
SMU_253	dacA	D-alanyl-D-alanine carboxypeptidase	0.86	0.450	101.64	118.63
SMU_255	oppA	oligopeptide ABC transporter substrate-binding protein OppA	1.08	0.140	496.45	460.82
SMU_256	oppB	oligopeptide transport system permease OppB	1.57	0.000	311.91	198.57
SMU_257	oppC	transmembrane permease OppC	1.60	0.000	219.76	137.73
SMU_258	oppD	oligopeptide ABC transporter ATP-binding proteinOppD	1.29	0.000	491.74	381.71
SMU_259	oppF	oligopeptide ABC transporter ATP-binding proteinOppF	0.94	0.760	482.8	512.49
SMU_260		hypothetical protein	0.75	0.000	258.63	346.85
SMU_261c		transcriptional regulator	1.23	0.630	11.31	9.22
SMU_262	otcA	putrescine carbamoyltransferase	0.95	1.000	36.98	38.78
SMU_263		amino acid antiporter	0.93	1.000	31.29	33.61
SMU_264		agmatine deiminase	0.97	1.000	45.54	46.92
SMU_265	arcC	amino acid kinase	1.27	0.160	52.24	41.15
SMU_267c		bifunctional glutamate--cysteine ligase/glutathione synthetase	1.18	0.044	195.74	165.96
SMU_268	purA	adenylosuccinate synthetase	0.90	0.470	508.36	567.58
SMU_270	sgaT	PTS system ascorbate-specific transporter subunit IIC	1.50	0.082	34.16	22.83
SMU_271	ptxB	PTS system transporter subunit IIB	0.35	0.000	106.98	301.71
SMU_272	ptxA	PTS system transporter subunit IIA	1.29	0.001	217.34	168.04
SMU_273		3-keto-L-gulonate-6-phosphate decarboxylase	1.09	0.390	142.8	130.53
SMU_274		L-xylulose 5-phosphate 3-epimerase	1.72	0.000	151.11	87.77
SMU_275		L-ribulose-5-phosphate 4-epimerase	1.43	0.000	178.22	124.47
SMU_276c		hypothetical protein		1.000	0	0
SMU_277		hypothetical protein	0.64	0.000	318.25	495.73
SMU_278		hypothetical protein	0.65	0.000	1661.19	2539.51
SMU_279		hypothetical protein	0.93	1.000	20.23	21.68
SMU_281		hypothetical protein	3.12	0.000	38.2	12.24
SMU_283		hypothetical protein	25.81	0.000	100.67	3.9
SMU_284		hypothetical protein	1.85	0.000	68.5	36.96
SMU_285		hypothetical protein	1.37	0.003	128	93.24
SMU_286		ABC transporter ATP-binding protein ComA	1.18	0.013	307.08	261.13
SMU_287		ComB, accessory factor for ComA	0.95	0.950	176.52	185.3
SMU_289		transcriptional regulator	1.14	0.620	36.17	31.73
SMU_290		L-ascorbate 6-phosphate lactonase	1.26	0.097	76.68	60.72
SMU_291	tkt	transketolase	0.94	0.700	501.29	533.28
SMU_292		transcriptional regulator	1.33	0.370	20.45	15.33
SMU_293		hypothetical protein	2.89	0.000	28.83	9.99
SMU_294		hypothetical protein	0.72	0.039	47.79	66.75
SMU_295		hypothetical protein	0.74	0.010	88.04	118.94
SMU_296		hypothetical protein	0.74	0.001	128.81	174.97
SMU_297	polI	DNA polymerase I	0.95	0.800	361.64	380.6
SMU_298		hypothetical protein	0.91	0.540	571.42	628.15
SMU_299c		bacteriocin peptide	0.68	0.003	67.71	100.06
SMU_300	tgt	queuine tRNA-ribosyltransferase	1.29	0.000	258.81	199.91

SMU_301		hypothetical protein	0.79	0.002	221.42	281.53
SMU_302		hypothetical protein	1.68	0.000	82.69	49.25
SMU_303		hypothetical protein	1.19	0.002	412.43	345.73
SMU_304		deaminase	1.01	0.880	502.89	498.13
SMU_305		hypothetical protein	0.98	1.000	272.64	278.51
SMU_307	pgi	glucose-6-phosphate isomerase	0.87	0.052	1099.97	1269.35
SMU_308		sorbitol-6-phosphate 2-dehydrogenase	1.89	0.001	41.05	21.67
SMU_309		regulator of sorbitol operon	0.76	0.380	18.68	24.47
SMU_310		sorbitol operon activator	3.24	0.000	31.13	9.61
SMU_311		PTS system sorbitol (glucitol) transporter subunit IIC2	1.32	0.094	57.62	43.5
SMU_312		PTS system sorbitol phosphotransferase transporter subunit IIBC	1.15	0.480	48.77	42.5
SMU_313		PTS system sorbitol-specific transporter subunitIIA	15.74	0.000	24.4	1.55
SMU_314		hypothetical protein	1.35	0.017	90.08	66.75
SMU_317		2,3,4,5-tetrahydropyridine-2,6-dicarboxylate N-acetyltransferase	1.03	0.600	508.79	495.84
SMU_318		hippurate hydrolase	1.09	0.094	453.52	415.15
SMU_320		5-formyltetrahydrofolate cyclo-ligase	0.62	0.000	193.59	311.05
SMU_321		hypothetical protein	0.53	0.000	172.67	324.15
SMU_322c		glucose-1-phosphate uridylyltransferase	0.85	0.019	736.28	866.36
SMU_323	gpsA	NAD(P)H-dependent glycerol-3-phosphate dehydrogenase	0.75	0.000	797	1063.91
SMU_325		deoxyuridine 5'-triphosphate nucleotidohydrolase	1.18	0.033	214.88	181.44
SMU_326		hypothetical protein	1.10	0.089	399.09	362.81
SMU_327		DNA repair protein RadA	1.16	0.000	780.83	672.82
SMU_328		carbonic anhydrase	0.95	0.550	1421.82	1498.92
SMU_329		hypothetical protein	0.99	0.940	1750.35	1763.97
SMU_330	gltX	glutamyl-tRNA synthetase	1.34	0.000	273.09	204.15
SMU_331		transcriptional regulator	1.31	0.000	330.05	252.31
SMU_332		hypothetical protein	0.77	0.005	139.39	180.64
SMU_333		hypothetical protein	0.99	0.990	290.22	293.31
SMU_334		argininosuccinate synthase	1.07	0.630	107.39	100.82
SMU_335		argininosuccinate lyase	1.12	0.410	87.02	77.98
SMU_336	rnpA	ribonuclease P	0.45	0.000	115.1	257.69
SMU_337		hypothetical protein	0.93	0.780	317.16	340.36
SMU_338		RNA-binding protein	1.12	0.120	273.56	245.21
SMU_339		hypothetical protein	1.43	0.000	302.33	211.89
SMU_340		50S ribosomal protein L34	0.98	0.860	1838.13	1869.68
SMU_341		deoxyribonuclease	0.72	0.000	432.92	603.91
SMU_342		hypothetical protein	1.31	0.000	398.21	304.25
SMU_343		hypothetical protein	0.98	0.950	336.74	345.08
SMU_344		hypothetical protein	1.08	0.150	439.75	408.1
SMU_345c		hypothetical protein	1.60	0.000	181.17	112.92
SMU_346		NADH dehydrogenase; NAD(P)H nitroreductase	1.98	0.000	153.73	77.82
SMU_348		histidine triad (HIT) hydrolase	0.69	0.000	366.66	529.97
SMU_349		KsgA/Dim1 family 16S ribosomal RNA methyltransferase	0.86	0.078	653.72	760.14
SMU_350		hypothetical protein	0.69	0.000	328.69	479.54
SMU_351		GTPase RsgA	0.77	0.000	649.53	839.83
SMU_352		ribulose-phosphate 3-epimerase	0.69	0.000	2430.41	3504.21
SMU_353		hypothetical protein	0.74	0.000	1949.84	2620.85
SMU_354		hypothetical protein	0.85	0.001	1449.28	1710.06
SMU_355		CMP-binding factor	0.74	0.000	2851.44	3829.7
SMU_356	purR	pur operon repressor	1.30	0.000	618.64	474.62
SMU_357		30S ribosomal protein S12	0.89	0.017	4575.14	5122.81
SMU_358		30S ribosomal protein S7	0.90	0.008	7169.23	7933.9
SMU_359		elongation factor G	0.98	0.760	3601.14	3667.46
SMU_360	gapC	extracellular glyceraldehyde-3-phosphate dehydrogenase	0.68	0.000	5150.95	7605.73
SMU_361	pgk	phosphoglycerate kinase	1.02	0.520	950.32	929.53
SMU_362		hypothetical protein	2.04	0.000	62.6	30.7
SMU_363	glnR	transcriptional regulator; glutamine synthetase repressor	0.53	0.000	1607.5	3060.19
SMU_364	glnA	glutamate--ammonia ligase	0.72	0.000	862.14	1197.51
SMU_365	gltA	glutamate synthase	0.95	0.890	212.23	224.22
SMU_366	gltB	glutamate synthase	1.27	0.001	281.43	221.59
SMU_367		hypothetical protein	1.03	0.660	380.52	370.46
SMU_368c		hypothetical protein	1.04	0.410	588.84	566.92
SMU_369c		hypothetical protein	1.42	0.000	326.41	229.13
SMU_370		ABC transporter ATP-binding protein	149.18	0.000	50.72	0.34
SMU_371		hypothetical protein	1.01	1.000	22.97	22.71
SMU_372		hypothetical protein	2.00	0.087	11.74	5.87
SMU_373		hypothetical protein	4.66	0.000	12.45	2.67
SMU_374		oxidoreductase	1.53	0.210	17.73	11.61
SMU_375		hypothetical protein	1.10	0.880	18.67	16.9
SMU_376		aminotransferase	0.90	0.930	35.74	39.51
SMU_378		hypothetical protein		0.000	5.74	0
SMU_379		hypothetical protein		0.000	5.98	0
SMU_381c		hypothetical protein	0.78	0.000	426.07	546.75
SMU_382c		oxidoreductase	3.33	0.000	33.26	9.99
SMU_383c		reductase	1.19	0.470	31.97	26.91
SMU_384		hypothetical protein	1.89	0.000	97.86	51.76
SMU_385		glycoprotein endopeptidase	1.12	0.220	169.01	151.18
SMU_386		ribosomal-protein-alanine acetyltransferase	1.53	0.000	130.65	85.37
SMU_387		UGMP family protein	1.10	0.230	189.41	171.55
SMU_388		branched-chain amino acid ABC transporter permease	0.72	0.000	198.27	275.2
SMU_389		hypothetical protein	1.28	0.001	229.92	179.6
SMU_390		hypothetical protein	1.14	0.130	169.45	148.54
SMU_391c		hypothetical protein	0.38	0.000	1002.74	2654.18
SMU_392c		hypothetical protein	0.95	0.890	166.49	175.15
SMU_393		hypothetical protein	0.66	0.001	76.53	115.81
SMU_394c		hypothetical protein	0.53	0.000	91.78	172.64
SMU_395	pepX	x-prolyl-dipeptidyl aminopeptidase	1.04	0.810	76.14	72.99
SMU_396	glpF	glycerol uptake facilitator protein	0.70	0.000	1008.49	1445.05
SMU_399		hypothetical protein	1.12	0.066	344.95	308.18
SMU_400		secreted esterase	0.99	0.960	571.48	578.1
SMU_401c		hypothetical protein	0.99	0.990	356.93	362

SMU_402	pfl	pyruvate formate-lyase	1.11	0.002	1065.52	956.61
SMU_403		DNA polymerase IV	1.10	0.240	187.4	169.64
SMU_404c		hypothetical protein	0.85	0.210	253.78	296.86
SMU_405c		transcriptional regulator	0.57	0.000	74.76	131.47
SMU_406c		hypothetical protein	0.91	0.730	179.59	197.41
SMU_407		hypothetical protein	0.74	0.049	58.04	78.01
SMU_408		permease	1.14	0.020	399.44	349.48
SMU_409		hypothetical protein	0.77	0.000	791.17	1026.73
SMU_410	brpA	transcriptional regulator	1.22	0.003	294.46	241.22
SMU_411c		hypothetical protein	0.58	0.000	146.03	250.68
SMU_412c		cell-cycle regulation protein	0.86	0.190	406.49	470.88
SMU_413		ABC transporter ATP-binding protein	1.30	0.000	320.18	246.94
SMU_414		ABC transporter permease	1.34	0.009	116.84	87.43
SMU_415		hypothetical protein	0.69	0.000	317.44	456.82
SMU_416		tRNA (guanine-N(7)-)-methyltransferase	0.80	0.007	194.99	245.19
SMU_417		ribosome maturation protein RimP	0.68	0.000	228.52	338.06
SMU_418	nusA	transcription elongation factor NusA	0.89	0.280	936.53	1052.45
SMU_419		hypothetical protein	0.92	0.640	422.85	461.34
SMU_420		hypothetical protein	1.83	0.000	219.77	120.21
SMU_421		translation initiation factor IF-2	1.05	0.130	950.68	901.27
SMU_422		ribosome-binding factor A	0.74	0.000	287.77	387.52
SMU_423		hypothetical protein	0.65	0.000	3884.54	6011.53
SMU_424	copY	negative transcriptional regulator, CopY	1.07	0.260	382.74	358.27
SMU_426	copA	copper-transporting ATPase	1.15	0.001	703.07	610.81
SMU_427	copZ	copper chaperone	0.78	0.000	519.61	663.28
SMU_428		hypothetical protein	0.64	0.000	185.21	290.75
SMU_429c		hypothetical protein	1.27	0.340	30.06	23.71
SMU_431		ABC transporter ATP-binding protein	1.14	0.680	28.37	24.85
SMU_432		ABC transporter	2.03	0.003	30.97	15.26
SMU_433		transcriptional regulator	0.68	0.000	150.99	223.19
SMU_434		hypothetical protein	0.63	0.000	173.69	273.87
SMU_435		N-acetylglucosamine-6-phosphate deacetylase	0.46	0.000	161.87	353.35
SMU_436c		transposase, ISSmu1	1.15	0.630	25.89	22.44
SMU_438c		(R)-2-hydroxyglutaryl-CoA dehydratase activator-like protein	0.93	1.000	38.97	42
SMU_439		transcriptional regulator	1.11	0.630	41.45	37.2
SMU_440		hypothetical protein	1.23	0.026	154.4	125.22
SMU_441		transcriptional regulator	1.39	0.000	250.9	180.72
SMU_442		hypothetical protein	1.27	0.000	470.65	369.73
SMU_444		hypothetical protein	1.12	0.150	209.77	187.07
SMU_445	sygA	glycyl-tRNA synthetase subunit alpha	1.45	0.000	198.27	136.86
SMU_446	sygB	glycyl-tRNA synthetase subunit beta	1.33	0.000	239.42	180.16
SMU_447		hypothetical protein	2.29	0.000	329.62	143.95
SMU_448		hypothetical protein	3.84	0.000	60.18	15.69
SMU_449	proB	gamma-glutamyl kinase	0.71	0.000	177.12	249.61
SMU_450	proA	gamma-glutamyl phosphate reductase	1.02	0.740	333.1	325.4
SMU_451		hypothetical protein	0.29	0.000	25.48	86.72
SMU_453		16S rRNA m(4)C1402 methyltransferase	0.81	0.001	390.89	483.84
SMU_454	ftsL	cell division protein	1.24	0.036	129.29	104.07
SMU_455	pbp2x	penicillin-binding protein 2X	1.17	0.008	372.67	318.33
SMU_456	mraY	phospho-N-acetylmuramoyl-pentapeptide- transferase	1.02	0.850	233.43	229.18
SMU_457		hypothetical protein	2.26	0.000	80.53	35.57
SMU_458		ATP-dependent RNA helicase	0.72	0.000	156.05	217.45
SMU_459		ABC transporter amino acid binding protein	1.03	0.880	110.12	107.32
SMU_460		amino acid ABC transporter permease	1.43	0.000	192.62	134.85
SMU_461		amino acid ABC transporter ATP-binding protein	1.02	0.790	232.24	226.81
SMU_462		hypothetical protein	1.59	0.000	94.49	59.44
SMU_463	trxB	thioredoxin reductase	0.75	0.000	1224.31	1622.78
SMU_464		nicotinate phosphoribosyltransferase	0.84	0.089	242.52	289.72
SMU_465	nadE	NAD synthetase	1.46	0.000	371.27	254.04
SMU_466	pepC	cysteine aminopeptidase	0.98	0.900	559.55	572.54
SMU_467	pbp1a	penicillin-binding protein 1a; membrane carboxypeptidase	0.86	0.076	665.89	774.11
SMU_469	recU	Holliday junction-specific endonuclease	0.50	0.000	727.52	1460.66
SMU_470		hypothetical protein	1.12	0.520	56.36	50.16
SMU_471		hypothetical protein	0.69	0.000	536.73	782.34
SMU_472		N-6 adenine-specific DNA methylase	0.92	0.640	452.49	492.38
SMU_473		hypothetical protein	0.77	0.000	357.15	461.28
SMU_474	luxS	S-ribosylhomocysteinase	0.47	0.000	192.91	407.69
SMU_475		hypothetical protein	0.93	0.430	1155.05	1248.32
SMU_478	kguA	guanylate kinase	0.72	0.000	580.82	806.03
SMU_479	rpoZ	DNA-directed RNA polymerase subunit omega	0.92	0.720	329.57	357.05
SMU_480	priA	primosome assembly protein PriA	1.09	0.410	148.29	136.38
SMU_481		methionyl-tRNA formyltransferase	1.60	0.000	296.05	185.06
SMU_482	sunL	16S rRNA (cytosine(967)-C(5))-methyltransferase	1.15	0.056	245.05	213.53
SMU_483		phosphoprotein phosphatase	1.12	0.006	735.55	654.99
SMU_484	pknB	serine/threonine protein kinase	1.01	0.690	941.34	927.48
SMU_485		hypothetical protein	0.88	0.960	19.93	22.59
SMU_486		histidine kinase	1.31	0.100	56.18	42.76
SMU_487		response regulator	1.24	0.056	104.4	84.09
SMU_488		hydrolase	0.78	0.087	60.93	78.38
SMU_489		hypothetical protein	0.44	0.000	51.94	117.66
SMU_490	pflC	pyruvate formate-lyase activating enzyme	1.51	0.045	37.16	24.53
SMU_491		DeoR-type transcriptional regulator	1.36	0.084	50.33	36.95
SMU_493	pfl2	formate acetyltransferase	1.30	0.045	82.67	63.47
SMU_494		fructose-6-phosphate aldolase	1.05	0.840	53.29	50.61
SMU_495	gldA	glycerol dehydrogenase	1.16	0.240	95.02	82.12
SMU_496	cysK	cysteine synthetase A	1.40	0.000	491.63	351.78
SMU_497c		hypothetical protein	0.94	0.970	56.06	59.62
SMU_498	comF	late competence protein	0.82	0.000	2431.28	2981.24
SMU_499		late competence protein	0.79	0.000	910.85	1159.18
SMU_500		ribosome-associated protein	0.83	0.000	1553.2	1876.09
SMU_501		hypothetical protein	0.81	0.160	73.6	90.95

SMU_502		hypothetical protein	1.04	0.840	85.14	82.15
SMU_503c		hypothetical protein	1.58	0.001	74.96	47.43
SMU_504	dam	site-specific DNA-methyltransferase	6.13	0.000	12.2	1.99
SMU_505		adenine-specific DNA methylase	0.79	0.000	1633.48	2075.19
SMU_506		type II restriction endonuclease	0.95	0.620	914	966.72
SMU_507		DeoR family transcriptional regulator	0.99	1.000	234.36	237.9
SMU_508		hypothetical protein	0.97	0.910	342.5	353.37
SMU_509		hypothetical protein	1.32	0.028	91.83	69.74
SMU_510c		hypothetical protein	0.48	0.000	115.31	239.38
SMU_512c		hypothetical protein	1.42	0.006	93.67	66.15
SMU_513		hypothetical protein	0.81	0.070	124.22	153.23
SMU_514		transcriptional regulator	0.74	0.170	27.33	37.14
SMU_515		hypothetical protein	2.79	0.000	25.94	9.3
SMU_516		hypothetical protein	1.05	0.690	132.24	126.21
SMU_517		phosphopantetheine adenylyltransferase	1.14	0.140	170.99	150.23
SMU_518		hypothetical protein	1.12	0.120	245.39	219.5
SMU_520		hypothetical protein	2.17	0.000	96.1	44.33
SMU_521		hypothetical protein	1.50	0.000	262.83	174.94
SMU_522		23S rRNA (adenine(2503)-C(2))-methyltransferase RlmN	1.12	0.031	435.46	387.67
SMU_523		hypothetical protein	1.12	0.190	181.24	161.88
SMU_524		ABC transporter ATP-binding protein	2.05	0.000	83.85	40.89
SMU_525		ABC transporter ATP-binding protein	1.31	0.110	53.52	40.89
SMU_526c		transcriptional regulator	0.73	0.140	28.95	39.43
SMU_527		hypothetical protein	1.13	0.078	266.17	235.35
SMU_528c		hypothetical protein	0.69	0.000	157.33	229.57
SMU_529		hypothetical protein	0.68	0.000	104.54	153.51
SMU_530c		hypothetical protein	0.62	0.000	376.38	606.14
SMU_531		chorismate mutase	0.80	0.000	403.6	504.37
SMU_532	trpE	anthranilate synthase component I	1.15	0.008	461.52	401.57
SMU_533	trpG	anthranilate synthase component II	1.19	0.006	329.48	277
SMU_534	trpD	anthranilate phosphoribosyltransferase	0.98	0.960	399.55	406.8
SMU_535	trpC	indole-3-glycerol phosphate synthase	0.81	0.003	377.85	464.08
SMU_536	trpF	N-(5'-phosphoribosyl)anthranilate isomerase	1.10	0.098	370.78	336.88
SMU_537	trpB	tryptophan synthase subunit beta	0.97	0.910	517.57	531.44
SMU_538	trpA	tryptophan synthase subunit alpha	1.16	0.006	428.19	367.79
SMU_539c		signal peptidase type IV	0.80	0.028	138.53	173.68
SMU_540	dpr	peroxide resistance protein Dpr	0.41	0.000	3467.21	8389.74
SMU_541		hypothetical protein	1.76	0.001	57	32.31
SMU_542	glk	glucose kinase	1.29	0.000	374.32	289.54
SMU_543		hypothetical protein	0.68	0.000	357.72	523.16
SMU_545		hypothetical protein	1.02	0.960	55.37	54.49
SMU_546		GTP-binding protein	1.03	0.490	691.6	671.61
SMU_547		hypothetical protein	0.94	0.710	530.68	565.77
SMU_548	murD	UDP-N-acetylmuramoyl-L-alanyl-D-glutamate synthetase	1.12	0.090	288.06	256.75
SMU_549	murG	undecaprenyldiphospho-muramoylpentapeptide beta-N-acetylglucosamin	1.12	0.170	199.39	178.57
SMU_550	ftsQ	cell division protein FtsQ	1.02	0.930	104.87	103.2
SMU_551	ftsA	cell division protein FtsA	0.99	0.930	781.29	792.26
SMU_552	ftsZ	cell division protein FtsZ	1.06	0.037	1516.3	1429.35
SMU_553	ylmE	hypothetical protein	0.97	0.790	860.6	891.37
SMU_554	ylmF	hypothetical protein	1.12	0.001	1277	1144.18
SMU_555	ylmG	hypothetical protein	0.66	0.000	233.13	352.7
SMU_556	ylmH	hypothetical protein	0.79	0.000	346.29	441.03
SMU_557	divIVA	cell division protein DivIVA	0.62	0.000	670.49	1082.8
SMU_558		isoleucyl-tRNA synthetase	0.89	0.610	259	292.2
SMU_560c		hypothetical protein	0.53	0.000	192.81	367.24
SMU_561c		hydrolase (MutT family)	1.38	0.008	96.39	69.76
SMU_562	clpE	ATP-dependent protease ClpE	0.85	0.002	1616.69	1891.11
SMU_563		ornithine carbamoyltransferase	0.45	0.000	22.86	50.76
SMU_564		hypothetical protein	0.51	0.000	370.85	723.11
SMU_565c		transposase, ISSmu1	2.63	0.000	29.98	11.39
SMU_566c		hypothetical protein	2.02	0.068	13.87	6.86
SMU_567		glutamine ABC transporter permease	0.61	0.000	252	412.14
SMU_568		amino acid ABC transporter ATP-binding protein	1.03	0.580	357.61	346.12
SMU_569	feoA	ferrous ion transport protein A	0.60	0.000	65.06	109.17
SMU_570	feoB	ferrous ion transport protein B	0.92	0.760	208.31	225.69
SMU_571		hypothetical protein	0.79	0.013	151.42	191.57
SMU_572	folD	bifunctional 5,10-methylene-tetrahydrofolate dehydrogenase/ 5,10-methyl	0.65	0.000	433.94	670.63
SMU_573		hypothetical protein	1.03	0.810	207.04	201.99
SMU_574c	lrgB	hypothetical protein	7.92	0.000	43.07	5.44
SMU_575c		hypothetical protein	0.83	0.950	12.14	14.59
SMU_576	lytR	response regulator LytR	1.85	0.001	48.59	26.32
SMU_577	lytS	histidine kinase LytS	1.37	0.100	40.93	29.88
SMU_580		exodeoxyribonuclease VII large subunit	0.80	0.047	126.19	157.74
SMU_581		exodeoxyribonuclease VII small subunit	0.82	0.021	249.37	304.43
SMU_582		farnesyl diphosphate synthase	0.86	0.280	278.36	322.83
SMU_583		hemolysin	0.89	0.760	129.07	144.69
SMU_584		arginine repressor ArgR	0.89	0.680	155.34	175.02
SMU_585	recN	DNA repair protein RecN	1.00	1.000	291.17	292.19
SMU_586		hypothetical protein	0.96	0.810	409.67	428.54
SMU_587		hypothetical protein	1.53	0.000	241.57	158.37
SMU_588		hypothetical protein	0.86	0.460	119.57	139.67
SMU_589		DNA-binding protein	0.53	0.000	1857.6	3508.05
SMU_590c		transposase, fragment	0.98	0.980	307.52	315
SMU_591c		hypothetical protein	0.70	0.110	28.43	40.69
SMU_592c		transcriptional regulator	1.15	0.740	15.32	13.3
SMU_593	furR	ferric uptake regulator protein FurR	0.75	0.009	101.21	134.83
SMU_594		hypothetical protein		0.000	27.61	0
SMU_595	pyrD	dihydroorotate dehydrogenase 1A	1.10	0.380	111.49	101.24
SMU_596	pmgY	2,3-bisphosphoglycerate-dependent phosphoglycerate mutase	0.74	0.000	1900.83	2563.52
SMU_597	pbp2b	penicillin-binding protein 2b	1.05	0.260	568.47	539.9
SMU_598	recM	recombination protein RecR	0.93	0.610	635.21	683.19

SMU_599		D-alanyl-alanine synthetase A	0.80	0.009	222.21	276.16
SMU_600c		hypothetical protein	0.54	0.000	231.57	428.62
SMU_602		sodium-dependent transporter	0.54	0.000	110.3	204.07
SMU_603	murF	UDP-N-acetylmuramoylalanyl-D-glutamyl-2,6- diaminopimelate-D-alanyl-	0.94	0.930	147.69	156.84
SMU_604		hypothetical protein	0.40	0.072	5.42	13.41
SMU_605		hypothetical protein		0.029	2.78	0
SMU_606		hypothetical protein	1.35	0.180	32.73	24.28
SMU_607		hypothetical protein	0.77	0.060	65.53	84.66
SMU_608		peptide chain release factor 3	0.93	0.790	273.97	295.25
SMU_609		40K cell wall protein	1.40	0.001	147.02	105.39
SMU_610	spaP	cell surface antigen SpaP	0.93	0.440	1339.07	1438.29
SMU_611		ATP-dependent RNA helicase, DEAD-box family	0.68	0.000	2468.19	3609.03
SMU_613		hypothetical protein	0.85	0.110	290.27	343.21
SMU_614		hypothetical protein	1.23	0.012	211.45	172.55
SMU_616		hypothetical protein	1.17	0.000	995	852.55
SMU_618		hypothetical protein	0.65	0.000	2143.88	3291.43
SMU_620		hypothetical protein	2.64	0.000	70.75	26.85
SMU_621c		hypothetical protein	0.95	0.900	194.17	203.66
SMU_622c		hypothetical protein	1.34	0.007	118.95	88.75
SMU_623c		deacetylase	0.77	0.007	119.09	155.36
SMU_624		1-acylglycerol-3-phosphate O-acyltransferase	0.76	0.000	303.07	396.42
SMU_625	comEA	competence protein	0.72	0.000	4811.63	6692.66
SMU_626		competence protein	0.90	0.088	2764.24	3072.64
SMU_627		hypothetical protein	1.11	0.000	2503.73	2255.17
SMU_628		DNA polymerase III subunit delta	0.75	0.054	56	74.35
SMU_629	sod	manganese-type superoxide dismutase, Fe/Mn-SOD	0.44	0.000	1682.75	3809.97
SMU_630		hypothetical protein	0.83	0.150	136.06	163.84
SMU_631		hypothetical protein	0.76	0.220	29.59	38.8
SMU_632		transcriptional regulator	0.37	0.000	83.95	226.43
SMU_633		thioesterase	0.72	0.001	130.42	180.34
SMU_634	queA	S-adenosylmethionine--tRNA ribosyltransferase-isomerase	0.61	0.000	416.14	685.55
SMU_635		hypothetical protein	0.67	0.000	1455.41	2162.28
SMU_636		N-acetylglucosamine-6-phosphate isomerase	0.57	0.000	1156.6	2033.28
SMU_637c		hypothetical protein	0.68	0.000	319.65	469.75
SMU_638		16S rRNA pseudouridine(516) synthase	0.86	0.310	279.41	323.46
SMU_639		acetyltransferase	0.86	0.210	333.14	387.37
SMU_640c		GntR family transcriptional regulator	0.77	0.006	132.48	171.89
SMU_641		oxidoreductase	0.85	0.470	93.07	109.25
SMU_642		hypothetical protein	7.96	0.000	48.26	6.06
SMU_643		esterase	1.08	0.580	93.1	86.58
SMU_644		competence protein/transcription factor	0.83	0.000	984.77	1181.44
SMU_645	pepB	oligopeptidase	0.79	0.000	772.6	978.02
SMU_646		phosphatase	0.96	0.670	1219.11	1271.09
SMU_647		methyltransferase	0.93	0.670	432.19	465.63
SMU_648	prtM	foldase PrsA	0.85	0.019	641	757.7
SMU_649		hypothetical protein	1.28	0.001	266.3	207.31
SMU_650		alanyl-tRNA synthetase	1.07	0.190	498.3	466.77
SMU_651c		ABC transporter substrate-binding protein	0.79	0.410	23.58	30.01
SMU_652c		nitrate ABC transporter ATP-binding protein	1.37	0.160	36.05	26.37
SMU_653c		ABC transporter permease	1.02	1.000	26.4	25.95
SMU_654	mutF	ABC transporter ATP-binding protein MutF	2.56	0.002	20.14	7.87
SMU_655	mutE1	protein MutE	0.58	0.420	6.32	10.86
SMU_656	mutE2	protein MutE	1.15	0.810	9.79	8.55
SMU_657	mutG	MutG	2.72	0.000	24.41	8.96
SMU_658		hypothetical protein	0.31	0.002	7.75	25.07
SMU_659		response regulator SpaR	1.07	0.840	25.75	24.14
SMU_660		histidine kinase SpaK	0.92	1.000	32.13	34.98
SMU_661		transcriptional regulator	3.37	0.000	97.37	28.92
SMU_662		hypothetical protein	1.06	0.770	62.19	58.56
SMU_663	argC	N-acetyl-gamma-glutamyl-phosphate reductase	1.20	0.340	49.4	41.17
SMU_664	argJ	bifunctional ornithine acetyltransferase/N-acetylglutamate synthase	2.08	0.000	67.88	32.65
SMU_665	argB	acetylglutamate kinase	1.71	0.001	57.26	33.54
SMU_666	argD	acetylornithine aminotransferase	1.83	0.000	184.38	100.84
SMU_667	nrdG	ribonucleotide-diphosphate reductase subunit beta	1.15	0.000	806.64	699.84
SMU_668c		ribonucleotide-diphosphate reductase subunit alpha	1.04	0.430	458.75	439.88
SMU_669c		glutaredoxin	0.47	0.000	373.39	789.19
SMU_670	citB	aconitate hydratase	0.89	0.590	318.92	356.91
SMU_671	citZ	citrate synthase	1.27	0.000	396.64	313.43
SMU_672	idh	isocitrate dehydrogenase	1.23	0.000	425.74	346.56
SMU_673		hypothetical protein	1.24	0.003	255.56	206.47
SMU_674	ptsH	phosphocarrier protein HPr	0.88	0.034	2790.22	3166.83
SMU_675		PTS system transporter protein EI	1.00	0.970	2866.53	2877.79
SMU_676	gapN	NADP-dependent glyceraldehyde-3-phosphate dehydrogenase	0.85	0.000	2096.38	2475.57
SMU_677		MerR family transcriptional regulator	15.12	0.000	54.75	3.62
SMU_678		oxidoreductase	0.83	0.330	81.02	97.21
SMU_679		oxidoreductase	1.08	0.610	103.91	96.66
SMU_680		gamma-carboxymuconolactone decarboxylase subunit	2.53	0.000	54.18	21.4
SMU_681		hypothetical protein	0.69	0.002	89.24	128.51
SMU_682		hypothetical protein	1.25	0.520	20.49	16.45
SMU_683		ATP-binding protein	1.32	0.091	57.42	43.51
SMU_684		hypothetical protein	1.45	0.048	46.65	32.17
SMU_685		hypothetical protein	0.94	1.000	14.68	15.57
SMU_687c		hypothetical protein	0.00	0.000	0	37.94
SMU_688		hypothetical protein	0.94	1.000	60.74	64.57
SMU_689	atlA	hypothetical protein	1.09	0.160	360.19	331.61
SMU_690		hypothetical protein	0.79	0.000	414.63	522.18
SMU_691	pepT	peptidase T	1.38	0.001	162.54	117.69
SMU_692		hypothetical protein	0.67	0.000	193.83	289.24
SMU_694c		ferredoxin (4Fe-4S)	0.90	0.720	178.81	197.74
SMU_695		hypothetical protein	1.01	0.890	215.55	212.39
SMU_696		cytidylate kinase	0.80	0.000	638.44	799.19

SMU_697		translation initiation factor IF-3	0.86	0.028	1202.23	1392.26
SMU_698		50S ribosomal protein L35	0.89	0.087	2048.8	2313.3
SMU_699		50S ribosomal protein L20	0.54	0.000	1429.56	2631.4
SMU_700c		phosphoglycerate mutase	1.23	0.015	195.47	159.53
SMU_701c		hypothetical protein	1.40	0.001	143.9	102.57
SMU_702c		transcriptional regulator	0.95	0.880	190.52	200.52
SMU_703c		hypothetical protein	0.95	0.980	146.66	154.77
SMU_704c		autolysin; amidase	0.62	0.010	34.15	55.52
SMU_706c		hypothetical protein	1.04	0.810	75.66	72.65
SMU_707c		endolysin	0.82	0.000	859.15	1047.75
SMU_709		hypothetical protein	0.81	0.017	226.03	278.87
SMU_711		hypothetical protein	0.98	1.000	164.08	167.45
SMU_712	capP	phosphoenolpyruvate carboxylase	1.10	0.220	225.23	205.59
SMU_713	ftsW	cell division protein FtsW	0.98	0.930	408.1	417.71
SMU_714		elongation factor Tu	0.80	0.000	11827.48	14872.12
SMU_715		triosephosphate isomerase	0.67	0.000	1841.36	2745.15
SMU_716	murN	peptidoglycan branched peptide synthesis proteinMurN	0.83	0.095	169.76	205.29
SMU_717	murM	peptidoglycan branched peptide synthesis proteinMurM	1.10	0.190	211.62	191.58
SMU_718c		hypothetical protein	1.67	0.000	230.78	138.59
SMU_719c		hypothetical protein	0.81	0.120	86.77	107.06
SMU_720		Na+/solute symporter	0.98	1.000	152.52	156.37
SMU_721		hypothetical protein	1.16	0.170	116.44	100.64
SMU_722		hypothetical protein	1.08	0.790	27.32	25.29
SMU_723		cadmium-transporting ATPase	0.93	0.590	725.61	778.89
SMU_724		glycerophosphoryl diester phosphodiesterase	0.83	0.530	46.02	55.3
SMU_725c		hypothetical protein	0.85	0.480	83.38	98.58
SMU_727		transcriptional regulator	1.84	0.002	44.35	24.09
SMU_728		oxidoreductase	1.20	0.130	97.19	80.87
SMU_730		hypothetical protein	0.97	1.000	13.02	13.36
SMU_731		ABC transporter ATP-binding protein	1.01	1.000	24.64	24.39
SMU_732		hypothetical protein	0.73	0.110	31.42	43.28
SMU_734		hypothetical protein	0.71	0.021	57.71	81.5
SMU_735		hypothetical protein	0.53	0.000	186.83	350.33
SMU_737		hypothetical protein	0.55	0.000	100.25	183.59
SMU_738		hypothetical protein	2.02	0.000	102.26	50.59
SMU_739c		hypothetical protein	1.09	0.700	48.05	44.22
SMU_741		hypothetical protein	0.84	0.390	71.29	85.18
SMU_742		hypothetical protein	0.73	0.002	113.15	154.95
SMU_743		hypothetical protein	1.42	0.000	196.07	138.48
SMU_744	ftsY	cell division protein FtsY	1.48	0.000	345.15	232.76
SMU_745	lmrB	drug-export protein; multidrug resistance protein	0.75	0.019	73.97	99.24
SMU_746c		hypothetical protein	0.64	0.000	199.38	310.37
SMU_747c		permease	0.74	0.000	247.63	332.62
SMU_748		hypothetical protein	0.58	0.000	302.32	517.68
SMU_750c		hypothetical protein		1.000	0	0
SMU_751		transcriptional accessory protein	0.86	0.230	356.83	413.34
SMU_752		hypothetical protein	0.80	0.004	232.09	290.73
SMU_753		hypothetical protein	0.37	0.000	2569.98	6896.84
SMU_754		HPr kinase/phosphorylase	0.73	0.000	1580.6	2159.15
SMU_755		prolipoprotein diacylglyceryl transferase	0.87	0.061	1029.02	1186.95
SMU_756		hypothetical protein	0.70	0.000	533.33	756.68
SMU_757		hypothetical protein	0.52	0.000	385.91	735.43
SMU_758c		hypothetical protein	0.30	0.000	76.75	258.87
SMU_759		protease	0.87	0.350	342.62	393.23
SMU_761		protease	0.84	0.008	608.63	726.55
SMU_764	ahpC	alkyl hydroperoxide reductase	1.15	0.001	824.44	718.25
SMU_765		alkyl hydroperoxide reductase	1.19	0.000	1348.9	1136.95
SMU_766		hypothetical protein	1.34	0.450	15.84	11.79
SMU_767		transposase, ISSmu1	2.09	0.003	29.53	14.11
SMU_768c		hypothetical protein	1.30	0.500	17.5	13.42
SMU_769		hypothetical protein	0.58	0.000	3329.77	5744.68
SMU_770c		manganese transporter	0.74	0.000	966.18	1304.82
SMU_771c		hypothetical protein	1.70	0.000	760.72	446.87
SMU_772	gbpD	glucan-binding protein D	0.82	0.001	503.8	616.48
SMU_773c		lysyl-tRNA synthetase	1.07	0.280	361.33	338.2
SMU_774		hypothetical protein	0.71	0.000	221.09	311.98
SMU_775c		hypothetical protein	0.80	0.002	306.47	382.19
SMU_776		hypothetical protein	0.61	0.000	119.06	194.99
SMU_777	aroD	3-dehydroquinase	0.82	0.014	282.63	345.42
SMU_778	aroE	shikimate 5-dehydrogenase	1.30	0.000	322.3	247.44
SMU_779	aroB	3-dehydroquinate synthase	1.46	0.000	426.08	292.29
SMU_780	aroC	chorismate synthase	1.24	0.000	636.97	515.72
SMU_781		prephenate dehydrogenase	1.01	0.860	525.25	519.39
SMU_782		hypothetical protein	1.06	0.220	487.17	458.05
SMU_784	aroA	3-phosphoshikimate 1-carboxyvinyltransferase	0.87	0.690	114.76	131.42
SMU_785	aroK	shikimate kinase	1.27	0.028	123.03	96.95
SMU_786	pheA	prephenate dehydratase	0.94	0.990	75.59	80.03
SMU_787		transcriptional regulator	1.27	0.002	236.59	186.94
SMU_788		RNA methyltransferase	1.09	0.210	266.05	244.48
SMU_789		hypothetical protein	0.55	0.000	260.73	476.5
SMU_790		hypothetical protein	1.41	0.530	10	7.11
SMU_791c		hypothetical protein	4.98	0.000	11.8	2.37
SMU_793		hypothetical protein	1.03	0.740	196.91	190.43
SMU_794		hypothetical protein	0.71	0.000	133.14	186.7
SMU_795		esterase	0.66	0.000	122.33	185.52
SMU_796		hypothetical protein	1.07	0.470	159.61	149.11
SMU_797		hypothetical protein	1.98	0.024	20.57	10.39
SMU_798c		hypothetical protein	0.46	0.000	82.93	180.88
SMU_799c		hypothetical protein	1.28	0.062	84.4	65.85
SMU_800		hypothetical protein	1.17	0.200	99.95	85.49
SMU_802		hypothetical protein	1.45	0.000	196.99	136.06

SMU_803c		ABC transporter ATP-binding protein	0.96	0.950	265.2	275.96
SMU_804		hypothetical protein	1.39	0.130	39.69	28.57
SMU_805c		amino acid ABC transporter ATP-binding protein	1.01	0.870	1246.35	1239.25
SMU_806c		glutamine ABC transporter permease	0.70	0.000	627.65	893.5
SMU_807		hypothetical protein	0.92	0.850	114.65	124.61
SMU_809	uvrB	excinuclease ABC subunit B	1.36	0.001	182.13	133.99
SMU_811		hypothetical protein	2.92	0.000	74.03	25.39
SMU_812		hypothetical protein	9.20	0.000	34.5	3.75
SMU_813		transcriptional regulator	1.38	0.038	61.47	44.4
SMU_814	mutT	MutT-like protein	1.22	0.008	239.5	196.44
SMU_815		amino acid ABC transporter substrate-binding protein	1.47	0.000	267.62	181.69
SMU_816		transaminase	0.77	0.000	235.65	304.71
SMU_817		amino acid ABC transporter substrate-binding protein	0.70	0.000	129.18	183.96
SMU_818		30S ribosomal protein S21	0.78	0.004	153.33	197.75
SMU_819	mscL	large conductance mechanosensitive channel	0.40	0.000	642.02	1622.9
SMU_820		hypothetical protein	0.98	0.960	580.32	591.58
SMU_821	dnaG	DNA primase	1.06	0.440	257.41	242.66
SMU_822	rpoD	RNA polymerase sigma factor RpoD	1.23	0.000	786.85	642.18
SMU_823		hypothetical protein	1.01	0.860	678.13	672.03
SMU_824		dTDP-4-keto-L-rhamnose reductase	1.06	0.320	423.5	401.05
SMU_825	rgpA	RgpAc; glycosyltransferase	0.70	0.000	466.8	664.63
SMU_826	rgpB	rhamnosyltransferase	1.06	0.310	331.8	311.93
SMU_827	rgpC	polysaccharide ABC transporter permease	1.27	0.000	332.99	261.73
SMU_828	rgpD	polysaccharide ABC transporter ATP-binding protein	1.09	0.140	399.93	367.5
SMU_829	rgpE	glycosyltransferase	1.16	0.020	318.99	275
SMU_830	rgpF	RgpFc protein	0.87	0.420	300.72	343.84
SMU_831		hypothetical protein	1.14	0.120	183	160.48
SMU_832		hypothetical protein	1.28	0.003	202.55	158.17
SMU_833		glycosyltransferase	1.72	0.000	126.67	73.83
SMU_834		hypothetical protein	1.07	0.290	341.55	320.63
SMU_835		hypothetical protein	1.36	0.001	187.7	138.51
SMU_836	lytF	hypothetical protein	0.97	0.610	3136.63	3239.49
SMU_837		reductase	0.85	0.000	3651.84	4274.17
SMU_838	gshR	glutathione reductase	0.91	0.310	1330.43	1456.77
SMU_839	fol	folyl-polyglutamate synthetase	0.76	0.037	66.95	88.41
SMU_840c		hypothetical protein	0.37	0.000	274.01	738.47
SMU_841		aminotransferase	0.97	0.960	250.55	257.43
SMU_842	thiI	thiamine biosynthesis protein ThiI	1.03	0.540	430.43	416.56
SMU_843		hypothetical protein	0.80	0.051	103.46	129.88
SMU_844		hypothetical protein	1.73	0.000	86.63	50.1
SMU_845		hypothetical protein	1.62	0.000	105.12	64.85
SMU_847c		hypothetical protein	0.81	0.000	1326.68	1630.9
SMU_846		50S ribosomal protein L21	0.79	0.000	1339.13	1699.84
SMU_848		hypothetical protein	0.74	0.000	1850.74	2487.94
SMU_849		50S ribosomal protein L27	3.57	0.000	522.89	146.63
SMU_850		hypothetical protein	0.57	0.000	93.33	164.77
SMU_851		hypothetical protein	0.59	0.000	70.15	119.73
SMU_852		transcriptional regulator; CpsY-like protein	0.73	0.000	173.72	238.54
SMU_853	lspA	lipoprotein signal peptidase	0.82	0.370	46.95	57.28
SMU_854		pseudouridylate synthase	1.25	0.005	216.35	173.26
SMU_855		hypothetical protein	0.78	0.000	366.56	471.29
SMU_856	pyrR	bifunctional pyrimidine regulatory protein PyrR/uracil phosphoribosyltrans	1.94	0.000	139.29	71.81
SMU_857		uracil permease	1.64	0.000	166.55	101.71
SMU_858	pyrB	aspartate carbamoyltransferase	1.43	0.000	191.55	134.3
SMU_859	pyrA	carbamoyl phosphate synthase small subunit	1.83	0.000	295.48	161.49
SMU_860	pyrAB	carbamoyl phosphate synthase large subunit	1.27	0.000	414.81	326.62
SMU_862		permease	0.78	0.000	250.21	322.29
SMU_863		ABC transporter ATP-binding protein	0.82	0.004	431.91	526.72
SMU_864		ABC transporter permease	0.89	0.520	327.36	369.47
SMU_865		30S ribosomal protein S16	0.68	0.000	1813.28	2659.01
SMU_866		hypothetical protein	0.66	0.000	1109.66	1676.54
SMU_867	rimM	16S rRNA-processing protein RimM	1.85	0.000	74.98	40.57
SMU_868	trmD	tRNA (guanine-N(1)-)-methyltransferase	1.18	0.099	137.94	117.29
SMU_869	trxB2	thioredoxin reductase	0.74	0.000	160.28	217.07
SMU_870		sugar metabolism transcriptional regulator	0.99	1.000	240.2	243.38
SMU_871	pfkB	1-phosphofructokinase	0.97	0.960	361.69	371.3
SMU_872		PTS system fructose-specific transporter subunitIIABC	0.94	0.680	646.43	685.96
SMU_873	metE	5- methyltetrahydropteroyltriglutamate/homocysteine S-methyltransferase	2.13	0.054	12.99	6.1
SMU_874		bifunctional homocysteine S-methyltransferase/5,10-methylenetetrahydro	1.29	0.330	26.13	20.22
SMU_875c		transposase, IS150-like	1.17	0.670	15.59	13.35
SMU_876	msmR	MSM operon regulatory protein	1.97	0.006	29.51	14.96
SMU_877	agaL	alpha-galactosidase	1.15	0.450	52.96	46.25
SMU_878	msmE	multiple sugar-binding ABC transporter, sugar-binding protein MsmE	1.55	0.011	51.09	33.01
SMU_879	msmF	multiple sugar-binding ABC transporter permease MsmF	2.02	0.000	41.45	20.53
SMU_880	msmG	multiple sugar-binding ABC transporter permease MsmG	0.98	1.000	55.41	56.3
SMU_881	gtfA	sucrose phosphorylase GtfA	1.54	0.000	141.02	91.51
SMU_882	msmK	multiple sugar-binding ABC transporter ATP-binding protein, MsmK	0.95	0.930	167.76	176.15
SMU_883	dexB	dextran glucosidase DexB	0.82	0.120	120.68	146.78
SMU_885	galR	LacI family transcriptional repressor	1.72	0.000	86.65	50.4
SMU_886	galK	galactokinase	1.68	0.001	67.6	40.27
SMU_887	galT	galactose-1-phosphate uridylyltransferase	1.31	0.015	118.68	90.61
SMU_888	galE	UDP-galactose 4-epimerase GalE	1.17	0.008	362.65	309.41
SMU_889	pbpX	penicillin-binding protein, class C; fmt-like protein	0.78	0.000	604.54	779.79
SMU_890		hypothetical protein	1.08	0.610	103.15	95.9
SMU_891	hsdM	type I restriction-modification system DNA methylase	1.24	0.100	80.04	64.36
SMU_892	hsdS	type I restriction-modification system, specificity determinant	1.24	0.110	84.62	68.31
SMU_893		anticodon nuclease	1.39	0.022	69.42	49.91
SMU_895		DNA-damage-inducible protein	0.48	0.000	52.37	109.97
SMU_896		hypothetical protein	0.98	1.000	67.17	68.84
SMU_897		type I restriction-modification system, helicasesubunits	1.10	0.690	40.25	36.69
SMU_898		hypothetical protein	0.72	0.000	301.13	415.84

SMU_899		hypothetical protein	0.94	0.810	308.19	329.29
SMU_900	dapB	4-hydroxy-tetrahydrodipicolinate reductase	0.83	0.019	344.67	416.66
SMU_901	papS	tRNA CCA-pyrophosphorylase	1.00	1.000	153.35	153.84
SMU_902		ABC transporter ATP-binding protein	1.04	0.710	158.69	152.33
SMU_905		ABC transporter ATP-binding protein	0.99	1.000	184.46	185.85
SMU_906		ABC transporter ATP-binding protein	0.83	0.140	164.76	197.33
SMU_909		permease	1.32	0.150	45.78	34.61
SMU_910	gtfD	glucosyltransferase-S	0.93	0.850	171.83	184.13
SMU_911c		hypothetical protein	0.70	0.017	52.14	74.77
SMU_913		glutamate dehydrogenase	0.69	0.000	847.42	1232.68
SMU_914c		hypothetical protein	1.02	0.890	138.59	135.61
SMU_915c		7-cyano-7-deazaguanine reductase	1.03	0.780	137.62	132.97
SMU_916c		7-cyano-7-deazaguanosine (preQ0) biosynthesis protein QueE	1.35	0.000	211.66	157.25
SMU_917c		6-pyruvoyl tetrahydrobiopterin synthase	1.05	0.710	107.75	102.18
SMU_919c		7-cyano-7-deazaguanine synthase QueC	1.85	0.000	130	70.27
SMU_921		transcriptional regulator	0.71	0.000	162.75	230.59
SMU_922		ABC transporter ATP-binding protein	1.06	0.350	315.07	297.11
SMU_923		ABC transporter ATP-binding protein	0.82	0.030	225.45	275.16
SMU_924	tpx	2-Cys peroxiredoxin	0.44	0.000	592.5	1350.21
SMU_925	cipI	hypothetical protein	0.24	0.000	1668.83	7016.78
SMU_926		GTP-pyrophosphokinase	0.59	0.000	852.07	1432.61
SMU_927		response regulator	0.75	0.000	508.52	677.65
SMU_928		histidine kinase	0.61	0.000	394.45	644.57
SMU_929c		hypothetical protein	0.31	0.000	212.96	681
SMU_930c		transcriptional regulator	1.14	0.780	12.12	10.61
SMU_932		hypothetical protein	1.78	0.003	42.3	23.71
SMU_933		amino acid ABC transporter substrate-binding protein	1.39	0.130	39.68	28.62
SMU_934		amino acid ABC transporter permease	1.45	0.010	74.33	51.13
SMU_935		amino acid ABC transporter permease	3.80	0.000	50.52	13.28
SMU_936		amino acid ABC transporter ATP-binding protein	1.71	0.000	79.48	46.49
SMU_937		mevalonate diphosphate decarboxylase	0.85	0.370	93.18	110.26
SMU_938		phosphomevalonate kinase	0.82	0.140	118.98	144.27
SMU_939		isopentenyl pyrophosphate isomerase	1.05	0.720	110.36	105.14
SMU_940c		hemolysin III	0.52	0.000	631.96	1226.82
SMU_941c		hypothetical protein	0.38	0.000	846.68	2201.19
SMU_942	mvaA	hydroxymethylglutaryl-CoA reductase	0.72	0.000	459.72	641.59
SMU_943c		hydroxymethylglutaryl-CoA synthase	0.80	0.006	222.1	277.79
SMU_944	thyA	thymidylate synthase	0.61	0.000	251.3	412.61
SMU_946		permease	1.81	0.000	104.94	58
SMU_947	dfrA	dihydrofolate reductase	1.67	0.000	243.05	145.89
SMU_948		hypothetical protein	1.13	0.068	303.73	269.59
SMU_949	clpX	ATP-dependent protease ATP-binding subunit ClpX	0.99	0.970	814.24	820.57
SMU_950		GTP-binding protein YsxC	0.88	0.260	834.82	946.86
SMU_951		amino acid permease	0.69	0.000	219.27	316.8
SMU_952		homocysteine methyltransferase	0.66	0.000	194.49	296.83
SMU_953c		transcriptional regulator/aminotransferase	1.71	0.005	42.68	25.01
SMU_954		pyridoxamine kinase	0.55	0.000	50.11	90.34
SMU_955		hypothetical protein	1.19	0.430	41.99	35.17
SMU_956	clp	Clp-like ATP-dependent protease, ATP-binding subunit	1.18	0.000	930.8	791.73
SMU_957		50S ribosomal protein L10	0.55	0.000	1566.98	2849.66
SMU_958		hypothetical protein	0.58	0.000	1175.77	2043.21
SMU_959c		hypothetical protein	0.88	0.110	1672.67	1896.34
SMU_960		50S ribosomal protein L7/L12	0.84	0.000	1421.93	1697.86
SMU_961		hypothetical protein	0.82	0.053	164.52	201.83
SMU_962		dehydrogenase	0.98	1.000	142.71	145.35
SMU_963c		deacetylase	0.98	0.980	313.7	319.34
SMU_965		homoserine dehydrogenase	0.87	0.560	157.39	180.31
SMU_966		homoserine kinase	1.38	0.000	303.05	219.74
SMU_967	folC	folyl-polyglutamate synthetase	1.02	0.850	171.26	167.33
SMU_968		GTP cyclohydrolase I	0.98	0.960	451.44	461.24
SMU_969	folP	dihydropteroate synthase	1.30	0.000	450.75	345.84
SMU_970	folA	dihydroneopterin aldolase	1.20	0.032	182.57	152.08
SMU_971	folK	2-amino-4-hydroxy-6- hydroxymethyldihydropteridine pyrophosphokinase	1.30	0.001	222.76	171.03
SMU_972	murB	UDP-N-acetylenolpyruvoylglucosamine reductase	0.78	0.002	194.48	248.42
SMU_973	potA	spermidine/putrescine ABC transporter ATP-binding protein	0.97	0.950	377.63	388.16
SMU_974	potB	spermidine/putrescine ABC transporter permease	1.65	0.000	126.92	76.96
SMU_975	potC	spermidine/putrescine ABC transporter permease	0.99	1.000	94.31	95.22
SMU_976	potD	ABC transporter periplasmic spermidine/putrescine-binding protein	1.33	0.004	143.65	107.7
SMU_977	licT	transcriptional antiterminator LicT (fragment)	0.76	0.041	69.58	91.06
SMU_980	bglP	PTS system beta-glucoside-specific transporter subunit II	1.44	0.290	17.73	12.35
SMU_981	bglB1	BglB fragment	6.06	0.008	5.82	0.96
SMU_982	bglB2	BglB fragment	2.19	0.460	3.35	1.53
SMU_983	bglC	transcriptional regulator	1.22	0.570	17.58	14.45
SMU_984		hypothetical protein	0.60	0.000	84.63	141.43
SMU_985	bglA	beta-glucosidase	1.15	0.270	95.57	83.39
SMU_986c		hypothetical protein	1.82	0.029	24.09	13.24
SMU_987	wapA	cell wall-associated protein WapA	0.71	0.000	352.03	494.12
SMU_988		cardiolipin synthase	0.71	0.024	54.92	77.41
SMU_989	asd	aspartate-semialdehyde dehydrogenase	0.69	0.000	652.03	950.39
SMU_990	dapA	4-hydroxy-tetrahydrodipicolinate synthase	0.79	0.000	348.06	441.04
SMU_991		ribonucleotide reductase	0.47	0.000	655.22	1398.13
SMU_992		hypothetical protein	0.88	0.990	49.26	55.67
SMU_993		GTPase YlqF	0.91	0.710	218.82	239.47
SMU_994	rnh	ribonuclease HII	0.96	0.890	312.04	325.63
SMU_995		ferrichromeABC transporter permease	1.14	0.190	142.38	125
SMU_996		ferrichromeABC transporter permease	1.29	0.007	154.84	119.74
SMU_997		inorganic ion ABC transporter ATP-binding protein	1.35	0.000	296.09	219.5
SMU_998		ABC transporter periplasmic ferrichrome-binding protein	1.04	0.640	264.02	254.1
SMU_999		hypothetical protein	0.03	0.000	1.89	65.59
SMU_1000		hypothetical protein	0.00	0.180	0	2.26
SMU_1001	smf	DNA processing Smf protein	1.14	0.000	1253.55	1099.09

SMU_1002	topA	DNA topoisomerase I	0.97	0.610	2941.51	3037.62
SMU_1003	gid	tRNA (uracil-5-)-methyltransferase Gid	1.01	0.740	2399.42	2381.37
SMU_1004	gtfB	glucosyltransferase-I	1.15	0.021	349.35	304.15
SMU_1005	gtfC	glucosyltransferase-SI	1.00	0.950	556.16	554.01
SMU_1006		ABC transporter ATP-binding protein	2.07	0.000	57.14	27.59
SMU_1007		ABC transporter permease	0.91	0.950	33.44	36.78
SMU_1008		response regulator	0.93	0.910	137.78	148.85
SMU_1009		histidine kinase	0.87	0.880	34.46	39.52
SMU_1010	citC	[citrate (pro-3S)-lyase] ligase	2.70	0.000	41.66	15.45
SMU_1011	citG	CitG protein	1.55	0.039	34.85	22.51
SMU_1012c		transcriptional regulator	0.70	0.120	24.89	35.67
SMU_1013c		Mg2+/citrate transporter	0.66	0.041	30.45	46.42
SMU_1014		hypothetical protein	1.97	0.000	65.4	33.24
SMU_1016	bcc	acetyl-CoA carboxylase biotin carboxyl carrier protein subunit	1.55	0.000	99.83	64.45
SMU_1017	oadB	oxaloacetate decarboxylase, sodium ion pump subunit	1.13	0.500	50.69	45.01
SMU_1018		hypothetical protein	2.34	0.000	98.18	41.91
SMU_1019	cilG	citrate lyase subunit gamma	3.45	0.000	130.34	37.76
SMU_1020	cilB	citrate lyase CilB, citryl-CoA lyase subunit beta	1.46	0.009	74.77	51.05
SMU_1021	cilA	citrate lyase, alfa subunit	1.35	0.110	46.96	34.83
SMU_1022	citG2	2-(5''-triphosphoribosyl)-3'-dephosphocoenzyme-Asynthase	1.00	1.000	75.37	75.36
SMU_1023	pycB	oxaloacetate decarboxylase	1.02	0.930	74.07	72.68
SMU_1024c		transposase fragment	0.41	0.350	2.91	7.03
SMU_1025		transcriptional regulator	0.80	0.910	5.35	6.68
SMU_1026		hypothetical protein		0.000	4.75	0
SMU_1027		transcriptional regulator		0.000	6.34	0
SMU_1028		hydrolase	1.18	0.360	49.22	41.79
SMU_1029		hypothetical protein	1.33	0.540	12.72	9.56
SMU_1030		polyribonucleotide nucleotidyltransferase		0.000	15.75	0
SMU_1031	xis	transposon excisionase; Tn916 ORF1-like		0.640	1.54	0
SMU_1032	tnr5	transposon integrase; Tn916 ORF3-like	1.29	0.340	27	20.97
SMU_1034c		site-specific tyrosine recombinase XerS	0.56	0.000	84.69	150.38
SMU_1035	glrA	ABC transporter ATP-binding protein	0.66	0.160	15.92	24.19
SMU_1036		hypothetical protein	1.18	0.640	17.61	14.92
SMU_1037c		histidine kinase	0.85	0.450	97.59	114.76
SMU_1038c		response regulator	1.31	0.003	173.39	132.83
SMU_1039c		lipopolysaccharide glycosyltransferase	1.68	0.001	68.41	40.6
SMU_1040c		oxidoreductase	1.11	0.240	174.06	156.96
SMU_1041		ABC transporter ATP-binding protein	0.94	0.900	95.17	101.71
SMU_1042		hypothetical protein	0.83	0.480	53.19	63.71
SMU_1043c		phosphotransacetylase	0.76	0.000	694.68	919.98
SMU_1044c		pseudouridylate synthase	0.93	0.690	441.19	475.71
SMU_1045c		inorganic polyphosphate/ATP-NAD kinase	0.99	1.000	395.21	397.5
SMU_1046c		GTP pyrophosphokinase	0.63	0.000	359.51	567.63
SMU_1047c		hypothetical protein	0.64	0.000	863.49	1345.09
SMU_1048		hypothetical protein	0.50	0.000	73.39	146.29
SMU_1050	krpS	ribose-phosphate pyrophosphokinase	0.91	0.570	497.03	545.91
SMU_1051		iron-sulfur cofactor synthesis protein; NifS family	0.94	0.770	418.9	443.73
SMU_1052		hypothetical protein	1.05	0.590	219.95	210.16
SMU_1053		redox-sensing transcriptional repressor Rex	0.83	0.000	1561.04	1874.7
SMU_1054		glutamine amidotransferase	0.96	0.580	1816.53	1900.55
SMU_1055	radC	hypothetical protein	0.84	0.001	998.7	1192.45
SMU_1056		hypothetical protein	0.53	0.000	122.43	230.03
SMU_1057	satE	hypothetical protein	1.09	0.460	117.57	107.38
SMU_1058	satD	hypothetical protein	0.95	0.900	182.49	192.89
SMU_1059	satC	hypothetical protein	1.26	0.310	31.66	25.06
SMU_1060	ffh	signal recognition particle protein	1.03	0.570	571.93	556.74
SMU_1061	ylxM	DNA-binding protein	0.39	0.000	97.56	250.38
SMU_1062	opuAb	ABC transporter proline/glycine betaine permease	1.04	0.390	555.13	532.88
SMU_1063	opuAa	proline/glycine betaine ABC transporter ATP-binding protein	0.95	0.720	765.6	805.08
SMU_1064c		GntR family transcriptional regulator	1.42	0.002	113.31	79.79
SMU_1065c		GntR family transcriptional regulator	1.94	0.000	99.79	51.5
SMU_1066	guaA	GMP synthase	0.86	0.130	597.86	691.63
SMU_1067c		ABC transporter permease	0.92	0.740	332.81	360.48
SMU_1068c		ABC transporter ATP-binding protein	1.00	1.000	367.35	368.51
SMU_1069c		hypothetical protein	0.69	0.000	201.1	292.52
SMU_1070c		hypothetical protein	0.34	0.000	212.97	627.33
SMU_1071c		hypothetical protein	0.65	0.000	144.35	223.77
SMU_1072c		acetyltransferase	2.21	0.015	17.91	8.09
SMU_1073	fthS	formate--tetrahydrofolate ligase	0.75	0.000	201.61	267.19
SMU_1074		phosphopantothenate--cysteine ligase	0.64	0.000	117.88	183.7
SMU_1075	dfp	phosphopantothenoylcysteine decarboxylase	0.94	0.830	309.3	329.61
SMU_1076		hypothetical protein	1.10	0.160	273.5	248.31
SMU_1077	pgm	phosphoglucomutase	0.93	0.630	592.97	639.1
SMU_1078c		ABC transporter ATP-binding protein	1.04	0.530	283.57	271.44
SMU_1079c		ABC transporter ATP-binding protein	0.94	0.880	211.76	224.34
SMU_1080c		transposon-like protein	1.50	0.000	261.19	174.2
SMU_1081c		hypothetical protein	0.85	0.230	197.1	231.9
SMU_1082	glyA	serine hydroxymethyltransferase	1.19	0.002	402.76	338.07
SMU_1083c		hypothetical protein	1.01	0.880	435.85	431.66
SMU_1084	hemK	N5-glutamine S-adenosyl-L-methionine-dependent methyltransferase	1.17	0.028	247.2	210.44
SMU_1085	rf1	peptide chain release factor 1	1.43	0.000	305.67	213.16
SMU_1086	kitH	thymidine kinase	0.68	0.000	459.89	679.55
SMU_1087		4-oxalocrotonate tautomerase	0.80	0.023	161.88	201.94
SMU_1088	apbE	thiamine biosynthesis lipoprotein	1.03	0.740	197.23	191.23
SMU_1089		hypothetical protein	1.31	0.000	262.22	200.09
SMU_1090		hypothetical protein	1.31	0.000	308.35	235.33
SMU_1091	wapE	cell wall protein, WapE	0.84	0.210	160.04	189.52
SMU_1093		ABC transporter permease	1.26	0.390	29.95	23.76
SMU_1094		ABC transporter ATP-binding protein	1.13	0.770	22.57	19.92
SMU_1095	opuBc	choline ABC transporter, osmoprotectant binding protein	0.94	0.900	160.3	170.63
SMU_1096	opuBa	choline transporter ABC transporter ATP-binding protein	0.97	1.000	174.99	180.26

SMU_1097c		transcriptional regulator	0.73	0.029	59.08	81.01
SMU_1098c		oxidoreductase	0.44	0.000	129.52	295.64
SMU_1100c		permease	0.57	0.000	54.99	96.87
SMU_1102	ascB	6-phospho-beta-glucosidase	1.56	0.000	97.03	62.18
SMU_1104c		phosphoglycerate mutase	1.26	0.010	168.37	133.37
SMU_1105c		phosphoglycerate mutase	1.71	0.000	187.26	109.19
SMU_1106c		phosphoglycerate mutase	1.74	0.000	78.8	45.28
SMU_1107c		hypothetical protein	0.63	0.002	53.74	84.98
SMU_1108c		hypothetical protein	0.76	0.021	83.5	109.91
SMU_1109c		permease	0.74	0.000	261.56	354.68
SMU_1111c		hypothetical protein	1.08	0.550	95.81	88.4
SMU_1112c		hypothetical protein	1.84	0.000	185.11	100.47
SMU_1113	srtA	sortase	0.78	0.002	199.39	255.76
SMU_1114	gyrA	DNA gyrase subunit A	1.00	0.980	343.81	342.33
SMU_1115	ldh	L-lactate dehydrogenase	0.62	0.000	1750.67	2805.41
SMU_1116c		hypothetical protein	0.72	0.004	90.01	125.76
SMU_1117	naoX	NADH oxidase (H2O-forming)	0.92	0.760	203.16	219.94
SMU_1118c		ABC sugar transporter, permease	0.85	0.130	294.93	347.3
SMU_1119c		sugar ABC transporter permease	0.91	0.660	342.49	377.44
SMU_1120		sugar ABC transporter ATP-binding protein	1.10	0.130	335.34	305.26
SMU_1121c		ABC transporter	0.98	0.890	786.95	804.22
SMU_1122	cdd	cytidine deaminase	1.15	0.006	492.93	427.58
SMU_1123	deoC	2-deoxyribose-5-phosphate aldolase	0.73	0.000	393.62	538.65
SMU_1124	pdp	pyrimidine-nucleoside phosphorylase	1.65	0.001	75.92	46.09
SMU_1125c		hypothetical protein	1.17	0.300	70.1	60.06
SMU_1126	coaA	pantothenate kinase	0.48	0.000	26.3	54.69
SMU_1128	ciaH	histidine kinase sensor CiaH	0.68	0.001	90.33	132.49
SMU_1129	ciaR	response regulator CiaR	0.88	0.810	72.54	82.21
SMU_1131c		hypothetical protein	3.95	0.000	76.58	19.4
SMU_1132	pepN	aminopeptidase	0.93	0.550	794.32	854.19
SMU_1133	phoU	phosphate transport system regulatory protein	1.24	0.081	95.26	76.58
SMU_1134c		phosphate transporter ATP-binding protein	1.15	0.089	193.81	168.53
SMU_1135	pstB	phosphate transporter ATP-binding protein	0.96	0.970	147.72	153.43
SMU_1136	pstC	phosphate ABC transporter permease	1.00	1.000	187.23	186.34
SMU_1137	pstC1	phosphate ABC transporter permease	1.50	0.000	235.47	157.18
SMU_1138	pstS	phosphate ABC transporter substrate-binding protein	0.70	0.000	241.26	346.15
SMU_1139c		methylase	1.29	0.025	108.58	84.22
SMU_1140c		hypothetical protein	0.61	0.000	194.3	319.47
SMU_1141c		hypothetical protein	0.65	0.000	193.49	296.29
SMU_1142c		transcriptional regulator Spx	0.66	0.000	448.95	680.82
SMU_1143c		bifunctional riboflavin kinase/FMN adenylyltransferase	1.17	0.040	226.77	194.34
SMU_1144	truB	tRNA pseudouridine synthase B	1.33	0.000	271.43	204.55
SMU_1145c		histidine kinase	0.93	1.000	48.79	52.73
SMU_1146c		response regulator	0.70	0.017	56.58	80.62
SMU_1147c		hypothetical protein	14.72	0.000	22.52	1.53
SMU_1148		transporter, ATP-binding protein; bacteriocin immunity protein	0.79	0.180	45.51	57.8
SMU_1149		transporter, trans-membrane domain bacteriocin immunity protein	1.61	0.014	43.08	26.69
SMU_1150		transporter, trans-membrane domain bacteriocin immunity protein	0.93	1.000	42.57	45.86
SMU_1151c		hypothetical protein	1.02	0.810	292.39	286.43
SMU_1152c		hypothetical protein	1.44	0.000	218.77	152.01
SMU_1153c		hypothetical protein	1.38	0.019	77.33	56.16
SMU_1154c		hypothetical protein	2.13	0.000	110.31	51.9
SMU_1155		hypothetical protein	2.16	0.000	71.55	33.18
SMU_1156c		hypothetical protein	3.07	0.000	62	20.17
SMU_1157c		hypothetical protein	0.99	1.000	10.48	10.54
SMU_1158c		hypothetical protein	1.06	0.700	82.36	77.53
SMU_1159c		hypothetical protein	1.89	0.000	63.41	33.63
SMU_1160c		hypothetical protein	1.93	0.046	17.18	8.9
SMU_1161c		hypothetical protein	1.45	0.170	25.49	17.53
SMU_1163c		ABC transporter ATP-binding protein	1.35	0.013	97.07	71.87
SMU_1164c		ABC transporter ATP-binding protein	1.24	0.130	66.23	53.45
SMU_1165c		transcriptional regulator	3.23	0.000	33.06	10.23
SMU_1166c		ABC transporter permease	0.93	1.000	28.1	30.25
SMU_1167c		ABC transporter ATP-binding protein	0.47	0.005	16.89	35.67
SMU_1168		transcriptional regulator	0.64	0.000	84.61	132.17
SMU_1169c		thioredoxin	1.09	0.370	149.03	136.23
SMU_1170	ccdA	cytochrome C biogenesis protein	1.28	0.043	89.57	69.75
SMU_1171c		hypothetical protein	0.75	0.050	55.66	74.19
SMU_1172c		hypothetical protein	1.26	0.000	380.21	302.22
SMU_1173	cysD	O-acetylhomoserine sulfhydrylase	1.31	0.000	468.43	357.55
SMU_1174	pcrA	ATP-dependent DNA helicase	1.28	0.000	525.66	410.95
SMU_1175		sodium/amino acid (alanine) symporter	0.86	0.300	233.68	271.84
SMU_1176		cation efflux transporter	0.74	0.008	93.34	126.64
SMU_1177c		ABC transporter glutamine binding protein	0.88	0.610	230.12	260.5
SMU_1178c		amino acid ABC transporter ATP-binding protein	1.03	0.640	341.19	331.31
SMU_1179c		amino acid ABC transporter permease	1.03	0.640	294.91	285.01
SMU_1180	phnA	alkylphosphonate uptake protein	0.84	0.390	68.86	82.04
SMU_1182	mtlD	mannitol-1-phosphate 5-dehydrogenase	0.80	0.180	59.3	74.05
SMU_1183	mtlA2	PTS system mannitol-specific transporter subunitIIA	1.13	0.370	76.69	67.57
SMU_1184c		transcriptional regulator, antiterminator	0.60	0.094	13.84	23.17
SMU_1185	mtlA1	PTS system mannitol-specific transporter subunitIIBC	1.35	0.170	38.41	28.45
SMU_1187	glmS	glucosamine--fructose-6-phosphate aminotransferase	1.13	0.042	376.94	334.88
SMU_1188	lepB	signal peptidase	0.49	0.000	155.95	319.69
SMU_1189c		hypothetical protein	0.65	0.000	523.7	802.74
SMU_1190	pykF	pyruvate kinase	0.97	0.730	2079.34	2136.39
SMU_1191	pfk	6-phosphofructokinase	1.18	0.000	2851.99	2412.88
SMU_1192	dnaE	DNA polymerase III DnaE	1.12	0.160	191.41	170.28
SMU_1193		transcriptional regulator	0.68	0.000	132.48	193.92
SMU_1194		ABC transporter ATP-binding protein	1.04	0.650	212.77	204.78
SMU_1195		permease	1.03	0.760	159.88	154.61
SMU_1196c		hypothetical protein	0.71	0.000	412.58	578.29

SMU_1197		hypothetical protein	1.08	0.430	140.06	129.55
SMU_1200	rs1	30S ribosomal protein S1	0.78	0.000	4177.98	5372.54
SMU_1201c		hypothetical protein	1.57	0.000	115.82	73.78
SMU_1203	ilvE	branched-chain amino acid aminotransferase	0.53	0.000	459.15	865.34
SMU_1204	parC	DNA topoisomerase IV subunit A	1.30	0.000	366.71	281.44
SMU_1205c		hypothetical protein	1.46	0.005	81.56	55.86
SMU_1206c		hypothetical protein	1.82	0.000	70.63	38.76
SMU_1207	fic	hypothetical protein	1.50	0.000	514.85	343.72
SMU_1208c		hypothetical protein	1.48	0.000	401.57	271.01
SMU_1209c		hypothetical protein	1.80	0.000	101.36	56.34
SMU_1210	parE	DNA topoisomerase IV subunit B	1.68	0.000	290.05	172.42
SMU_1211		glycerol-3-phosphate acyltransferase PlsY	0.79	0.011	149.63	190.16
SMU_1213c		5'-nucleotidase	1.23	0.002	284.43	230.61
SMU_1214	pyrC	dihydroorotase	1.16	0.038	239.22	205.51
SMU_1215	ung	uracil-DNA glycosylase	1.48	0.000	345.09	233.22
SMU_1216c		amino acid ABC transporter permease	0.61	0.000	139.23	226.48
SMU_1217c		ABC transporter amino acid binding protein	1.75	0.000	121.78	69.47
SMU_1218	nylA	amidase	1.51	0.000	164.85	109.25
SMU_1219c		hypothetical protein	2.15	0.000	114.43	53.25
SMU_1220c		hypothetical protein	1.27	0.000	348.18	273.21
SMU_1221	pyrE	orotate phosphoribosyltransferase	1.32	0.000	319.28	241.65
SMU_1222	pyrF	orotidine 5'-phosphate decarboxylase	1.26	0.000	436.83	345.33
SMU_1223	pyrDB	dihydroorotate dehydrogenase 1B	2.00	0.000	161.86	80.78
SMU_1224	pyrK	dihydroorotate dehydrogenase electron transfer subunit	1.85	0.000	120.05	64.82
SMU_1225	cpsY	transcriptional regulator	0.55	0.000	195.06	352.81
SMU_1226c		hypothetical protein	0.81	0.067	129.62	160.06
SMU_1227	deoD	purine nucleoside phosphorylase	1.08	0.230	303.23	280.52
SMU_1228c		glutamine amidotransferase	1.25	0.000	346.88	278.16
SMU_1229	punA	purine nucleoside phosphorylase	0.92	0.790	217.15	235.73
SMU_1230c		hypothetical protein	1.23	0.002	299.2	243.4
SMU_1231c		hypothetical protein	0.88	0.290	664.55	755.87
SMU_1232c		hypothetical protein	1.17	0.021	299.54	256.89
SMU_1233	deoB	phosphopentomutase	1.33	0.000	374.55	282.57
SMU_1234	rpiA	ribose-5-phosphate isomerase A	0.93	0.830	146.74	158.44
SMU_1235	thdF	tRNA modification GTPase TrmE	1.07	0.410	237.92	222.78
SMU_1236c		hypothetical protein	0.96	1.000	40.04	41.76
SMU_1237c		hypothetical protein	0.09	0.000	29.64	322.67
SMU_1238c		hypothetical protein	1.04	0.450	575.77	556.06
SMU_1239	pepV	dipeptidase PepV	1.14	0.009	540.13	474.78
SMU_1240c		nitroreductase	0.83	0.072	248.49	298.01
SMU_1241	uvrC	excinuclease ABC subunit C	1.13	0.110	215.8	191.3
SMU_1243		low temperature requirement A protein	1.43	0.003	100.37	70.41
SMU_1245c		hypothetical protein	1.26	0.000	449.79	356.98
SMU_1246c		transcriptional regulator	0.71	0.001	103.76	146.59
SMU_1247	eno	enolase	0.81	0.000	4938.91	6089.94
SMU_1249c		hypothetical protein	0.65	0.000	108.19	167.1
SMU_1250c		hypothetical protein	0.47	0.000	38.9	82.21
SMU_1251		hypothetical protein	0.38	0.000	29.96	78.83
SMU_1252	grk	glycerate kinase	1.62	0.000	97.87	60.46
SMU_1253c		hypothetical protein	4.91	0.000	39.38	8.02
SMU_1254		hypothetical protein	0.73	0.005	96.5	131.69
SMU_1255c		hypothetical protein	0.60	0.000	144.15	240.99
SMU_1256c		hypothetical protein	0.90	0.650	280.69	311.3
SMU_1257c		hypothetical protein	0.51	0.000	303.22	599.4
SMU_1258c		restriction endonuclease	0.63	0.240	12.22	19.32
SMU_1259		restriction endonuclease		0.000	8.66	0
SMU_1260c		hypothetical protein	0.96	1.000	41.08	42.68
SMU_1261c		phosphoribosyl-ATP pyrophosphohydrolase	4.48	0.000	123.28	27.54
SMU_1262c		hypothetical protein	1.64	0.440	7.28	4.45
SMU_1263	hisI	phosphoribosyl-ATP pyrophosphatase / phosphoribosyl-AMP cyclohydrol	1.72	0.014	34.75	20.26
SMU_1264	hisF	imidazole glycerol phosphate synthase subunit HisF	1.42	0.022	64.31	45.17
SMU_1265	hisA	1-(5-phosphoribosyl)-5-[(5- phosphoribosylamino)methylideneamino] imic	0.94	1.000	63.24	66.99
SMU_1266	hisH	imidazole glycerol phosphate synthase subunit HisH	1.26	0.120	66.34	52.59
SMU_1267c		hypothetical protein	1.38	0.055	53.15	38.41
SMU_1268	hisB	imidazoleglycerol-phosphate dehydratase	1.99	0.000	69.75	35.02
SMU_1269	serB	phosphoserine phosphatase	1.27	0.150	57.87	45.45
SMU_1270	hisD	bifunctional histidinal dehydrogenase/ histidinol dehydrogenase	1.67	0.002	55.06	33.02
SMU_1271	hisG	ATP phosphoribosyltransferase	1.31	0.180	42.21	32.28
SMU_1272	hisZ	histidyl-tRNA synthetase	1.23	0.320	39.39	31.91
SMU_1273	hisC	histidinol-phosphate aminotransferase	1.27	0.320	33.71	26.56
SMU_1276c		septation ring formation regulator EzrA	1.00	1.000	489.8	489.28
SMU_1277	gyrB	DNA gyrase subunit B	1.02	0.750	466.56	458.46
SMU_1278c		hypothetical protein	0.91	0.670	254.18	278.97
SMU_1279c		cell shape determining protein	0.67	0.000	192.37	287.35
SMU_1280c		hypothetical protein	3.21	0.002	14.72	4.58
SMU_1282		transcriptional regulator	1.67	0.028	33.44	20.07
SMU_1284c		hypothetical protein	0.75	0.065	54.62	72.87
SMU_1286c		permease	0.67	0.016	45.12	67.1
SMU_1287		transcriptional regulator	0.48	0.000	28.58	59.19
SMU_1288	rl19	50S ribosomal protein L19	0.46	0.000	5224.54	11452
SMU_1289c		chloride channel permease	1.13	0.360	86.88	76.91
SMU_1290c		chloride channel permease	1.00	1.000	82.59	82.27
SMU_1291c		hypothetical protein	0.85	0.570	57.71	67.75
SMU_1292c		hypothetical protein	0.92	0.780	225.06	243.85
SMU_1293c		hypothetical protein	0.85	0.160	314.51	368.1
SMU_1294	flaW	flavodoxin	174.25	0.000	55.76	0.32
SMU_1295	add	adenosine deaminase	0.72	0.007	86.88	119.93
SMU_1296		S-transferase	1.18	0.009	342.47	291.38
SMU_1297		hypothetical protein	0.57	0.000	284.1	497.76
SMU_1298	rl31	50S ribosomal protein L31 type B	0.50	0.000	1366.11	2748.43
SMU_1299c		acetate kinase	0.78	0.002	180.57	231.43

SMU_1300c		hypothetical protein	0.61	0.000	258.77	421.52
SMU_1301c		methyltransferase	1.26	0.007	186.1	147.18
SMU_1302	adcA	surface adhesin	1.09	0.430	115.31	105.52
SMU_1303c		dipeptidase	1.11	0.096	341.64	308.93
SMU_1304c		hypothetical protein	0.93	0.800	280.05	301.1
SMU_1305c		hypothetical protein	0.96	0.870	417.89	436.44
SMU_1306c		glmZ(sRNA)-inactivating NTPase	1.78	0.000	236.17	132.7
SMU_1307c		hypothetical protein	0.90	0.790	164.67	182.78
SMU_1308	aldR	translation initiation inhibitor; aldR regulator-like protein	1.31	0.000	272.46	208.15
SMU_1309c		glycerol dehydrogenase	1.06	0.500	206.94	195.44
SMU_1310		hypothetical protein	1.86	0.000	74.96	40.21
SMU_1311	asnS	asparaginyl-tRNA synthetase	0.90	0.480	623.66	689.54
SMU_1312	aspB	aspartate aminotransferase	0.92	0.750	262.67	286.97
SMU_1313c		bifunctional ATP-dependent DNA helicase/DNA polymerase III subunit ep	1.02	0.900	152.48	149.97
SMU_1314		hypothetical protein	0.63	0.000	514.37	815
SMU_1315c		ATP-binding protein	1.10	0.490	96.71	88.11
SMU_1316c		hypothetical protein	1.82	0.000	65.56	35.96
SMU_1317c		hypothetical protein	1.42	0.014	71.08	49.92
SMU_1319c		hypothetical protein	0.85	0.280	144.38	170.34
SMU_1321c		hypothetical protein	0.76	0.011	103.62	136.37
SMU_1322	budC	acetoin reductase	0.67	0.000	551.63	825.04
SMU_1323		hydrolase	0.56	0.000	294.16	529.05
SMU_1324	ftsX	cell-division protein FtsX	0.89	0.450	492.62	553.17
SMU_1325	ftsE	ABC transporter ATP-binding protein	1.06	0.140	711.81	669.93
SMU_1326	rf2	peptide chain release factor 2	1.17	0.000	922.72	785.31
SMU_1327c		4Fe-4S ferredoxin	0.72	0.004	89.47	124.78
SMU_1329c		transposase	0.63	0.011	38.26	60.34
SMU_1330c		transposase	0.98	1.000	30.82	31.29
SMU_1331c		transposase	1.42	0.067	42.64	29.97
SMU_1332c		transposase	0.82	0.650	21.67	26.41
SMU_1334	sfp	phosphopantetheinyl transferase	1.02	0.850	199.79	196.28
SMU_1335c		enoyl-ACP reductase	1.63	0.000	221.53	135.92
SMU_1336	pksD	hypothetical protein	1.13	0.240	133.14	118.19
SMU_1337c		alpha/beta hydrolase	1.77	0.000	99.33	56.15
SMU_1338c		permease	1.15	0.160	132.38	114.81
SMU_1339	bacD	bacitracin synthetase	1.10	0.450	106.47	97.2
SMU_1340	bacA2	surfactin synthetase	0.96	0.960	86.87	90.2
SMU_1341c		gramicidin S synthetase	0.89	0.980	46.62	52.4
SMU_1342	bacA1	bacitracin synthetase 1; BacA	0.87	0.950	30.34	34.86
SMU_1343c		polyketide synthase	0.79	0.150	54.18	68.84
SMU_1344c		malonyl CoA-ACP transacylase	0.81	0.540	26.67	32.94
SMU_1345c		peptide synthetase	0.73	0.033	54.01	74.21
SMU_1346	bacT	thioesterase	0.80	0.180	60.06	75.29
SMU_1347c		permease	0.90	0.830	100.08	110.82
SMU_1348c		ABC transporter ATP-binding protein	0.81	0.049	155.85	191.73
SMU_1349		hypothetical protein	0.51	0.000	101.81	199.59
SMU_1351		transposase fragment	0.62	0.001	53.25	85.6
SMU_1352		transposase	0.57	0.001	37.75	66.75
SMU_1353		transposase	1.39	0.100	42.99	31.01
SMU_1354c		transposase fragment	0.63	0.000	69.55	110.66
SMU_1355c		transposase fregment	0.78	0.051	81.14	104.05
SMU_1356c		transposase fragment	1.52	0.003	74.48	48.89
SMU_1357		transposase fragment	1.21	0.016	218.48	180.7
SMU_1358		transposase fragment	1.31	0.000	563.57	431.02
SMU_1359		hypothetical protein	1.24	0.084	95.15	76.91
SMU_1360c		hypothetical protein	0.60	0.000	119.21	199.63
SMU_1361c		TetR family transcriptional regulator	0.62	0.000	152.9	245.69
SMU_1363c		transposase	1.05	0.490	308.1	294.65
SMU_1365c		permease	0.90	0.780	106.66	118.38
SMU_1366c		ABC transporter ATP-binding protein	0.74	0.000	154.83	210.38
SMU_1367c		hypothetical protein	1.33	0.000	230.52	173.11
SMU_1368		hypothetical protein	0.43	0.000	30.81	71.14
SMU_1369		hypothetical protein		0.000	40.93	0
SMU_1370c		transposase, IS150-like	0.92	1.000	27.11	29.47
SMU_1372c		hypothetical protein	0.92	1.000	55.85	60.89
SMU_1373c		hypothetical protein	1.06	0.790	65.77	62.2
SMU_1374		hypothetical protein	1.61	0.000	219.41	136.7
SMU_1375c		hypothetical protein	2.63	0.000	85.15	32.4
SMU_1377c		hypothetical protein	0.64	0.000	521.43	809.33
SMU_1378		hypothetical protein	0.70	0.120	29.22	41.59
SMU_1379		hypothetical protein	1.11	0.850	18.19	16.45
SMU_1381	leuD	3-isopropylmalate dehydratase small subunit	1.75	0.000	260.99	148.79
SMU_1382	leuC	3-isopropylmalate dehydratase large subunit	1.73	0.000	540.31	312.08
SMU_1383	leuB	3-isopropylmalate dehydrogenase	1.26	0.000	555.63	440.04
SMU_1384	leuA	2-isopropylmalate synthase	1.50	0.000	410.98	273.57
SMU_1386	urk	uridine/cytidine kinase	0.68	0.000	138.6	202.36
SMU_1387		oxidoreductase	0.64	0.000	104.72	163.02
SMU_1388		RNA helicase	0.83	0.190	132.52	158.97
SMU_1389	pckA	hypothetical protein	0.95	0.840	238.01	251.27
SMU_1390		hypothetical protein	0.60	0.000	235.78	390.02
SMU_1391c		hypothetical protein	0.85	0.280	168.63	198.36
SMU_1392c		acetyltransferase	1.05	0.700	104.11	98.74
SMU_1393c		hypothetical protein	0.67	0.000	107.52	161.52
SMU_1394	lepA	GTP-binding protein LepA	0.82	0.037	238.32	289.14
SMU_1395c		hypothetical protein		0.000	8.24	0
SMU_1396	gbpC	glucan-binding protein GbpC	1.32	0.000	394.64	297.88
SMU_1397c		hypothetical protein	0.64	0.000	376.6	585.72
SMU_1398		transcriptional regulator	0.76	0.046	66.06	87.2
SMU_1400c		hypothetical protein	0.69	0.000	523.7	764.1
SMU_1402c		hypothetical protein	0.79	0.160	49.22	62.33
SMU_1403c		hypothetical protein	1.52	0.000	214.77	140.99

SMU_1404c		hypothetical protein	1.57	0.000	123.22	78.44
SMU_1405c		hypothetical protein	1.15	0.550	40.04	34.67
SMU_1406c		hypothetical protein	0.77	0.016	100.4	130.3
SMU_1407c		transposase, ISSmu1	1.55	0.086	26.88	17.34
SMU_1408c		hypothetical protein	1.65	0.110	18.11	10.97
SMU_1409c		transcriptional regulator	0.39	0.077	4.9	12.47
SMU_1410		reductase	2.20	0.001	33	14.98
SMU_1411		hypothetical protein	1.73	0.012	33.78	19.48
SMU_1412c		ABC transporter membrane protein subunit and ATP-binding protein	0.93	0.830	189.9	203.78
SMU_1414c		hypothetical protein	1.09	0.610	66.35	60.79
SMU_1415c		phosphatase	1.19	0.043	188.27	158.45
SMU_1416c		mutator protein MutT	1.28	0.007	165.47	129.38
SMU_1417c		oleoyl-acyl carrier protein thioesterase	1.82	0.000	110.2	60.7
SMU_1418	hemN	coproporphyrinogen III oxidase	0.77	0.005	134.48	175.64
SMU_1419		transcriptional regulator	0.87	0.400	261.39	300.77
SMU_1420		oxidoreductase	1.53	0.000	269.43	176.66
SMU_1421	pdhC	branched-chain alpha-keto acid dehydrogenase E2 subunit	1.13	0.580	39.38	34.95
SMU_1422	pdhB	pyruvate dehydrogenase E1 component subunit beta	1.68	0.014	38.8	23.16
SMU_1423	pdhA	pyruvate dehydrogenase, TPP-dependent E1 component alpha-subunit	5.29	0.000	36.42	6.89
SMU_1424	pdhD	dihydrolipoamide dehydrogenase	1.21	0.420	38.49	31.9
SMU_1425	clpB	Clp proteinase, ATP-binding subunit ClpB	1.69	0.073	21.63	12.78
SMU_1426c		phosphoglucosamine mutase	1.00	1.000	723.52	723.53
SMU_1427c		hypothetical protein	0.82	0.049	217.34	263.75
SMU_1428c		hypothetical protein	0.70	0.000	282.75	403.21
SMU_1429	murC2	UDP-N-acetylmuramyl tripeptide synthetase MurC	0.62	0.000	265.74	425.36
SMU_1430	cobQ	cobyric acid synthase CobQ	0.74	0.000	281.19	380.49
SMU_1431c		ABC transporter ATP-binding protein	1.02	0.830	226.33	221.9
SMU_1432c		endoglucanase	0.85	0.083	338	399.77
SMU_1434c		glycosyltransferase	0.97	0.930	306.76	317.33
SMU_1435c		hypothetical protein	2.02	0.000	87.04	43.03
SMU_1436c		hypothetical protein	1.23	0.014	192.95	157.07
SMU_1437	epsC	UDP-N-acetylglucosamine 2-epimerase	0.94	0.770	370.18	392.05
SMU_1438c		Zn-dependent protease	0.52	0.000	252.93	485.26
SMU_1442c		hypothetical protein	0.84	0.089	249.68	298.22
SMU_1443c		tributyrin esterase	0.83	0.030	317.64	382.47
SMU_1444c		hypothetical protein	1.02	0.820	401.87	395.31
SMU_1445c		ABC transporter ATP-binding protein	1.06	0.390	298.75	281.94
SMU_1446c		ABC transporter permease	1.05	0.670	119.26	113.18
SMU_1447c		hypothetical protein	0.90	0.650	236.11	262.41
SMU_1449		fibronectin/fibrinogen-binding protein	2.28	0.000	105.55	46.22
SMU_1450		amino acid permease	0.73	0.000	201.2	277.43
SMU_1451	aldB	alpha-acetolactate decarboxylase	0.90	0.660	252.95	279.6
SMU_1452	alsS	acetolactate synthase	0.84	0.018	453.14	541.6
SMU_1453c		hypothetical protein	0.96	1.000	113.56	118.28
SMU_1454c		hypothetical protein	1.12	0.380	88.28	78.8
SMU_1455	mutX	8-oxo-dGTP diphosphatase	1.13	0.300	102.87	90.71
SMU_1456c		hypothetical protein	1.25	0.460	24.85	19.85
SMU_1457	rmlB	dTDP-glucose-4,6-dehydratase	1.06	0.140	875.16	828.41
SMU_1459c		hypothetical protein	1.58	0.000	1511.36	956.71
SMU_1460	rmlC	dTDP-4-keto-L-rhamnose reductase	0.97	0.850	653.84	675.06
SMU_1461	rmlA	glucose-1-phosphate thymidylyltransferase	0.99	0.960	584.74	592.67
SMU_1462c		oxidoreductase	1.97	0.000	68.66	34.8
SMU_1463c		hypothetical protein	1.40	0.008	92.1	65.82
SMU_1464c		hypothetical protein	1.43	0.000	136.43	95.26
SMU_1465c		replication protein DnaD-like	1.16	0.019	325.59	281.09
SMU_1466	metA	homoserine O-succinyltransferase	0.98	1.000	261.19	267.09
SMU_1467	apt	adenine phosphoribosyltransferase	0.96	0.830	584.76	606.95
SMU_1470c		hypothetical protein	0.75	0.077	44.67	59.79
SMU_1471c		hypothetical protein	0.70	0.073	30.37	43.63
SMU_1472	recJ	single-strand DNA-specific exonuclease RecJ	1.18	0.041	210.19	178.28
SMU_1473c		oxidoreductase	0.75	0.000	199.2	265.97
SMU_1474c		ribonuclease Z	1.26	0.031	122.82	97.76
SMU_1475c		hypothetical protein	0.94	0.930	147.89	156.6
SMU_1476c		GTP-binding protein	0.67	0.000	534.85	796
SMU_1477	miaA	tRNA delta(2)-isopentenylpyrophosphate transferase	0.56	0.000	147.24	261.74
SMU_1479		hypothetical protein	0.76	0.001	175.11	231.51
SMU_1480		hypothetical protein	0.92	0.950	92.57	100.83
SMU_1482c		hypothetical protein	1.25	0.022	139.95	111.64
SMU_1483c		hypothetical protein	1.24	0.005	233.11	188.14
SMU_1484c		hypothetical protein	1.64	0.000	142.28	86.55
SMU_1485c		endonuclease	1.10	0.710	41.23	37.41
SMU_1486c		hypothetical protein	1.23	0.048	128.92	105.11
SMU_1487		hypothetical protein	0.86	0.520	89.93	104.54
SMU_1488c		hypothetical protein	1.24	0.070	91.19	73.25
SMU_1489	lacX	hypothetical protein	3.07	0.000	78.32	25.54
SMU_1490	lacG	6-phospho-beta-galactosidase	4.31	0.000	153.56	35.6
SMU_1491	lacE	PTS system lactose-specific transporter subunit IIBC	2.74	0.000	74.98	27.34
SMU_1492	lacF	PTS system lactose-specific transporter subunit IIA	7.79	0.000	52.76	6.77
SMU_1493	lacD	tagatose 1,6-diphosphate aldolase	3.25	0.000	53.47	16.44
SMU_1494	lacC	tagatose-6-phosphate kinase	4.12	0.000	60.33	14.64
SMU_1495	lacB	galactose-6-phosphate isomerase subunit LacB	2.59	0.006	17.11	6.61
SMU_1496	lacA	galactose-6-phosphate isomerase subunit LacA	11.78	0.000	31.21	2.65
SMU_1498	lacR	lactose repressor	1.96	0.003	33.92	17.32
SMU_1499	rexA	exonuclease RexA	1.16	0.150	129.54	111.66
SMU_1500	rexB	exonuclease RexB	1.15	0.250	106.86	93.19
SMU_1502c		hypothetical protein	0.58	0.000	154.77	268.37
SMU_1504c		hypothetical protein	0.53	0.002	27.79	52.21
SMU_1505c		hypothetical protein	0.44	0.001	19.66	45.06
SMU_1506c		hypothetical protein	0.84	0.930	15.67	18.66
SMU_1507c		hypothetical protein	0.19	0.002	3.28	17.57
SMU_1508c		coenzyme PQQ synthesis protein	1.91	0.004	32.13	16.83

SMU_1509	rgg	transcriptional regulator	0.88	0.990	30.76	34.82
SMU_1510	syfB	phenylalanyl-tRNA synthetase subunit beta	0.85	0.230	181.01	213.59
SMU_1511c		acetyltransferase	1.12	0.360	98.56	87.77
SMU_1512	syfA	phenylalanyl-tRNA synthetase subunit alpha	0.86	0.480	152.84	177.16
SMU_1513	smc	chromosome segregation ATPase	1.16	0.040	254.29	219.47
SMU_1514	rnc	ribonuclease III	1.17	0.150	111.97	95.67
SMU_1515	covX	hypothetical protein	0.84	0.046	412.99	490.02
SMU_1516	covS	histidine kinase CovS	1.03	0.610	359.78	348.4
SMU_1517	covR	response regulator CovR	0.92	0.590	492.54	535.76
SMU_1519	glnQ	amino acid ABC transporter ATP-binding protein	0.61	0.000	685.73	1125.35
SMU_1520		ABC transporter glutamine binding protein	1.09	0.063	573.51	525.89
SMU_1521		amino acid ABC transporter permease	0.93	0.650	582.65	623.62
SMU_1522	glnP	amino acid ABC transporter integral membrane protein	0.74	0.000	326.44	438.86
SMU_1523	endA	membrane nuclease EndA	0.91	0.790	175.68	193.75
SMU_1524c		hypothetical protein	0.63	0.000	165.24	264.24
SMU_1525	murA	UDP-N-acetylglucosamine 1-carboxyvinyltransferase	0.98	0.970	466.44	475.61
SMU_1526c		hypothetical protein	1.46	0.095	31.66	21.68
SMU_1527	atpA	ATP synthase F0F1 subunit epsilon	1.04	0.160	1492.51	1435.43
SMU_1528	atpB	ATP synthase F0F1 subunit beta	1.16	0.000	2078.68	1799.6
SMU_1529	atpC	ATP synthase F0F1 subunit gamma	1.17	0.000	984.23	838.15
SMU_1530	atpD	ATP synthase F0F1 subunit alpha	1.25	0.000	1264.47	1008.72
SMU_1531	atpE	ATP synthase F0F1 subunit delta	1.11	0.052	471.29	426.05
SMU_1532	atpF	ATP synthase F0F1 subunit B	1.04	0.160	1210.38	1158.53
SMU_1533	atpG	ATP synthase F0F1 subunit A	0.93	0.620	603.64	649.75
SMU_1534	atpH	ATP synthase F0F1 subunit C	0.80	0.001	293.83	368.95
SMU_1535	phsG	glycogen phosphorylase	1.42	0.000	188.22	132.55
SMU_1536	glgA	glycogen synthase	0.97	1.000	134.16	137.9
SMU_1537	glgD	glycogen biosynthesis protein GlgD	0.70	0.037	41.97	59.6
SMU_1538	glgC	glucose-1-phosphate adenylyltransferase	1.09	0.520	82.22	75.12
SMU_1539	glgB	glycogen branching protein	1.10	0.610	47.9	43.43
SMU_1541	pulA	pullulanase	1.17	0.015	311.56	266.07
SMU_1542c		lipid kinase	1.26	0.000	356.86	284.15
SMU_1543	dnlJ	NAD-dependent DNA ligase LigA	0.86	0.180	344.83	401.86
SMU_1545c		hypothetical protein	0.73	0.002	113.55	156.42
SMU_1546		hypothetical protein	0.76	0.043	68.98	90.21
SMU_1547c		response regulator	0.93	0.800	212.74	229.27
SMU_1548c		histidine kinase	0.92	0.770	192.83	208.57
SMU_1550c		hypothetical protein	2.87	0.000	51.46	17.93
SMU_1551c		ABC transporter ATP-binding protein	2.23	0.001	28.1	12.59
SMU_1552c		hypothetical protein	1.41	0.270	19.66	13.99
SMU_1553c		hypothetical protein		0.000	13.51	0
SMU_1554c		hypothetical protein		0.069	2.58	0
SMU_1555c		hypothetical protein	1.25	0.007	198.74	158.44
SMU_1556	ampM	methionine aminopeptidase	1.62	0.000	708.76	438.24
SMU_1557c		hypothetical protein	2.12	0.000	152.65	72.03
SMU_1558c		acetyltransferase	1.30	0.150	46.81	36.14
SMU_1560		hypothetical protein	0.56	0.000	41.23	74.23
SMU_1561	trkB	potassium uptake system protein TrkB	1.22	0.029	175.15	144
SMU_1562	trk	potassium uptake protein TrkA	1.04	0.680	165.6	158.59
SMU_1563	pacL	cation-transporting P-type ATPase PacL	1.12	0.063	363.7	325.47
SMU_1564	glgP	glycogen phosphorylase	1.38	0.001	149.9	108.72
SMU_1565	malQ	4-alpha-glucanotransferase	1.01	0.960	176.61	175.21
SMU_1566	malR	maltose operon transcriptional repressor	0.66	0.000	215.79	325.58
SMU_1568	malX	maltose ABC transporter substrate-binding protein	1.27	0.018	137.38	107.85
SMU_1569	malF	maltose ABC transporter permease	0.92	0.930	44.18	47.95
SMU_1570	malG	maltose ABC transporter permease	1.41	0.033	60.66	43.01
SMU_1571		MsmK-like ABC transporter ATP-binding protein	1.73	0.000	103.16	59.47
SMU_1572	murZ	UDP-N-acetylglucosamine 1-carboxyvinyltransferase	0.86	0.016	1158.28	1349.41
SMU_1573	metK	S-adenosylmethionine synthetase	0.79	0.000	597.73	757.88
SMU_1574c		hypothetical protein	0.98	1.000	72.17	73.36
SMU_1575c		hypothetical protein	2.25	0.009	17.95	7.99
SMU_1576c		hypothetical protein	1.55	0.033	37.45	24.16
SMU_1577c		hypothetical protein	2.01	0.035	18.59	9.25
SMU_1578	birA	bifunctional biotin--[acetyl-CoA-carboxylase] synthetase/biotin operon rep	0.70	0.000	234.96	335.03
SMU_1579		hypothetical protein	0.68	0.000	263.68	386.5
SMU_1581	dnaX	DNA polymerase III subunits gamma and tau	0.83	0.005	456.92	553.77
SMU_1582c		hypothetical protein	0.86	0.430	151.76	176.28
SMU_1584c		hypothetical protein	2.75	0.020	10.57	3.85
SMU_1585c		transcriptional regulator	0.33	0.000	39.48	119.79
SMU_1586	syt1	threonyl-tRNA synthetase	0.96	0.900	302.54	313.82
SMU_1587c		hypothetical protein	0.70	0.000	266.89	380.53
SMU_1588c		hexosyltransferase	0.86	0.096	502.89	586.28
SMU_1589c		hexosyltransferase	0.77	0.000	504.47	655.01
SMU_1590	amyA	alpha-amylase	0.82	0.110	132.31	160.69
SMU_1591	ccpA	catabolite control protein CcpA	0.61	0.000	846.16	1394.13
SMU_1592	pepQ	dipeptidase PepQ	0.80	0.059	119.67	149.13
SMU_1593c		CDP-diglyceride synthetase	0.89	0.670	241.41	270.01
SMU_1595	cah	carbonic anhydrase	0.77	0.056	66.83	87.11
SMU_1596	ptcC	cellobiose phosphotransferase system IIC component	0.62	0.380	7.47	12.14
SMU_1597c		hypothetical protein	0.15	0.730	0.49	3.29
SMU_1598	ptcA	cellobiose phosphotransferase system IIA component	1.00	1.000	5.48	5.47
SMU_1599	celR	transcriptional regulator	0.93	1.000	3.71	4
SMU_1600	ptcB	PTS system cellobiose transporter subunit IIB		1.000	0.62	0
SMU_1601	bgl	6-phospho-beta-glucosidase	0.53	0.004	23.65	44.84
SMU_1602		NAD(P)H-flavin oxidoreductase	0.77	0.000	572.56	738.97
SMU_1603	lguL	lactoylglutathione lyase	1.00	1.000	539.65	539.8
SMU_1604c		hypothetical protein	1.26	0.630	9.74	7.76
SMU_1605		MDR permease	0.77	0.005	138.26	180.08
SMU_1606	smpB	SsrA-binding protein	0.92	0.860	176.83	191.23
SMU_1607	vacB	exoribonuclease R	0.92	0.810	169.24	184.78
SMU_1609c		preprotein translocase subunit SecG	0.44	0.000	106.13	239.54

SMU_1610	rl33	50S ribosomal protein L33	1.10	0.410	121.34	110.34
SMU_1611c		permease	0.81	0.009	239.59	297.09
SMU_1612c		hypothetical protein	1.28	0.000	342.36	266.88
SMU_1613c		dephospho-CoA kinase	1.07	0.400	235	219.97
SMU_1614	fpg	formamidopyrimidine/5-formyluracil/ 5-hydroxymethyluracil DNA glycosyla	1.06	0.590	154.2	145.92
SMU_1615c		hypothetical protein	1.14	0.057	280.05	246.07
SMU_1616c		hypothetical protein	1.18	0.016	275.58	233.21
SMU_1617	era	GTPase Era	1.03	0.750	177.76	172
SMU_1618	dagK	diacylglycerol kinase	0.61	0.000	97.48	159.47
SMU_1619c		metal-binding heat shock protein	0.99	1.000	260.35	263.87
SMU_1620	phoH	phosphate starvation-induced protein PhoH	0.81	0.000	547.88	673.4
SMU_1621c		hypothetical protein	1.07	0.490	147.5	137.67
SMU_1622	pmsR	methionine sulfoxide reductase A	0.79	0.002	234.68	297.11
SMU_1623c		hypothetical protein	0.87	0.330	361.76	414.78
SMU_1624	rrf1	ribosome recycling factor	1.24	0.000	501.88	403.89
SMU_1625	pyrH	uridylate kinase	0.99	0.990	1005.54	1014.09
SMU_1626	rl1	50S ribosomal protein L1	0.69	0.000	4060.84	5846.93
SMU_1627	rl11	50S ribosomal protein L11	0.67	0.000	4929.52	7306.77
SMU_1628		hypothetical protein	0.51	0.000	38.33	75.56
SMU_1629c		cell division DNA segregation ATPase	0.96	0.890	395.38	410.78
SMU_1631		peptidyl-prolyl cis-trans isomerase	4.19	0.000	27.64	6.59
SMU_1632	pfs	5'-methylthioadenosine/S-adenosylhomocysteine nucleosidase	0.99	1.000	433.78	437.48
SMU_1633c		hypothetical protein	1.46	0.000	281.09	192.79
SMU_1634c		hypothetical protein	1.10	0.100	384.81	350.81
SMU_1635	glmU	UDP-N-acetylglucosamine pyrophosphorylase	1.07	0.250	403.36	378.08
SMU_1636c		hypothetical protein	0.94	0.680	581.58	617.53
SMU_1637c		hypothetical protein	1.23	0.000	781.52	637.86
SMU_1638c		hypothetical protein	0.90	0.590	391.26	432.99
SMU_1639	metS	methionyl-tRNA synthetase	1.10	0.008	948.41	861.26
SMU_1641c		hypothetical protein	0.42	0.000	1009.72	2430.81
SMU_1642c		hypothetical protein	1.08	0.690	65.27	60.69
SMU_1643c		hypothetical protein	1.95	0.009	26.38	13.55
SMU_1644c		hypothetical protein	0.66	0.000	95.05	143.7
SMU_1645		tellurite resistance protein TehB	0.55	0.000	375.97	678.5
SMU_1646c		hemolysis inducing protein	0.69	0.000	128.98	187.93
SMU_1647c		transcriptional regulator	0.48	0.000	119.06	246.83
SMU_1648c		hypothetical protein	0.87	0.830	63.31	72.88
SMU_1649	exoA	exodeoxyribonuclease III	0.55	0.000	747.1	1348.06
SMU_1650	end3	endonuclease III (DNA repair)	0.82	0.220	92.63	112.41
SMU_1651		arsenate reductase	0.62	0.000	288.22	463.82
SMU_1652	ogt	methylated DNA-protein cysteine methyltransferase	0.85	0.170	272.98	320.2
SMU_1653	serA	D-3-phosphoglycerate dehydrogenase	0.97	0.910	345.99	355.34
SMU_1654c		acetyltransferase	0.91	0.580	454.35	501.45
SMU_1655c		hypothetical protein	1.02	0.640	713.84	700.3
SMU_1656	serC	3-phosphoserine/phosphohydroxythreonine aminotransferase	0.78	0.000	376.03	484.69
SMU_1657c		nitrogen regulatory protein PII	0.58	0.000	50.89	87.78
SMU_1658	nrgA	ammonium transporter	0.56	0.002	36.23	64.57
SMU_1659c		hypothetical protein	0.90	0.720	215.24	238.78
SMU_1660c		DNA replication intiation control protein YabA	1.03	0.660	346.43	336.26
SMU_1661c		signal peptidase II	1.24	0.003	257.51	207.51
SMU_1662	holB	DNA polymerase III subunit delta'	1.02	0.830	211.14	206.93
SMU_1663	kthY	thymidylate kinase	1.44	0.000	319.43	221.1
SMU_1664c		acetoin utilization protein, acetoin dehydrogenase	1.32	0.000	379.74	288.45
SMU_1665	livF	ABC transporter ATP-binding protein	1.38	0.000	853.09	616.55
SMU_1666	livG	ABC transporter ATP-binding protein	1.18	0.000	888.42	752.88
SMU_1667	livM	branched chain amino acid ABC transporter permease	1.23	0.000	811.5	661.9
SMU_1668	livH	branched chain amino acid ABC transporter permease	1.12	0.002	875.35	778.78
SMU_1669	livK	branched chain amino acid ABC transporter substrate-binding protein	0.79	0.000	899.71	1133.52
SMU_1670c		hypothetical protein	0.99	1.000	158.98	160
SMU_1671c		hypothetical protein	1.09	0.260	202.5	185.19
SMU_1672	clpP	ATP-dependent Clp protease proteolytic subunit	0.77	0.000	776.07	1002.95
SMU_1673	upp	uracil phosphoribosyltransferase	0.93	0.770	310.29	335.15
SMU_1674	metC	aminotransferase	1.63	0.000	96.1	58.79
SMU_1675	metB	cystathionine gamma-synthase	1.42	0.016	67.03	47.16
SMU_1676c		hypothetical protein	1.15	0.120	161.23	140.16
SMU_1677	murE	UDP-N-acetylmuramoylalanyl-D-glutamate--L-lysineligase	0.81	0.041	176.35	216.76
SMU_1678		acyl-CoA thioesterase	0.98	0.930	344.47	352.64
SMU_1679c		hypothetical protein	0.93	0.780	322.68	346.22
SMU_1680c		hypothetical protein	0.94	0.750	348.73	372.77
SMU_1681c		hypothetical protein	0.90	0.570	429.72	475.79
SMU_1682c		intracellular protease	1.08	0.810	27.8	25.69
SMU_1683c		hypothetical protein	0.88	0.820	85.61	97.47
SMU_1685c		hypothetical protein	1.00	1.000	71.24	71.03
SMU_1687	ppaC	manganese-dependent inorganic pyrophosphatase	0.88	0.310	739.3	839.04
SMU_1688	dltD	extramembranal protein, DltD protein	1.25	0.000	414.81	331.65
SMU_1689	dltC	D-alanine--poly(phosphoribitol) ligase subunit 2	1.48	0.000	441.26	298.79
SMU_1690	dltB	hypothetical protein	1.14	0.038	319.92	280.39
SMU_1691	dltA	D-alanine--poly(phosphoribitol) ligase subunit 1	0.84	0.037	480.02	568.21
SMU_1692	pflA	pyruvate-formate lyase activating enzyme	0.76	0.000	382.02	504.63
SMU_1693	hlyX	hemolysin	0.70	0.000	544.8	782.75
SMU_1694c		permease	1.68	0.038	27	16.11
SMU_1695		molybdenum ABC transporter ATP-binding protein	0.78	0.001	226.2	288.56
SMU_1697c		hypothetical protein	0.58	0.000	123.76	214.72
SMU_1699c		hypothetical protein	1.00	1.000	339.36	340.68
SMU_1700c		LrgB family protein	1.39	0.004	113.59	81.77
SMU_1701c		hypothetical protein		0.000	8.97	0
SMU_1702c		phosphatase	1.38	0.000	197.05	142.94
SMU_1703c		hypothetical protein	0.82	0.020	255.47	312.7
SMU_1704		hypothetical protein	0.69	0.130	22.43	32.63
SMU_1705		hypothetical protein	0.53	0.000	72.64	138.23
SMU_1706		hypothetical protein	0.88	0.900	66.12	75.44

SMU_1707c		rRNA methylase	0.73	0.001	137.34	188.15
SMU_1708	trkA	potassium transporter peripheral membrane protein	0.82	0.039	216.8	264.1
SMU_1709	trkH	potassium uptake protein TrkH	0.82	0.076	164.85	200.28
SMU_1710c		hypothetical protein	1.06	0.530	192.03	181.89
SMU_1711	rluB	pseudouridylate synthase	1.11	0.044	478.87	431.37
SMU_1712c		segregation and condensation protein B	1.00	1.000	228.84	228.08
SMU_1713c		segregation and condensation protein A	0.97	0.960	231.13	237.94
SMU_1714c		site-specific tyrosine recombinase XerD	1.30	0.005	156.19	120.49
SMU_1715c		hypothetical protein	1.95	0.000	177.55	90.85
SMU_1716c		hypothetical protein	1.07	0.340	255.44	238.24
SMU_1717c		deoxyribonucleotide triphosphate pyrophosphatase/hypothetical protein	1.49	0.000	165.19	110.57
SMU_1718	murI	glutamate racemase	1.06	0.240	460.12	433.29
SMU_1719c		hypothetical protein	0.97	0.860	610.12	630.26
SMU_1721c		diaminopimelate decarboxylase	0.90	0.620	292.72	324.36
SMU_1722c		hypothetical protein	0.59	0.000	236.45	403.54
SMU_1723c		hypothetical protein	0.90	0.480	544.49	603.57
SMU_1724c		rRNA methylase	0.65	0.001	72.03	110.41
SMU_1725		acylphosphatase	0.77	0.620	11.42	14.79
SMU_1727		OxaA-like protein precursor	0.62	0.000	663.71	1070.69
SMU_1728	greA	transcription elongation factor GreA	0.68	0.000	208.96	308.72
SMU_1729c		aminodeoxychorismate lyase	1.09	0.210	243.28	222.25
SMU_1730c		acetyltransferase	1.04	0.790	95.25	91.39
SMU_1732c		hypothetical protein	1.06	0.430	255.19	240.98
SMU_1733c		SNF helicase	0.79	0.027	124.49	157.23
SMU_1734	accA	acetyl-CoA carboxylase carboxyl transferase subunit alpha	1.21	0.000	1254.28	1037.3
SMU_1735	accD	acetyl-CoA carboxylase subunit beta	1.14	0.000	2023.37	1782.2
SMU_1736	accC	acetyl-CoA carboxylase biotin carboxylase subunit	1.16	0.000	1422.41	1221.58
SMU_1737	fabZ	(3R)-hydroxymyristoyl-ACP dehydratase	1.34	0.000	1723.93	1287.25
SMU_1739	fabF	3-oxoacyl-ACP synthase	1.16	0.000	1327.04	1140.32
SMU_1740	fabG	3-ketoacyl-ACP reductase	0.96	0.740	1229.65	1275.88
SMU_1741	fabD	malonyl CoA-ACP transacylase	1.00	0.980	1170.09	1168.37
SMU_1742c		trans-2-enoyl-ACP reductase	1.73	0.000	963.5	556.75
SMU_1743	acp	acyl carrier protein	1.37	0.000	224.28	163.15
SMU_1744	fabH	3-oxoacyl-ACP synthase III	0.74	0.000	763.32	1028.26
SMU_1745c		transcriptional regulator	0.62	0.000	524.53	847.8
SMU_1746c		enoyl-CoA hydratase	0.57	0.000	1128.77	1967.86
SMU_1747c		phosphatase	0.73	0.012	80.06	108.97
SMU_1748	akh	aspartate kinase	0.88	0.640	225.86	255.87
SMU_1750c		hypothetical protein	1.51	0.000	181.43	120.49
SMU_1752c		hypothetical protein	3.51	0.000	105.12	29.96
SMU_1753c		hypothetical protein	2.76	0.000	169.91	61.47
SMU_1754c		hypothetical protein	1.47	0.046	45.79	31.25
SMU_1755c		hypothetical protein	2.70	0.000	201.12	74.46
SMU_1757c		hypothetical protein	3.03	0.000	178.31	58.91
SMU_1758c		hypothetical protein	2.80	0.000	160.13	57.13
SMU_1760c		hypothetical protein	3.68	0.000	104.12	28.26
SMU_1761c		hypothetical protein	2.42	0.000	39.99	16.55
SMU_1762c		hypothetical protein	0.52	0.002	23.67	45.66
SMU_1763c		hypothetical protein	3.15	0.000	83.1	26.37
SMU_1764c		hypothetical protein	2.98	0.000	42.16	14.17
SMU_1765c		hypothetical protein	0.36	0.000	17.34	48.45
SMU_1766c		hypothetical protein	1.98	0.000	204.76	103.22
SMU_1767c		hypothetical protein	1.07	0.310	303.6	284.56
SMU_1768c		hypothetical protein	1.06	0.340	328.22	309.21
SMU_1770	syv	valyl-tRNA synthetase	1.07	0.190	534.66	501.53
SMU_1771c		hypothetical protein	0.73	0.320	14.71	20.26
SMU_1772c		hypothetical protein	0.76	0.140	36.94	48.46
SMU_1773c		hypothetical protein	1.28	0.250	37.4	29.19
SMU_1774c		hypothetical protein	0.31	0.000	243	774.28
SMU_1775c		hypothetical protein	0.97	0.960	100.73	104.34
SMU_1776c		hypothetical protein	0.46	0.000	27.51	60.16
SMU_1777	nrdI	flavoprotein NrdI	0.86	0.760	65.21	75.45
SMU_1779c		RNA methyltransferase	0.89	0.800	80.51	90.22
SMU_1780		recombination regulator RecX	0.71	0.150	24.58	34.79
SMU_1781		hypothetical protein	0.58	0.000	448.16	768.69
SMU_1782		hypothetical protein	0.61	0.000	79.83	130.76
SMU_1783	proS	prolyl-tRNA synthetase	1.10	0.064	452.54	411.09
SMU_1784c		Eep protein-like protein	1.22	0.005	263.92	215.79
SMU_1785	cdsA	phosphatidate cytidylyltransferase synthase	0.92	0.700	274.76	299.06
SMU_1786	uppS	undecaprenyl pyrophosphate synthase	0.97	0.970	334.3	342.99
SMU_1787c		preprotein translocase subunit YajC	0.80	0.000	541.64	676.57
SMU_1788c		bacterocin transport accessory protein, Bta	0.63	0.000	214.97	341.81
SMU_1789c		hypothetical protein	1.08	0.160	363.43	334.97
SMU_1790c		transcriptional regulator	4.97	0.000	150.16	30.2
SMU_1791c		hypothetical protein	1.78	0.000	220.31	123.55
SMU_1792c		hypothetical protein	1.84	0.000	289.35	157
SMU_1794c		hypothetical protein	2.65	0.000	254.38	96.08
SMU_1795c		hypothetical protein	1.01	0.950	141.95	140.37
SMU_1797c		hypothetical protein	2.13	0.000	189.72	89.23
SMU_1798c		hypothetical protein	1.44	0.000	257.94	179.65
SMU_1799	nadD	nicotinic acid mononucleotide adenylyltransferase	1.40	0.000	580.23	414.71
SMU_1800c		hypothetical protein	1.59	0.000	584.01	367.91
SMU_1801c		GTPase YqeH	1.76	0.000	412.3	234.43
SMU_1802c		hypothetical protein	0.79	0.000	791.79	997.04
SMU_1803c		hypothetical protein	0.88	0.710	124.59	141.36
SMU_1804c		hypothetical protein		0.000	10.71	0
SMU_1805		transcriptional regulator	1.39	0.330	18.51	13.34
SMU_1806		glycosyltransferase	1.62	0.032	30.81	18.97
SMU_1807c		permease	1.52	0.006	65.66	43.2
SMU_1808c		integrase fragment	0.18	0.000	11.48	65.21
SMU_1809	scnG	bacteroiocin operon protein ScnG-like protein	0.92	1.000	45.68	49.57

SMU_1810	scnE	bacteriocin operon component, ScnE-like protein	1.55	0.180	19.35	12.47
SMU_1811	scnF	bacteriocin component ScnF-like protein	1.52	0.052	33.12	21.76
SMU_1812		transposase, ISSmu2	1.15	0.052	262.53	228.83
SMU_1813		transposase fragment	1.07	0.360	253.71	237.14
SMU_1814	scnK	histidine kinase, ScnK-like protein	0.96	0.960	127.76	133.42
SMU_1815	scnR	response regulator; ScnR-like protein	0.82	0.044	212.11	257.89
SMU_1816c		maturase-like protein	0.85	0.570	43.2	51.07
SMU_1817c		maturase-like protein	1.46	0.018	62.14	42.62
SMU_1818c		hypothetical protein	0.82	0.310	50.86	62.15
SMU_1819	gatB	aspartyl/glutamyl-tRNA amidotransferase subunit B	1.10	0.040	628.53	572.98
SMU_1820c		aspartyl/glutamyl-tRNA amidotransferase subunit A	1.03	0.640	458.73	447.21
SMU_1821c		aspartyl/glutamyl-tRNA amidotransferase subunit C	1.35	0.000	260.72	192.52
SMU_1822	gatA	aspartyl-tRNA synthetase	1.46	0.000	199.69	136.43
SMU_1823	pncA	pyrazinamidase/nicotinamidase	1.22	0.000	963.15	792.59
SMU_1824c		transcriptional repressor CodY	0.71	0.000	616.96	872.69
SMU_1826	yfbQ	aminotransferase	0.94	0.830	236.51	252.32
SMU_1827		biotin biosynthesis protein	0.90	1.000	42.48	47.16
SMU_1828		hypothetical protein	0.52	0.000	1089.69	2101.97
SMU_1830c		hypothetical protein	1.18	0.027	238.22	202.09
SMU_1831	aspG	L-asparaginase	1.05	0.430	313.8	298.5
SMU_1832		hypothetical protein	0.71	0.000	167.7	234.55
SMU_1833	recG	ATP-dependent DNA helicase, RecG	1.10	0.350	124.02	112.64
SMU_1834	alr	alanine racemase	1.02	0.560	736.9	719.56
SMU_1835	acpS	4'-phosphopantetheinyl transferase	1.98	0.000	413.92	208.67
SMU_1836	aroG	phospho-2-dehydro-3-deoxyheptonate aldolase	1.11	0.012	748.49	674.98
SMU_1837	aroH	phospho-2-dehydro-3-deoxyheptonate aldolase	1.27	0.008	169.01	132.91
SMU_1838	secA	preprotein translocase subunit SecA	1.09	0.023	906.49	833.13
SMU_1839	manA	mannose-6-phosphate isomerase	1.10	0.023	678.86	615.8
SMU_1840	scrK	fructokinase	1.08	0.120	438.5	404.26
SMU_1841	scrA	PTS system sucrose-specific transporter subunit IIABC	0.86	0.530	114.59	132.51
SMU_1843	scrB	sucrose-6-phosphate hydrolase	0.61	0.000	93.7	152.36
SMU_1844	scrR	sucrose operon repressor	0.90	0.830	110.62	122.33
SMU_1845	nusB	transcription antitermination protein NusB	0.83	0.063	230.86	277.92
SMU_1846c		hypothetical protein	1.40	0.000	474.05	338.19
SMU_1847	efp	elongation factor P	1.19	0.000	858.2	721.56
SMU_1848		hypothetical protein	1.02	0.920	86.23	84.56
SMU_1849	comEB	deoxycytidylate deaminase	1.11	0.110	307.75	277.97
SMU_1850	pepP	aminopeptidase	1.26	0.000	445.48	354.03
SMU_1851	uvrA	excinuclease ABC subunit A	0.99	0.970	395.56	400.42
SMU_1852		magnesium/cobalt transport protein	0.57	0.000	251.47	442.95
SMU_1853		hypothetical protein	1.24	0.006	222.82	180.26
SMU_1854		hypothetical protein	0.49	0.000	234.77	483.83
SMU_1855		hypothetical protein	0.51	0.000	144.56	281.04
SMU_1856c		hypothetical protein	0.50	0.000	152.11	305.91
SMU_1858	rs18	30S ribosomal protein S18	0.77	0.000	5557.91	7178.68
SMU_1859	ssb	single-stranded DNA-binding protein	0.62	0.000	6580.26	10624.52
SMU_1860	rs6	30S ribosomal protein S6	0.55	0.000	3939.52	7176.64
SMU_1861c		hypothetical protein	0.95	1.000	84.42	89.07
SMU_1862		hypothetical protein	0.85	0.570	73.85	86.48
SMU_1865	mutY	A/G-specific adenine glycosylase	1.01	0.960	133.2	132.1
SMU_1867c		alcohol dehydrogenase	0.62	0.000	584.08	945.56
SMU_1869	trxA	thioredoxin	0.34	0.000	831.41	2439.56
SMU_1870	mutS2	DNA mismatch repair protein MutS2	1.16	0.092	168.24	144.96
SMU_1871c		hypothetical protein	0.68	0.001	84.07	122.99
SMU_1872c		hypothetical protein	0.44	0.000	189.85	430.56
SMU_1873	rnh3	ribonuclease HIII	0.91	0.660	292.86	322.16
SMU_1874	lepC	signal peptidase I	0.93	0.750	229.81	248.02
SMU_1875		exodeoxyribonuclease V	0.79	0.061	78.19	99.43
SMU_1876		hypothetical protein	1.07	0.650	98.04	91.84
SMU_1877	ptnA	PTS system mannose-specific transporter subunit IIAB	0.93	0.690	401.62	432
SMU_1878	ptnC	PTS system mannose-specific transporter subunit IIC	1.31	0.000	663.34	506.24
SMU_1879		PTS system mannose-specific transporter subunit IID	1.15	0.002	578.32	500.83
SMU_1881c		ABC transporter ATP-binding protein	2.36	0.000	102.81	43.58
SMU_1882c		hypothetical protein	0.38	0.000	35.34	92.44
SMU_1883		hypothetical protein	0.59	0.000	186.43	316.17
SMU_1884c		hypothetical protein	1.53	0.009	55.81	36.57
SMU_1886	sys	seryl-tRNA synthetase	0.96	0.870	421.27	438.95
SMU_1888		transposase fragment	1.47	0.490	10.07	6.87
SMU_1889c		hypothetical protein	0.83	0.440	52.3	62.88
SMU_1891c		hypothetical protein	0.14	0.000	6.55	45.88
SMU_1892c		hypothetical protein	0.22	0.000	5.61	25.25
SMU_1893c		transposase, ISSmu1	2.03	0.009	25.19	12.41
SMU_1894c		hypothetical protein	0.93	1.000	13.95	15.08
SMU_1895c		hypothetical protein	4.98	0.000	113.63	22.83
SMU_1897		ABC transporter ATP-binding protein	0.80	0.008	207.9	259.52
SMU_1898		ABC transporter ATP-binding protein/permease	0.82	0.130	119.62	146.23
SMU_1899		ABC transporter ATP-binding protein/permease (fragment)	0.90	0.890	95.99	106.15
SMU_1900		hypothetical protein	1.35	0.018	92.05	68.33
SMU_1902c		hypothetical protein	0.97	0.790	1243.61	1280.53
SMU_1903c		hypothetical protein	0.86	0.013	1650.54	1909.17
SMU_1904c		hypothetical protein	0.95	0.190	6220.38	6571.78
SMU_1905c		bacteriocin secretion protein	1.29	0.000	9956.29	7692.22
SMU_1906c		hypothetical protein	1.73	0.000	12216.85	7050.59
SMU_1907		hypothetical protein	1.17	0.000	12556.56	10693.72
SMU_1908c		hypothetical protein	1.79	0.000	1007.86	562.23
SMU_1909c		hypothetical protein	0.94	0.065	10457.26	11092.31
SMU_1910c		hypothetical protein	1.25	0.000	7525.5	6019.85
SMU_1912c		hypothetical protein	1.15	0.000	7682.42	6676.26
SMU_1913c		immunity protein, BLpL-like	0.77	0.000	4832.79	6310.62
SMU_1914c	cipB	hypothetical protein	3.53	0.000	5795.99	1642.08
SMU_1915	comC	competence stimulating peptide	0.67	0.000	261.1	391.53

SMU_1916	comD	histidine kinase of the competence regulon, ComD	0.74	0.000	937.36	1275.06
SMU_1917	comE	response regulator of the competence regulon ComE	0.59	0.000	1705.66	2891.54
SMU_1918	dedA	membrane-associated protein DedA	1.02	0.760	286.77	279.88
SMU_1919	sapR2	hypothetical protein	0.88	0.280	791.03	896.58
SMU_1920	pgdA	GTP-binding protein Der	0.97	0.820	1018.89	1050.78
SMU_1921	dnaI	primosomal protein DnaI	0.95	0.790	506.14	530.71
SMU_1922	dnaB	chromosome replication protein	0.85	0.160	232.77	274.71
SMU_1923c		transcriptional regulator NrdR	0.81	0.011	252.9	311.75
SMU_1924	gcrR	response regulator GcrR for glucan-binding protein C	0.90	0.480	602.47	666.85
SMU_1925c		hypothetical protein	1.10	0.280	155.17	140.43
SMU_1926	psaR	transcriptional regulator	1.49	0.000	145.89	97.82
SMU_1927		ABC transporter ATP-binding protein	1.26	0.003	210.08	166.2
SMU_1928	psaB	ABC transporter permease	0.94	0.930	150.03	160.14
SMU_1929	htpX	heat shock protein HtpX	0.85	0.011	830.37	979.06
SMU_1930	lemA	cytoplasmic membrane protein	0.59	0.000	619.26	1045.67
SMU_1931	gidB	16S rRNA methyltransferase GidB	0.93	1.000	45.78	49.42
SMU_1933c		cobalt permease	0.76	0.052	62.37	81.89
SMU_1934c		cobalt ABC transporter ATP-binding protein	1.45	0.000	134.42	92.73
SMU_1935c		hypothetical protein	1.17	0.280	72.86	62.02
SMU_1936c		hypothetical protein	1.30	0.019	112.89	86.95
SMU_1937	cnhA	carbon-nitrogen hydrolase	0.74	0.007	97.06	131.66
SMU_1938c		ABC transporter permease	1.40	0.008	86.1	61.44
SMU_1939c		ABC transporter ATP-binding protein	1.08	0.600	95.6	88.84
SMU_1940c		peptidase, AtmC; ArgE/DapE/Acy1 family protein	1.53	0.002	78.48	51.36
SMU_1941	atmB	membrane lipoprotein	0.96	1.000	78.99	82.7
SMU_1942c		amino acid binding protein	0.77	0.020	91.48	118.93
SMU_1943	syl	leucyl-tRNA synthetase	0.87	0.210	630.76	722.56
SMU_1945		hypothetical protein	1.75	0.000	109.36	62.49
SMU_1946		hypothetical protein	1.27	0.004	211.83	167.44
SMU_1947	nusG	transcription antitermination protein NusG	0.74	0.000	285.73	385.78
SMU_1948	secE	preprotein translocase subunit SecE	1.03	0.660	267.32	258.84
SMU_1949	pbp2a	membrane carboxypeptidase, penicillin-binding protein 2a	1.16	0.041	237.22	203.72
SMU_1950		pseudouridylate synthase	0.96	0.990	84.58	87.96
SMU_1951c		hypothetical protein	0.90	1.000	36.5	40.74
SMU_1954	groEL	molecular chaperone GroEL	1.36	0.000	2509.01	1841.53
SMU_1955	groES	co-chaperonin GroES	1.54	0.000	268.63	173.9
SMU_1956c		hypothetical protein	1.09	0.640	60.59	55.58
SMU_1957		PTS system mannose-specific transporter subunit IID	0.91	0.730	272.29	298.16
SMU_1958c		PTS system mannose-specific transporter subunit IIC	0.97	0.940	328.88	340.68
SMU_1960c		PTS system mannose-specific transporter subunit IIB	0.90	0.660	289.04	320.78
SMU_1961c		PTS system sugar-specific transporter subunit IIA	2.14	0.000	95.92	44.79
SMU_1963c		sugar-binding periplasmic protein	0.59	0.000	175.9	298.92
SMU_1964c		response regulator	0.65	0.000	295.08	451.92
SMU_1965c		histidine kinase	0.82	0.038	236.38	286.93
SMU_1966c		periplasmic sugar-binding protein	0.61	0.000	224.42	370.7
SMU_1967	ssb2	single-stranded DNA-binding protein	0.51	0.000	20824.55	40730.48
SMU_1968c		hypothetical protein	2.18	0.000	63.41	29.09
SMU_1969c		transcriptional regulator	0.35	0.000	15.2	43.15
SMU_1970c		phenylalanyl-tRNA synthetase subunit beta	0.88	0.730	169.15	191.3
SMU_1971c		thioredoxin	0.66	0.000	165.79	249.64
SMU_1972c		hypothetical protein	0.65	0.000	144.85	221.94
SMU_1973	pepA	glutamyl-aminopeptidase; endo-1,4-beta-glucanase	0.63	0.000	165.78	262.72
SMU_1974	proC	pyrroline-5-carboxylate reductase	0.69	0.000	294.79	430.35
SMU_1975c		hypothetical protein	0.79	0.000	285.23	362.25
SMU_1976c		hypothetical protein	0.70	0.000	307.04	440.39
SMU_1977c		transcriptional regulator	0.97	0.860	841.01	864.68
SMU_1978	ackA	acetate kinase	0.94	0.052	10660.93	11338.85
SMU_1979c		hypothetical protein	0.84	0.000	3742.26	4480.51
SMU_1980c		hypothetical protein	0.93	0.100	5536.83	5950.63
SMU_1981c		hypothetical protein	1.00	0.800	3759.82	3743.13
SMU_1982c		hypothetical protein	0.90	0.064	3045.25	3397.32
SMU_1983	comYD	competence protein ComYD	1.07	0.002	2803.07	2625.28
SMU_1984	comYC	competence protein ComYC	1.59	0.000	1699.78	1071.85
SMU_1985	comYB	ABC transporter ComYB	0.92	0.100	4192.74	4550.48
SMU_1987	comYA	ABC transporter ATP-binding protein ComYA; late competence gene	1.09	0.000	2628.8	2422.1
SMU_1988c		DNA binding protein	0.35	0.000	252.28	729.94
SMU_1989	rpoC	DNA-directed RNA polymerase subunit beta'	1.27	0.000	1720.03	1357.99
SMU_1990	rpoB	DNA-directed RNA polymerase subunit beta	1.47	0.000	1135.21	771.85
SMU_1991	pbp1b	membrane carboxypeptidase, penicillin-binding protein 1b	0.95	0.850	274.36	288.19
SMU_1992	tyrS	tyrosyl-tRNA synthetase	1.09	0.280	199.02	182.98
SMU_1993	adcB	ABC transporter zinc permease	0.69	0.033	44.8	64.76
SMU_1994	adcC	ABC transporter ATP-binding protein	0.49	0.000	119.68	245.54
SMU_1995c		transcriptional regulator	0.50	0.000	59	118.09
SMU_1996	ipk	4-diphosphocytidyl-2C-methyl-D-erythritol kinase	1.08	0.320	246.19	228.92
SMU_1997	sigX	ComX, transcriptional regulator of competence-specific genes	4.22	0.000	131.6	31.22
SMU_1999c		hypothetical protein	0.82	0.370	49.9	61.03
SMU_2000	rl17	50S ribosomal protein L17	0.78	0.000	1820.42	2335.32
SMU_2001	rpoA	DNA-directed RNA polymerase subunit alpha	1.35	0.000	2136.31	1580.69
SMU_2002	rs11	30S ribosomal protein S11	1.16	0.000	4008.93	3442.17
SMU_2003	rs13	30S ribosomal protein S13	0.90	0.053	3378.77	3761.9
SMU_2003a		50S ribosomal protein L36	0.86	0.007	1387.73	1619.82
SMU_2004	if1	translation initiation factor IF-1	0.94	0.620	889.56	946.59
SMU_2005	adk	adenylate kinase	1.28	0.000	365.8	285.9
SMU_2006	secY	preprotein translocase subunit SecY	0.92	0.360	1542.95	1670.52
SMU_2007	rl15	50S ribosomal protein L15	1.55	0.000	1933.35	1245.36
SMU_2008	rl30	50S ribosomal protein L30	0.90	0.080	3259.32	3602.12
SMU_2009	rs5	30S ribosomal protein S5	1.24	0.000	3063.96	2465.92
SMU_2010	rl18	50S ribosomal protein L18	1.32	0.000	5868.07	4439.02
SMU_2011	rl6	50S ribosomal protein L6	1.45	0.000	2431.21	1675.05
SMU_2012	rs8	30S ribosomal protein S8	0.92	0.240	1942.76	2123.16
SMU_2014	rs14	30S ribosomal protein S14	0.98	0.590	5303	5438.05

SMU_2015	rl5	50S ribosomal protein L5	1.14	0.000	3543.58	3098.74
SMU_2016	rl24	50S ribosomal protein L24	1.31	0.000	3471.92	2646.15
SMU_2017	rl14	50S ribosomal protein L14	1.22	0.000	4486.13	3682.34
SMU_2018	rs17	30S ribosomal protein S17	1.18	0.000	2831.33	2393.16
SMU_2019	rl29	50S ribosomal protein L29	1.36	0.000	2768.44	2031.91
SMU_2020	rl16	50S ribosomal protein L16	1.24	0.000	4843.39	3915.48
SMU_2022	rl22	50S ribosomal protein L22	1.19	0.000	4348.56	3656.01
SMU_2025	rl3	50S ribosomal protein L3	1.10	0.000	4309.81	3903.31
SMU_2026c		30S ribosomal protein S10	0.78	0.000	4579.91	5891.21
SMU_2027		transcriptional regulator	0.49	0.000	106.66	215.8
SMU_2028	sacB	beta-D-fructosyltransferase	0.85	0.340	139.77	163.49
SMU_2029	clpC	class III stress response-related ATP-dependent Clp protease, ATP-bindi	1.07	0.210	461.7	432.61
SMU_2030	ctsR	CtsR family transcriptional regulator	0.66	0.000	327.3	499.06
SMU_2031	eftS	elongation factor Ts	0.81	0.000	1459.44	1805.49
SMU_2032	rs2	30S ribosomal protein S2	0.77	0.000	2087.64	2714.56
SMU_2033c		hypothetical protein	0.62	0.000	68.42	109.59
SMU_2035		bacteriocin immunity protein	0.72	0.000	258.64	358.9
SMU_2036	pepO	peptidase	0.82	0.000	824.09	1009.48
SMU_2037	treA	trehalose-6-phosphate hydrolase TreA	1.09	0.140	376.76	346.14
SMU_2038	pttB	PTS system trehalose-specific transporter subunit IIABC	0.99	1.000	284.34	286.52
SMU_2040	treR	transcriptional regulator; repressor of the trehalose operon	0.98	1.000	72.38	74.13
SMU_2042	dexA	dextranase	1.47	0.000	560.82	381.64
SMU_2043c		D-tyrosyl-tRNA(Tyr) deacylase	1.37	0.000	190.37	139.41
SMU_2044	relA	stringent response protein, ppGpp synthetase	1.02	0.900	177.05	174.22
SMU_2046c		hypothetical protein	0.86	0.570	111.61	129.47
SMU_2047	ptsG	PTS system glucose-specific transporter subunit IIABC	0.90	0.560	355.26	395.01
SMU_2048		hypothetical protein	0.36	0.000	105.15	292
SMU_2049c		16S rRNA (uracil(1498)-N(3))-methyltransferase	1.33	0.000	210.32	157.97
SMU_2050c		50S ribosomal protein L11 methyltransferase	0.99	1.000	183.86	186.43
SMU_2052c		hypothetical protein	0.52	0.000	51.62	99.46
SMU_2053c		hypothetical protein	0.98	1.000	31.51	32.13
SMU_2054c		hypothetical protein	2.54	0.000	107.31	42.29
SMU_2055		acetyltransferase	0.85	0.190	202.45	239.45
SMU_2056		recombination factor protein RarA	1.32	0.000	238.62	180.63
SMU_2057c		cadmium-transporting ATPase	0.77	0.000	427.27	552.75
SMU_2058		transcriptional regulator	1.02	0.980	44.23	43.31
SMU_2059c		hypothetical protein	1.23	0.200	63.22	51.58
SMU_2060		LysR family transcriptional regulator	0.59	0.000	169.47	287.81
SMU_2061		hypothetical protein	0.83	0.063	272.83	326.77
SMU_2063	hemZ	ferrochelatase	1.28	0.011	155.71	122.12
SMU_2064c		transmembrane protein	1.01	0.980	94.42	93.53
SMU_2065		UDP-glucose 4-epimerase	1.42	0.000	239.38	168.86
SMU_2066c		transmembrane protein	1.20	0.046	158.19	131.82
SMU_2067	csbB	stress response protein	0.80	0.040	133.65	166.44
SMU_2069		zinc transporter ZupT	2.41	0.000	31.12	12.89
SMU_2070		hypothetical protein	2.93	0.000	39.96	13.66
SMU_2071		anaerobic ribonucleotide reductase activating protein	1.25	0.190	60.26	48.37
SMU_2072c		acetyltransferase	1.31	0.140	52.09	39.77
SMU_2073c		hypothetical protein	1.67	0.000	75.54	45.27
SMU_2074	nrdD	anaerobic ribonucleoside triphosphate reductase	1.28	0.000	466.54	365.48
SMU_2075c		hypothetical protein	0.65	0.000	224.75	347.46
SMU_2076c		hypothetical protein	1.53	0.000	126.23	82.72
SMU_2077c		hypothetical protein	0.84	0.000	1059.68	1267.72
SMU_2078c		Holliday junction resolvase-like protein	0.77	0.000	2183.28	2852.4
SMU_2079c		hypothetical protein	0.66	0.000	1555.3	2356.07
SMU_2080		hypothetical protein	0.98	0.960	371.21	379.09
SMU_2081		hypothetical protein	1.53	0.000	825.76	540.79
SMU_2083c		hypothetical protein	0.83	0.000	1486.32	1790.87
SMU_2084c		transcriptional regulator Spx	0.53	0.000	3724.45	7074.12
SMU_2085	recA	recombinase A	0.98	0.720	6409.58	6508.49
SMU_2086	cinA	competence damage-inducible protein A	0.93	0.160	4511.68	4836.09
SMU_2087	tagI	3-methyladenine DNA glycosylase	1.00	1.000	53.08	53.01
SMU_2088	ruvA	Holliday junction DNA helicase RuvA	0.78	0.025	111.78	142.52
SMU_2089	hexB	DNA mismatch repair protein	0.73	0.009	80.98	111
SMU_2090c		hypothetical protein	1.14	0.026	374.69	329.2
SMU_2091c		DNA mismatch repair protein MutS	0.88	0.560	246.31	280.27
SMU_2092c		hypothetical protein	0.75	0.000	307.03	412.12
SMU_2093	argR	transcriptional regulator of arginine metabolism	0.71	0.000	147.05	208.23
SMU_2094c		hypothetical protein	0.69	0.130	22.04	32
SMU_2096c		hypothetical protein	1.17	0.710	13.29	11.38
SMU_2097		hypothetical protein	1.23	0.000	417.72	338.59
SMU_2098	argS	arginyl-tRNA synthetase	0.99	1.000	276.63	280.19
SMU_2099c		hypothetical protein	0.95	1.000	81.89	86.28
SMU_2100c		hypothetical protein	0.81	0.018	227.45	280.01
SMU_2101	aspS	aspartyl-tRNA synthetase	1.11	0.150	237.43	213.74
SMU_2102	hisS	histidyl-tRNA synthetase	0.83	0.014	369.88	446.7
SMU_2104		hypothetical protein	0.71	0.000	185.77	262.97
SMU_2104a		50S ribosomal protein L32	0.76	0.000	1819.39	2385.34
SMU_2105		hypothetical protein	0.39	0.000	642.12	1657.68
SMU_2106c		transcriptional regulator	0.84	0.490	55.65	66.17
SMU_2107c		hypothetical protein	0.61	0.000	97.61	160.72
SMU_2108c		transcriptional regulator	0.76	0.130	41.28	54.16
SMU_2109		MDR permease	0.77	0.046	67.65	88.35
SMU_2111c		hypothetical protein	1.64	0.010	41.5	25.25
SMU_2112	gbpA	glucan-binding protein GbpA	1.05	0.390	339.26	321.77
SMU_2113c		hypothetical protein	1.62	0.084	21.9	13.55
SMU_2114c		transcriptional regulator	1.44	0.064	39.44	27.31
SMU_2115		short-chain dehydrogenase	0.81	0.750	9.73	12.04
SMU_2116	opuCa	osmoprotectant amino acid ABC transporter ATP-binding protein	0.80	0.001	333.33	416.09
SMU_2117	opuCb	osmoprotectant ABC transporter permease	0.69	0.000	340.29	490.9
SMU_2118	opuCc	ABC transporter glycine betaine/carnitine/choline-binding protein	0.70	0.000	252.97	360.72

SMU_2119	opuCd	osmoprotectant ABC transporter permease	0.49	0.000	267.91	549.2
SMU_2120c		3-methyladenine DNA glycosylase	0.76	0.000	271.35	359.27
SMU_2121c		hypothetical protein	4.91	0.000	48.48	9.88
SMU_2123		hypothetical protein	0.76	0.097	48.44	63.77
SMU_2124		hypothetical protein	3.52	0.000	34.57	9.82
SMU_2125		hypothetical protein	1.18	0.260	68.59	58.24
SMU_2126c		purine-nucleoside phosphorylase	1.39	0.001	139.5	100.45
SMU_2127		succinate semialdehyde dehydrogenase	0.82	0.063	171.56	208.93
SMU_2128		dihydroxy-acid dehydratase	0.99	1.000	866.02	870.44
SMU_2129c		hypothetical protein	2.15	0.000	35.77	16.64
SMU_2130		hypothetical protein	0.64	0.110	17.03	26.8
SMU_2131		hypothetical protein	0.56	0.200	8.23	14.66
SMU_2133c		hypothetical protein	1.51	0.039	37.84	25.05
SMU_2134		transcriptional regulator	0.45	0.010	12.05	26.66
SMU_2135c		30S ribosomal protein S4	1.00	1.000	1208.37	1211.25
SMU_2136c		hypothetical protein	1.87	0.000	406.54	217.03
SMU_2137c		hypothetical protein	0.99	1.000	157.9	160
SMU_2138	dnaC	replicative DNA helicase	0.92	0.670	375.32	406.1
SMU_2139c		50S ribosomal protein L9	1.62	0.001	76.02	46.8
SMU_2142	rpiB	hypothetical protein	0.87	0.380	281.07	323.45
SMU_2143c		tRNA-specific 2-thiouridylase MnmA	0.87	0.290	351.06	404.66
SMU_2146c		hypothetical protein	1.20	0.000	776	648.95
SMU_2147c		hypothetical protein	0.65	0.000	440.64	679.08
SMU_2148c		cobalt permease	1.54	0.004	64.29	41.88
SMU_2149c		cobalt transporter ATP-binding subunit	1.25	0.012	182.83	146.67
SMU_2150c		cobalt transporter ATP-binding subunit	1.12	0.130	222.77	199
SMU_2151	pgsA	phosphotidylglycerophosphate synthase	0.75	0.072	52.19	69.7
SMU_2152c		hypothetical protein	1.06	0.650	117.83	111.15
SMU_2153c		peptidase	0.93	1.000	21.3	22.88
SMU_2154c		peptidase	0.40	0.000	20.36	50.61
SMU_2155		hypothetical protein	0.65	0.000	83.34	127.89
SMU_2156	recF	recombination protein F	0.77	0.045	70.4	91.6
SMU_2157	guaB	inosine 5'-monophosphate dehydrogenase	1.15	0.009	440.27	382.29
SMU_2158c		tryptophanyl-tRNA synthetase	0.68	0.000	125.61	185.26
SMU_2159		ABC-F family ATPase	0.93	0.810	178.36	191.99
SMU_2160		transmembrane protein	1.08	0.910	14.56	13.49
SMU_2161c		hypothetical protein	1.14	0.500	50.54	44.2
SMU_2162c		23S rRNA (pseudouridine(1915)-N(3))-methyltransferase RlmH	1.39	0.053	56.05	40.32
SMU_2164	htrA	serine protease HtrA	0.85	0.320	170.28	199.59
SMU_2165		SpoJ	1.17	0.440	47.01	40.07
SMU_72		hypothetical protein	1.23	0.062	109.96	89.25
SMU_96	rpoE	DNA-directed RNA polymerase subunit delta	1.14	0.018	438.05	385.23
SMU_801		GTP-binding protein	1.35	0.000	361.78	267.63
SMU_1127	rs20	30S ribosomal protein S20	0.13	0.000	82.83	636.08
SMU_1731	murC	UDP-N-acetyl muramate-alanine ligase	1.18	0.014	300.41	255.56
SMU_1738	bccP	biotin carboxyl carrier protein of acetyl-CoA carboxylase	1.23	0.000	1801.97	1467.46
SMU_2021	rs3	30S ribosomal protein S3	1.19	0.000	3150.76	2653.64
SMU_2023c		30S ribosomal protein S19	1.23	0.000	3051.64	2489.16
SMU_2167		50S ribosomal protein L2	1.17	0.000	3194.14	2739.42
SMU_2166		50S ribosomal protein L23	0.91	0.210	1986.81	2179.74
SMU_2024c		50S ribosomal protein L4	1.06	0.007	3069.91	2908.55

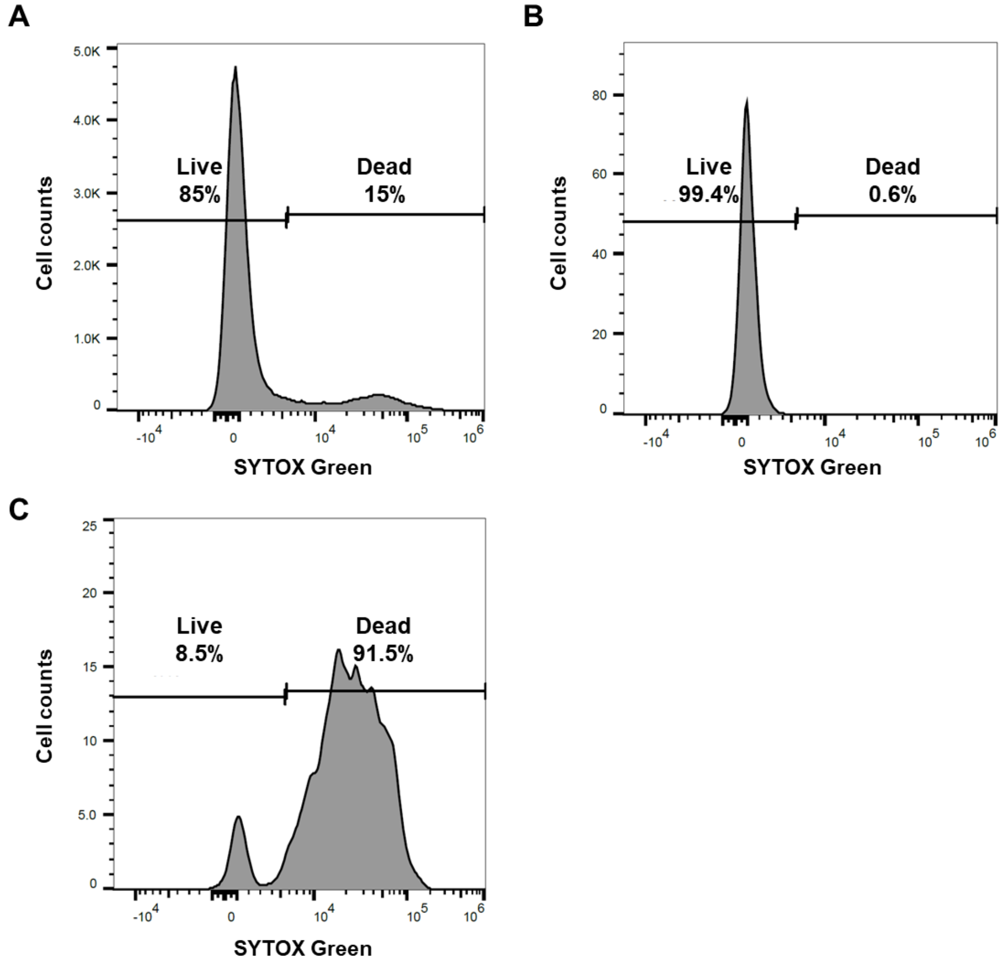

Fig. S1 Live/dead population analysis before and after cell sorting

(A) Cells were grown in BHI with sCSP in an aerobic atmosphere containing 5% CO_2 at 37 °C for 6 h. Dead cells were stained with SYTOX Green and the populations of SYTOX Green-negative and -positive cells were quantified by flow cytometry. The boundary between negative and positive of SYTOX Green signal was determined using unstained cells. (B and C) Collected cells from the sample shown in Fig. S1A were reanalyzed for confirmation. B and C show the results of tubes from which live and dead cells were collected, respectively.

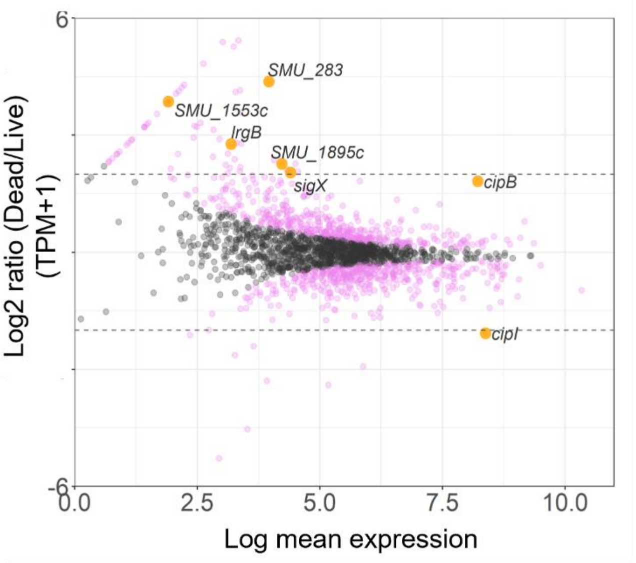

Fig. S2 RNA-seq results identify mRNAs that accumulated differentially between live and dead cells

The MA plot shows mRNAs that varied between live and dead cells. The vertical and horizontal axes correspond to log2 fold change (dead/live) and log-transformed mean of transcripts per million (TPM), respectively. Plots corresponding to genes with adjusted *P* values less than 0.05 are highlighted in magenta.

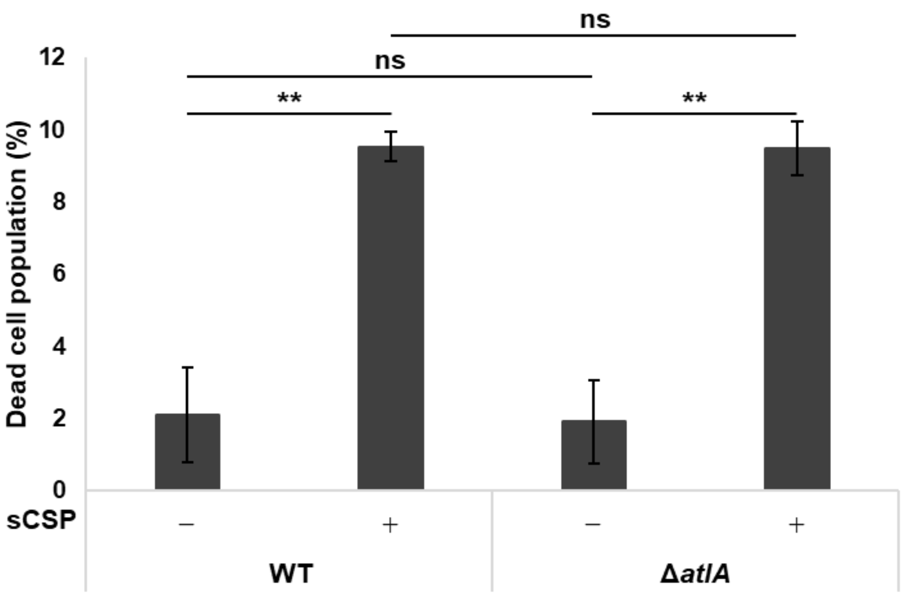

Fig. S3 Effect of *atlA* on CSP-dependent cell death

Cells were grown in BHI or BHI sCSP in an aerobic atmosphere containing 5% CO_2 at 37 °C for 6 h. Since the Δ*atlA* strain shows clumped chained cells, due to insufficient cell wall degradation and cell separation, cells were sonicated at 25 W for 60 seconds before staining to separate them into single cells. Dead cells were stained with SYTOX Green, and the population of SYTOX Green-positive cells was quantified by flow cytometry. Data indicate the mean ± standard deviation of the results from three independent experiments. ns indicates not significant.

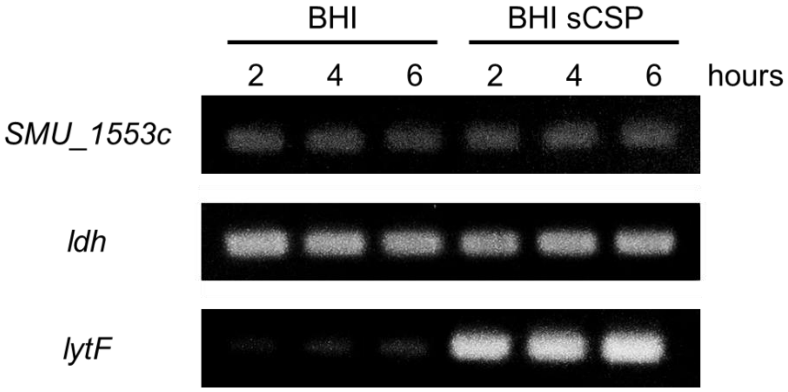

Fig. S4 *SMU_1553c* expression is not influenced by the growth phase and sCSP.

Cells were grown in BHI or BHI sCSP in an aerobic atmosphere containing 5% CO_2 at 37°C for 2, 4, or 6 h. Total RNA extracted from the cells was used for RT-PCR with gene-specific primers. As an endogenous control, *ldh* was used. *lytF* is a known gene that is upregulated by sCSP. A representative data from three independent experiments is shown.
